# The Influence of Age, Gender and Education on Neuropsychological Test Scores: Updated Clinical Norms for Five Widely Used Cognitive Assessments

**DOI:** 10.3390/jcm12165170

**Published:** 2023-08-08

**Authors:** Jannik F. Scheffels, Isabell Ballasch, Nadine Scheichel, Martin Voracek, Elke Kalbe, Josef Kessler

**Affiliations:** 1 Department of Psychology, University of Oldenburg, 26129 Oldenburg, Germany; 2 Department of Medical Psychology, Neuropsychology & Gender Studies, Center for Neuropsychological Diagnostics and Intervention (CeNDI), Faculty of Medicine and University Hospital Cologne, University of Cologne, 50937 Cologne, Germany; 3 Department of Cognition, Emotion, and Methods in Psychology, Faculty of Psychology, University of Vienna, 1010 Vienna, Austria

**Keywords:** sociodemographic effects, clinical normative data, Mini-Mental State Examination (MMSE), F-A-S Test (FAS), Rey–Osterrieth Complex Figure Test (ROCFT), Trail Making Test (TMT)

## Abstract

Background: Sociodemographic effects (i.e., age, gender, education) have been shown to influence neuropsychological test scores. The current retrospective, quasi-epidemiological work provides age-, gender- and education-corrected clinical norms for five common cognitive assessments. Methods: In total, test scores of 4968 patients from the University Hospital of Cologne (Department of Neurology), recruited between 2009 and 2020, were analyzed retrospectively. Conducted tests were the Mini-Mental State Examination (MMSE), F-A-S Test (FAS), Rey–Osterrieth Complex Figure Test (ROCFT) and Trail Making Test, Part A and B (TMT-A/-B). Using multiple linear regression analyses, test scores were analyzed for sociodemographic influences (age, gender, education). Based on these analyses, norms were generated by first separating patients into different age groups stratified by educational level and (if necessary) gender. Subsequently, percentile ranks and *z*-scores for a subsample including only individuals without dementia were calculated. Results: Lower age and higher educational level predicted better test scores (MMSE, FAS, ROCFT) and completion times (TMT-A/-B). Additionally, produced words on the FAS and remembered drawings from the ROCFT were influenced by gender, with females having better FAS but lower ROCFT (delayed recall) scores than males. Considering these effects, clinical norms were provided for the five cognitive assessments. Conclusions: We found influences of age, gender and education on test scores, although they are frequently not or only partially considered for test score interpretation. With the provided norms, neuropsychologists can make more profound evaluations of cognitive performance. A user-friendly Microsoft Excel file is offered to assist this process.

## 1. Introduction 

Due to their time- and cost-efficient properties, a variety of short cognitive screening instruments have become established in routine clinical practice. Although they do not replace extensive neuropsychological assessment, screening instruments often serve as a first step to quickly elaborating and characterizing cognitive deficits of patients. Further diagnostics can then be initiated if necessary. 

In order to achieve a proper interpretation of the individuals’ cognitive performance, the test scores of screening instruments should ideally be, among other psychometric requirements, independent of culture, language and education (that is, they should be free of sociodemographic effects) [1]. However, in clinical practice, it is known that this requirement is rarely met. Therefore, an appropriate neuropsychological test should at least provide up-to-date norms that are corrected for such effects. Otherwise, the risk of misinterpreting the patient’s cognitive performance is comparably high. In the worst case, such a misinterpretation may lead to false positive or false negative diagnoses, to ineffective therapy or to wrong medications. 

Unfortunately, sociodemographic effects on the subjects’ performance are frequently not or only partially considered by researchers and/or clinicians (such as physicians and (neuro)psychologists) although they are available [2]. This may be because at least some additional time investment is required (i.e., searching through various norm tables for the corresponding cutoff points). Another reason is that some screenings do not offer such corrected norms at all (i.e., sociodemographic effects were not accounted for in the test development stage). In the following, five widely used cognitive assessments (that are either used as part of a comprehensive neuropsychological battery or as stand-alone instruments) and sociodemographic effects on their test scores are discussed. 

### 1.1. Mini-Mental State Examination

The Mini-Mental State Examination (MMSE [3]; in its German adaptation [4]) is one of the most widely used screening instruments worldwide [1] and has been translated into various languages. Its time to administer usually is about five to ten minutes. Cutoff scores for dementia defined as 24 to 26 out of 30 possible points are commonly used (for a systematic review, see [2]). Higher scores are interpreted as normal cognitive functioning. The MMSE contains tasks assessing orientation in time and place, memory, calculation, visuoconstruction, language and apractic aspects. 

MMSE test scores have been shown to be influenced by age and education [5,6,7,8,9] with older and less educated patients achieving lower total scores. The individual’s gender, in contrast, seems not to affect MMSE scores [7,9,10]. Although sociodemographic effects are known and age and education corrections are available, they are usually not considered in clinical practice [11]. 

### 1.2. F-A-S Test

Another widely used instrument is the F-A-S Test (FAS), which is part of the Controlled Oral Word Association Test (COWAT) [12] and measures phonetic word fluency as well as executive functioning (see [13] for a discussion on its measured constructs). Individuals are instructed to name as many words as possible starting with the letters F, A, and S, consecutively. They are prohibited from saying proper nouns (e.g., Frank, Finland) or saying the same word with a different ending (e.g., accept, accepted). One minute per letter is provided and the number of generated words is added up to a total score. 

Age and education were shown to have a significant influence on test results of the FAS [14,15,16]. Patients with higher scores tend to be younger and higher educated. Regarding gender, some studies have found that women may slightly outperform men [17,18], whereas others did not find significant influences of gender on test scores of the FAS [19,20].

### 1.3. Rey–Osterrieth Complex Figure Test

The Rey–Osterrieth Complex Figure Test (ROCFT) [21,22] assesses visuospatial constructional abilities and visual memory. It can also be used for evaluating the patient’s planning strategies as well as organizational skills and, therefore, executive functions [23,24]. Many variations in the administration of this test have been reported (for an overview, see [24]). Commonly, patients are first instructed to copy a complex geometric figure presented horizontally on an A4 sheet of paper (ROCFT copy). No time constraint is given. After a 30 min time interval, the patient is asked to draw the figure from memory (ROFCT delayed recall). Based on the original scoring scheme by Osterrieth [21], both drawings are then evaluated by the examiner with regard to the position of the figure’s components and their accuracy. Each of the 18 components was given 2 points (accurately drawn and correctly placed), 1 point (accurately drawn but incorrectly placed; correctly placed but inaccurately drawn), 0.5 points (incorrectly placed but recognizable), or 0 points (inaccurately drawn and unrecognizable, or omitted). Thus, a total of 36 points can be achieved for each of the two drawings.

Similar to the other cognitive assessments, the test scores of the ROCFT copy and delayed recall are influenced by the patient’s age and education [25,26]. The influence of gender on ROCFT test scores is controversial, with some studies reporting men outperforming women [25,27,28] or vice versa [26]. 

### 1.4. Trail Making Test, Part A and B

The Trail Making Test (TMT) [29] consists of two conditions that are frequently conducted consecutively: TMT-A and TMT-B. The former consists of numbers from 1 to 25 that are pseudorandomized over an A4 sheet of paper. Patients are asked to connect them with a pencil in ascending order and as fast as possible. The TMT-B contains numbers (1 to 13) and letters (A to L) that should be connected alternately and in ascending/alphabetic order. Again, the time until task completion is recorded. Both conditions are preceded by a short example in order to ensure that instructions are followed properly during the real task. Usually, time is started at the moment when the participant starts to draw the first line. When a mistake is made, the participant is corrected while the time is still running. Additionally, some authors have proposed to handle a time constraint of 300 s in order to prevent the patient from being frustrated and to minimize the test’s duration [30,31].

The time needed to accomplish TMT-A is taken as a measure of visuomotor and processing speed [32,33], whereas TMT-B is supposed to assess visuomotor/processing speed but also subcomponents of executive functions such as cognitive flexibility and working memory [33,34,35]. The ratio score (time of completion for TMT-B/TMT-A) [36] accounts for basic cognitive functions such as general processing speed and is assumed to measure executive functions more specifically [37,38]. However, it does not appear to enhance clinical utility of the TMT [39]. The number of errors made is usually not considered for the tests’ evaluations.

Both conditions of the TMT seem to be influenced by the patient’s age [40,41,42,43,44], with increasing age being associated with longer time for completion. Conclusions on the influence of educational level on the TMT have been inconsistent between studies. Some studies found that both conditions are affected by educational level [41,43,45,46], with time of completion being longer for patients of lower educational level. However, another study only found such an influence of education on TMT-B [44]. Regarding gender, no or only small influences were found [42,43,44].

### 1.5. Aims of the Present Study

Based on the above findings and considerations, the present study set out (1) to explore possible sociodemographic effects (gender, age, education) on the test scores of five widely used neuropsychological assessments (i.e., the MMSE, FAS, ROCFT copy and 30 min delay, TMT-A, TMT-B) and (2) to offer updated and corrected clinical normative data for German-speaking individuals complaining about cognitive deficits but without dementia (with and without neurological diagnoses). Sociodemographic influences on cognitive tasks of German-speaking subjects were not expected to be substantially different compared to those from other western industrialized countries. However, the application of normative data based on samples closely resembling the main characteristics of the population assessed has been shown to be crucial for accurate determinations of neurocognitive impairment [47]. 

Overall, the present work aims to improve diagnostic accuracy and to prevent inaccurate or inappropriate diagnoses of patients’ cognitive performance. 

## 2. Methods 

### 2.1. Participants

**Figure 1:**
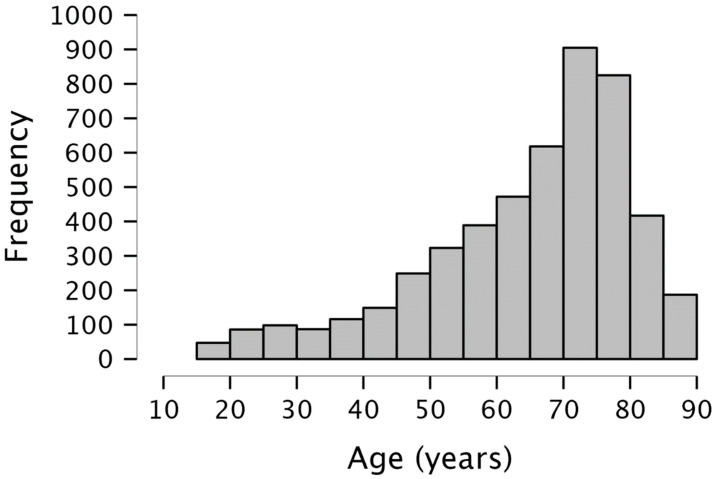
Age distribution of the entire sample (*n* = 4968).

In total, data of 4968 patients, assessed between 2009 and 2020, were obtained. They were in- and outpatients from the University Hospital of Cologne, Department of Neurology (urban setting). All patients included in the study were native German speakers. They all had in common that they came to the clinic because medical doctors, relatives, caregivers or themselves expected cognitive deficits that needed to be evaluated. Reasons for in-patient stays were subjective complaints about cognitive deficits and/or a referral by a general practitioner. Included patients were either without neurological diagnoses or suffered most frequently from the following diseases: idiopathic Parkinson’s disease, multiple sclerosis, epilepsy, normal pressure hydrocephalus, stroke, traumatic brain injury, dystonia, encephalitis, essential tremor, migraine, polyneuropathy, and transient global amnesia. All patients were assessed in the course of the routine neuropsychological examination, consisting of a standardized assessment (at least the tests listed below), supplemented by further tests depending on expected/declared cognitive deficits. Examiners were the neuropsychology work group’s staff (PhD clinical neuropsychologists) as well as students conducting projects for their master’s thesis (at least bachelor’s degree and after sufficient instruction). For evaluating cognitive performances, patients were examined on the same day with, in addition to other instruments, the DemTect [48], MMSE [3,4], FAS [12], ROCFT (copy and 30 min delay) [21,22], as well as TMT-A and -B [29]. The instructions were the same as those mentioned in the description of the tests in the Introduction. Sample characteristics are summarized in Table 1, the age distribution of the entire sample is depicted in Figure 1. For the categorization of educational level into ≥12 years and <12 years, the subject’s level of graduation was used. Hence, no graduation (less than 8 years of schooling), “Hauptschule” or “Volksschule” (8 or 9 years of education) and “Realschule” (10 years of education) were considered to be a lower educational level (<12 years), and “Gymnasium” (at least 12 or 13 years of education) was considered to be a higher educational level (≥12 years).

The test results of all assessed patients were routinely digitalized by the work group’s staff into a SPSS Statistics data file (Version 27) and were analyzed retrospectively in the current study. Therefore, no informed consent was needed. 

### 2.2. Statistical Analysis 

The following statistical analyses were performed with JASP, version 0.16 [49]. First of all, cases with missing data for sociodemographic characteristics (age, gender, education; *n* = 868) were removed from the original dataset (*n* = 5854). Then, outliers (*n* = 18; improbable data values and missing data entries) were removed in order to reduce dataset entries with typing errors and because some instruments did not have upper score boundaries (e.g., TMT-A/-B, FAS). For this purpose, the interquartile range (IQR), which is a measure of distribution variability, was used. It was calculated as the difference between the first (Q1) and third (Q3) quartiles. Any test scores greater than 1.5 times the IQR from Q3, or more than 1.5 times the IQR less than Q1, were considered outliers [50]. 

Then, means and standard deviations were calculated. In order to check for correlations between the neuropsychological tests, we calculated Spearman’s rank correlation coefficients. For investigating the effect of sociodemographics (age, gender, education) on neuropsychological test scores, we performed multiple linear regression analyses with and without bootstrapping after corresponding assumption checking (normality, independency and homoscedasticity of the residuals, multicollinearity of the predictors). Age, gender (male vs. female) and educational level (≥12 vs. <12 school years) were used as the independent variables and the corresponding instrument (MMSE, FAS, ROCFT copy and delayed recall, as well as TMT-A and -B) as the dependent variable. Due to nonnormally distributed test scores and missing homoscedasticity, we also conducted the same regression analyses with bootstrapping (5000 iterations) for increasing the robustness of our results [51].

In order to provide a more precise description of the relationship between neuropsychological test scores and age, we graphically illustrated the decline in cognitive performance across the lifespan with nonparametric bivariate local weighted error sums of squares (LOESS) smoothed plots [52]. LOESS curves with 95% confidence intervals were created with age in years as the independent variable and the corresponding test score as the dependent variable.

For generating clinical normative data, we first only selected patients who were classified using the DemTect (version A) [48] as having normal cognition (at least 13 out of 18 points, indicating age appropriate cognitive performance). The DemTect was chosen for this purpose because it is a commonly used instrument for the diagnosis of mild cognitive impairments (9 to 12 points) and dementia (0 to 8 points), it already offers norms that are corrected for age (≤40, <60, ≥60 and ≥80 years), and it has appropriate sensitivity and specificity [53]. Then, we divided the patients into different age groups (e.g., 18–29 years, 30–49 years, etc.). We decided to create age groups including at least ten patients after additionally separating into male/female and low/high educational level. 

Subsequently, because the data were not normally distributed, we first calculated percentile ranks for the test scores and then transformed them into *z*-scores, as suggested by Lienert and Raatz [54]. Percentile ranks were calculated with Microsoft Excel by using the following formula:

(1) $$PR=\frac{Cf-f/2}{N}\cdot 100$$

where f represents the frequency of a given test score in the sample and Cf is the cumulative frequency of that test score. Subsequently, the calculated percentile ranks were transformed (normalized) into corresponding *z*-equivalents (given in the *z*-score table) of the standard normal distribution by using the area under the normal curve ($\frac{Cf-f/2}{N}$). Percentile ranks of 16 (=1 SD) and above were considered as being age adequate cognitive performance.

## 3. Results 

### 3.1. Sociodemographics, Neuropsychological Test Results and Correlations

The patients’ sociodemographics and neuropsychological test scores are shown in Table 1, correlations of test scores are displayed in Table 2. 

### 3.2. Sociodemographic Effects on Test Scores 

In order to examine the influence of sociodemographics on test scores, we conducted multiple regression analyses (with and without bootstrapping) separately for each of the five cognitive assessments. The results are displayed in Table 3. All models revealed to be significant (MMSE: *F*(3, 4301) = 160, *p* < 0.001; *R*^2^_adj_ = 0.10; FAS: *F*(3, 2745) = 115.6, *p* < 0.001; *R*^2^_adj_ = 0.11; ROCFT copy: *F*(3, 2366) = 106.9, *p* < 0.001; *R*^2^_adj_ = 0.12; ROCFT delayed recall: *F*(3, 2523) = 191.7, *p* < 0.001; *R*^2^_adj_ = 0.19; TMT-A: *F*(3, 3344) = 294.6, *p* < 0.001; *R*^2^_adj_ = 0.21; TMT-B: *F*(3, 2505) = 355.9, *p* < 0.001; *R*^2^_adj_ = 0.30). 

As can be seen in Table 3 for both types of regression analyses, sociodemographic effects significantly predicted test scores in all cognitive assessments. Whereas the MMSE, TMT-A, TMT-B and ROCFT copy were affected by age and educational level, the FAS and ROCFT delayed recall were influenced by age, educational level as well as gender. Throughout all analyses made, a lower age and higher educational level predicted better test scores (MMSE, FAS, ROCFT) or processing times (TMT-A and -B). Additionally, independent of age and educational level, female patients scored significantly higher than male patients on the FAS (i.e., they named more words) but lower on the ROCFT delayed recall. For a summary of sociodemographic effects found and corresponding effect sizes, see Table 4.

**Figure 2:**
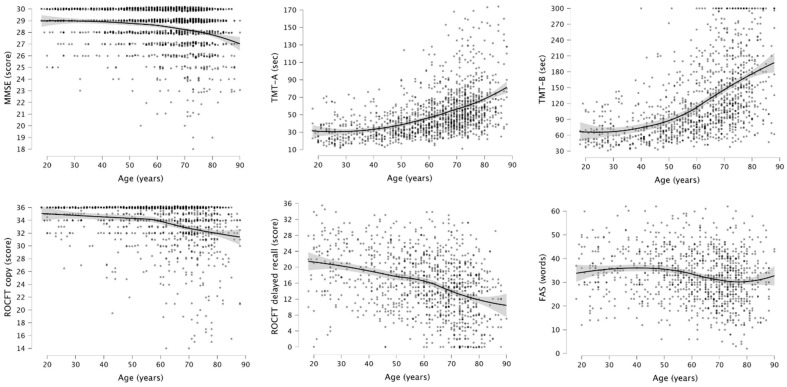
Bivariate LOESS plot (with 95% confidence intervals) of cognitive performances of the neuropsychological tests across the lifespan (cross-sectional and independent of education or gender).

The relationship between neuropsychological test scores and age were graphically illustrated with LOESS scatterplots (Figure 2). For the MMSE, it can be seen that test scores remain relatively steady until the age of 60, following by a decline in performance until the age of 90. Times of completion for TMT-A and -B appear to increase slowly until the age of 40, followed by a stronger increase until the age of 90. For TMT-B, the displayed line seems to slightly flatten out in the last decade of life. The curves for the ROCFT copy and delayed recall are quite similar, with a somewhat stronger decline in scores for the ROCFT delayed recall. Furthermore, both parts of the ROCFT show a salient point at the age of 60. Lastly, in the scatterplot of the generated words during the FAS, it can be seen that the subjects’ production of words slightly increases until the age of 40, followed by a decline between 50 and 60 years of age.

### 3.3. Percentile Rank Generation and z-Score Transformation

Based on the results of the multiple regression analyses, new clinical normative data for the MMSE, ROCFT copy, TMT-A and TMT-B should be offered separately for distinct age groups and educational levels, whereas normative data for the FAS and the ROCFT delayed recall should be divided into age groups, males vs. females, and different educational levels. Therefore, several groups with sufficient sample sizes (*n* ≥ 10) were created for subjects with normal cognition based on their DemTect scores. The obtained groups, their sample sizes as well as the means and standard deviations of the test scores for each group can be seen in Table 5 and Table 6. 

As a next step, we calculated percentile ranks for the test scores of the cognitive assessments and transformed them into equivalent *z*-scores [54] (Table 7 shows an example for the MMSE and individuals with 12 and more years of education; see Supplementary Tables S1–S16 for the complete clinical normative data). Scores that were one or more standard deviations below the mean within the corresponding sample (i.e., percentile rank of 16 and below) were interpreted as cognitive impairment [56]. 

**Figure 3:**
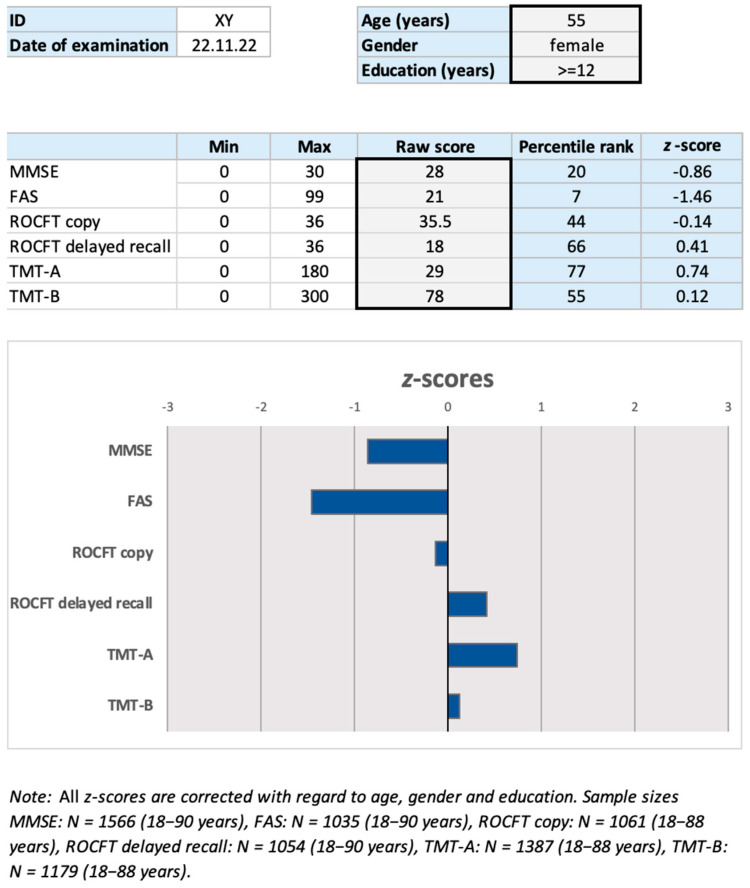
User interface of the scoring worksheet (with example data) that provides corrected *z*-scores and percentile ranks for the neuropsychological tests. First, the user is required to insert sociodemographic details: the age of the participant (an integer) as well as the gender (male, female) and educational level (<12, ≥12 years) from a drop-down menu. Second, the raw score(s) from the conducted test(s) needs to be filled in. The raw score cells of tests not conducted should be left empty. With an illustrative figure, the *z*-scores for each of the conducted tests are shown by means of a colored bar.

In order to ease the lookup and use of the updated clinical norms from the present study, we provide a user-friendly Microsoft Excel file for test score interpretation (see Figure 3). The file requires the user to shortly insert the patient’s sociodemographics (age, gender, educational level) and the corresponding test score in one or more of the five cognitive assessments. It then returns and graphically illustrates the patient’s performance by showing his/her percentile rank and *z*-score. The file can be downloaded for free at https://neurologie.uk-koeln.de/forschung/ag-neuropsychologie/ or https://figshare.com/ (accessed on 4 July 2023).

## 4. Discussion 

The present study retrospectively investigated sociodemographic effects on test scores of five commonly used assessments—MMSE, FAS, ROCFT, TMT-A and TMT-B—for assessing the presence of cognitive impairments in a sample of 4968 patients. Furthermore, updated age-, gender- and education-corrected clinical normative data (percentile ranks and *z*-scores) from a control group (DemTect ≥ 13/18 points; *n* = 1603) were provided. 

The test scores of all cognitive assessments investigated were significantly predicted by sociodemographics such as age, gender and educational level. MMSE scores, copied drawings on the ROCFT as well as TMT-A and -B times for completion were affected by age and education (but not gender), whereas produced words on the FAS and remembered drawings (delayed recall) of the ROCFT were influenced by age, gender and education. In general, it has been frequently shown that older patients and those with lower educational level tend to score lower on neuropsychological tests than younger and more educated people [5,6,7,8,9,14,15,16,25,26]

Brain health and age-related cognitive decline have been studied extensively. The course and extent of cognitive deterioration are influenced by various factors, such as lifestyle, education, genetics and cognitive engagement [57,58]. A recent study by Lin et al. [58], using data of 17030 participants from the UK Biobank, identified several significant features that were meaningfully associated with brain and cognitive maintenance. Particularly, smoking behavior and intensity, chronic and heavy alcohol consumption, a sedentary lifestyle and medical variables such as diabetes and hypertension had a negative impact on brain health. 

Generally, it is assumed that “crystallized” abilities such as intellectual abilities and vocabulary appear to be more resistant to age effects than “fluid” abilities such as attention and executive functions [59]. Overall, age effects on cognition can at least partially be explained by a general cognitive slowing [60] and by a deterioration of subcomponents of executive functions [61]. Memory functions appear to be most affected, especially those related to episodic memory [59]. Neuroanatomically, it seems that cognitive decline depends on changes in gray [62,63] and also white matter [64,65]. On the biochemical level, a decrease in the neurotransmitter dopamine appears to be associated with lower scores in tasks requiring psychomotor speed [66]. 

Anstey and Christensen [5] stated that educational effects on cognitive test scores may be explained by the following four assumptions: (1) education level may represent a proxy for other effects such as socioeconomic status and nutrition, health behavior as well as exposure to occupational stress and hazards; (2) education may stimulate dendritic growth, increase the number of synapses and may enhance vascularization of the brain, leading to neurochemical structural alterations in the brain (thus, increasing the brain’s reserves); (3) education may preserve learning (and not the rate of biological decline); and (4) educational effects may be present so strongly because they are ongoing throughout a whole life, instead of being limited to a (short) critical period. 

Overall, influences of gender on neuropsychological test scores have not been observed as clear-cut as the influence of age and educational level. We found that female patients scored significantly higher than male patients on the FAS (i.e., they named more words) but lower on the ROCFT delayed recall. Previous studies did also find the same influence of gender on FAS scores [17,18] and the delayed recall of the ROCFT [25,27,28], although others are not in line with our findings [19,20,26]. 

Generally, it has been frequently found that women are more likely than men to suffer from cognitive impairment [57,67,68,69]. Furthermore, an examination of 34 439 elderly individuals with normal cognition revealed that women might have greater cognitive reserve but faster cognitive decline than men [68]. Based on other previous research, it could be hypothesized that the women in our sample may have exhibited higher verbal intelligence, since this trait has been frequently shown to be associated with better FAS performance [17,70,71]. According to Bolla and colleagues [17], an explanation is that that people with higher verbal intelligence probably employ more effective recall strategies, and have a broader knowledge of vocabulary, organizational skills and semantic linguistic facility. Thus, this could have also explained the gender influence on the FAS scores in our study. Concerning the ROCFT, men could have benefitted from their superior ability in mental imagery as well as spatial perception, visualization and rotation [59,72,73]. This could also be the reason why men may generally be able to perform better than women on episodic memory tasks requiring such processing [74]. 

A few limitations of the present study warrant consideration. First, in order to generate updated normative data, we only used data of patients without dementia according to the DemTect (13 or more out of 18 points). Since our sample still includes patients with neurological diseases recruited at the University Hospital of Cologne, it does not represent a usual “healthy” control group. As a result, some participants may still fall within the range of mild cognitive impairment or even early dementia based on their MMSE scores. Consequently, our clinical norms are probably not as “strict” as standard norms (based on data including only individuals without any medical/neurological condition). As an advantage of our approach, patients who come for neuropsychological diagnostics and who score below the percentile ranks provided in this study will most likely indeed show cognitive decline. Thus, false-positive diagnoses will be reduced. In order to decrease the risk of false-negative diagnoses, patients who show borderline performance (e.g., a percentile rank of 20) will, however, need to go through additional testing (level II diagnostics) to validate the finding [75,76]. However, this approach is not uncommon in clinical neuropsychology and screening instruments are not used solely during the assessment of cognitive performance anyway. Their results should rather serve as an impetus for further examination [76]. The “optimal” accuracy of test score interpretation will be probably reached when all the tests discussed in the study are used simultaneously.

Second, we used data from a relatively long period of time (11 years), and it could be argued that the test scores may be influenced by certain events, such as, for instance, new medical treatments, changes in clinic procedures or health issues. However, we assume such influences on our sample to be minimal, since the referral procedure, the reasons for neuropsychological examination (e.g., subjective experience of cognitive deficits), as well as inclusion and exclusion criteria for the assessment did not change over time. 

Third, due to our clinical sample, the recruited subjects are not a perfect reflection of the general population in Germany. For instance, our sample appeared to be somewhat older (*M* = 65 years of age) than what is expected of the mean population in the community. However, we think that the large sample size overall allows for a well-suited representation of subjects who report subjective cognitive complaints. 

Fourth, we did not find a perfectly linear decline in neuropsychological test scores with increasing age, probably specifically due to several small-sized age groups (thereby increasing sampling error), cohort differences associated with the cross-sectional nature of the study, the nonnormally distributed test scores, or, more generally, because of the inherently not perfectly linear trajectory of these phenomena over the entire adult lifespan. This had an influence on some of the generated *z*-scores and percentile ranks (see the Supplementary Tables S1–S16). For instance, for the MMSE scores in subjects with 12 or more years of education, those between 60 and 69 years of age received a percentile rank of 5 for a score of 25, whereas those in the following age group (70 to 79 years) received a percentile rank of 4 for the same MMSE score. This is counterintuitive because—in case of a negative age effect—we would expect older persons receiving a higher percentile rank for the same score than younger persons (i.e., because they have an “age bonus”). Because age-related trends are clearly present, as can be seen in the nonparametric LOESS-smoothed scatterplots and because the effect is small and only concerns some of the adjacent age groups, we do not expect the provided norms to be invalidated. Furthermore, as outlined above, in the case of borderline cognitive performance, further neuropsychological testing is required anyhow.

In conclusion, we found influences of patients’ age, gender, and educational level on common cognitive assessments, although these factors are frequently not or only partially considered for test score interpretation. This study offers corrected and updated clinical norms by providing percentile ranks and *z*-scores for five widely used cognitive assessments. They may supplement standard normative data that is already available.

By providing a user-friendly Microsoft Excel file that assists test score interpretation, clinical neuropsychologists are enabled to determine the degree to which scores on either of the five instruments reflect impaired performance for male and female subjects (of varying ages and educational levels) with cognitive complaints but without dementia.

## Figures and Tables

**Table 1:** Sociodemographics and neuropsychological test scores.

	Entire Sample (*n* = 4968)	DemTect ≥ 13 (*n* = 1603)	DemTect < 13 (*n* = 2518)
Age (Years) [range]	65.3 ± 15.6 [18–90]	61.2 ± 16.3 [18–90]	68 ± 14.6 [18–90]
Gender (% female)	43.6	47.1	42.9
Education ≥ 12 years	36.8	49.5	29.6
Education < 12 years	63.2	50.5	70.4
Cognition			
DemTect [range]	10.8 ± 4.5 [0–18]	-	-
MMSE [range]	26.4 ± 3.3 [17–30]	28.4 ± 1.8 [18–30]	25.2 ± 3.4 [17–30]
FAS [range]	24.9 ± 12.7 [0–62]	32.7 ± 10.8 [2–62]	19.4 ± 11 [0–61]
ROCFT copy [range]	31.6 ± 5.4 [13.5–36]	33.4 ± 3.8 [14–36]	29.8 ± 6.1 [13.5–36]
ROCFT delayed recall ^+^ [range]	11.8 ± 8.3 [0–35.5]	15.7 ± 7.7 [0–35.5]	8.1 ± 7.3 [0–32]
TMT-A [range] ^++^	64.4 ± 35.2 [11–175]	50 ± 25.9 [11–174]	76.6 ± 36.9 [11–175]
TMT-B [range] ^+++^	154.3 ± 80.6 [31–300]	123.7 ± 68.9 [31–300]	190 ± 78.1 [32–300]

^+^ after 30 min, ^++^ stop after 3 min, ^+++^ stop after 5 min. Values as means ± standard deviation for continuous variables and % for categorical variables; DemTect = Dementia Detection Test; MMSE = Mini-Mental State Examination; FAS = F-A-S Test; ROCFT = Rey–Osterrieth Complex Figure Test; TMT = Trail Making Test.

**Table 2:** Spearman’s rank correlation coefficients for the neuropsychological test scores.

Test	MMSE	FAS	ROCFT Copy	RCOFT Delayed Recall ^+^	TMT-A	TMT-B
MMSE	-					
FAS	0.48 *	-				
ROCFT copy	0.37 *	0.37 *	-			
ROCFT delayed recall ^+^	0.54 *	0.41 *	0.53 *	-		
TMT-A	−0.45 *	−0.49 *	−0.46 *	−0.48 *	-	
TMT-B	−0.47 *	−0.50 *	−0.51 *	−0.52 *	0.76 *	-

^+^ after 30 min, * *p* < 0.001; MMSE = Mini-Mental State Examination; FAS = F-A-S Test; ROCFT = Rey–Osterrieth Complex Figure Test; TMT = Trail Making Test.

**Table 3:** Multiple linear regression models (with and without bootstrapping) examining the effects of sociodemographics on neuropsychological test results.

Variables	Without Bootstrapping					With Bootstrapping (5000 Iterations)				
	b	SE	β	*t*	*p*				95% CI	
						b	SE	*p*	Lower	Upper
Dependent variable: MMSE (*n* = 4302)										
Age	−0.06	0.003	−0.26	−17.98	<0.001 **	−0.06	0.003	<0.001 **	−0.06	−0.05
Gender ^a^	−0.08	0.10		−0.80	0.42	−0.08	0.10	0.44	−0.27	0.11
Education ^b^	−1.02	0.10		−10.32	<0.001 **	−1.02	0.10	<0.001 **	−1.21	−0.83
Dependent variable: FAS (*n* = 2746)										
Age	−0.17	0.02	−0.22	−11.93	<0.001 **	−0.17	0.01	<0.001 **	−0.20	−0.15
Gender ^a^	1.58	0.47		3.39	<0.001 **	1.58	0.47	0.003 *	0.65	2.48
Education ^b^	−5.98	0.48		−12.56	<0.001 **	−5.99	0.49	<0.001 **	−6.92	−5.0
Dependent variable: ROCF copy (*n* = 2367)										
Age	−0.09	0.01	−0.29	−14.70	<0.001 **	−0.09	0.006	<0.001 **	−0.11	−0.08
Gender ^a^	−0.09	0.21		−0.42	0.43	−0.09	0.21	0.66	−0.50	0.31
Education ^b^	−1.71	0.21		−7.98	<0.001 **	−1.72	0.21	<0.001 **	−2.10	−1.29
Dependent variable: ROCF delayed recall ^+^ (*n* = 2524)										
Age	−0.19	0.01	−0.37	−20.27	<0.001 **	−0.19	0.009	<0.001 **	−0.20	−0.17
Gender ^a^	−1.56	0.30		−5.15	<0.001 **	−1.56	0.30	<0.001 **	−2.15	−0.98
Education ^b^	−2.56	0.31		−8.26	<0.001 **	−2.56	0.31	<0.001 **	−3.15	−1.95
Dependent variable: TMT-A (*n* = 3345)										
Age	0.94	0.03	0.43	27.61	<0.001 **	0.94	0.03	<0.001 **	0.88	1.00
Gender ^a^	0.20	1.10		0.18	0.86	0.20	1.11	0.86	−1.99	2.36
Education ^b^	8.47	1.12		7.58	<0.001 **	8.46	1.10	<0.001 **	6.34	10.66
Dependent variable: TMT-B (*n* = 2506)										
Age	2.42	0.08	0.50	29.26	<0.001 **	2.42	0.08	<0.001 **	2.28	2.58
Gender ^a^	−3.17	2.74		−1.16	0.25	−3.18	2.74	0.24	−8.66	2.21
Education ^b^	27.86	2.76		10.09	<0.001 **	27.96	2.72	<0.001 **	22.37	33.16

^a^ female, ^b^ <12 years, ^+^ after 30 min, ** *p* < 0.001, * *p* < 0.05; CI = Confidence Interval; MMSE = Mini-Mental State Examination; FAS = F-A-S Test; ROCFT = Rey–Osterrieth Complex Figure Test; TMT = Trail Making Test.

**Table 4:** Summary of sociodemographic effects on neuropsychological test scores.

Test	Sociodemographic Effect			$\mathrm{Effect}\;\mathrm{Size}\;(f^{2})$	Interpretation According to Cohen [55]
	Age	Gender	Education		
MMSE	−	0	+	0.11	Small
FAS	−	♀↑	+	0.13	Small
ROCFT copy	−	0	+	0.14	Small
ROCFT delayed recall	−	♂↑	+	0.23	Medium
TMT-A	−	0	+	0.26	Medium
TMT-B	−	0	+	0.43	Large

– negative influence, + positive influence, 0 no influence, ♀↑ females better than males, ♂↑ males better than females; MMSE = Mini-Mental State Examination; FAS = F-A-S Test; ROCFT = Rey–Osterrieth Complex Figure Test; TMT = Trail Making Test.

**Table 5:** Neuropsychological test scores for low and high educational level, divided into age groups.

Test	Education (Years)	Age (Years)					
		18–29	30–49	50–59	60–69	70–79	≥80
MMSE	≥12	29.2 ± 1.1 (*n* = 65)	29.2 ± 1.2 (*n* = 146)	29 ± 1.2 (*n* = 127)	28.5 ± 1.8 (*n* = 180)	28.4 ± 1.7 (*n* = 195)	27.7 ± 2 (*n* = 62)
	<12	28.7 ± 1.5 (*n* = 40)	28.4 ± 1.5 (*n* = 100)	28.5 ± 1.7 (*n* = 109)	28.5 ± 1.5 (*n* = 205)	27.7 ± 2.3 (*n* = 268)	27.6 ± 2.2 (*n* = 69)
TMT-A	≥12	29.9 ± 13.5 (*n* = 64)	32.1 ± 12.2 (*n* =133)	40.7 ± 16.4 (*n* = 118)	47.6 ± 21 (*n* = 166)	59.4 ± 26.1 (*n* = 171)	68.6 ± 36.7 (*n* = 53)
	<12	30.5 ± 13.6 (*n* = 38)	36.4 ± 12.9 (*n* = 87)	45 ± 20.2 (*n* = 91)	53.1 ± 22.8 (*n* = 180)	63.2 ± 27.7 (*n* = 226)	74 ± 32.9 (*n* = 60)
TMT-B	≥12	61.8 ± 22.5 (*n* = 59)	72.5 ± 38.3 (*n* = 123)	93.4 ± 44.2 (*n* = 103)	120.3 ± 62.13 (*n* = 142)	146.7 ± 63.6 (*n* = 140)	170.2 ± 84.3 (*n* = 44)
	<12	74.7 ± 25.1 (*n* = 29)	84.8 ± 37.8 (*n* = 73)	102.2 ± 43.7 (*n* = 82)	140.1 ± 68 (*n* = 146)	167.2 ± 68.4 (*n* = 186)	198.4 ± 70.1 (*n* = 52)

Values as means ± standard deviation; MMSE = Mini-Mental State Examination; TMT = Trail Making Test.

**Table 6:** Neuropsychological test scores of male and female patients for low and high educational level, divided into age groups.

Test	Gender	Education (Years)	Age (Years)				
			18–29	30–49	50–64	65–74	≥75
FAS	Male	≥12	35.2 ± 10.8 (*n* = 19)	37.5 ± 8.5 (*n* = 60)	37.5 ± 9.6 (*n* = 77)	31.2 ± 11.1 (*n* = 93)	32.7 ± 10.9 (*n* = 60)
		<12	31.5 ± 9.7 (*n* = 17)	30.8 ± 10.5 (*n* = 31)	32.8 ± 10.5 (*n* = 66)	29.1 ± 8.6 (*n* = 82)	27.3 ± 10.3 (*n* = 64)
	Female	≥12	35.2 ± 11.6 (*n* = 26)	36.3 ± 11.5 (*n* = 42)	35 ± 10.4 (*n* = 65)	36.4 ± 9 (*n* = 43)	35.2 ± 11 (*n* = 35)
		<12	34.8 ± 8.2 (*n* = 10)	36.2 ± 11.8 (*n* = 34)	32 ± 9.9 (*n* = 63)	29.4 ± 10.6 (*n* = 75)	28.5 ± 11.6 (*n* = 73)
ROCFT copy	-	≥12	34.9 ± 1.6 (*n* = 56)	34.8 ± 2.3 (*n* = 110)	34.3 ± 2.8 (*n* = 153)	33.4 ± 3.8 (*n* = 150)	32.6 ± 3.9 (*n* = 82)
		<12	34.7 ± 2.1 (*n* = 32)	33.6 ± 3.6 (*n* = 70)	33.6 ± 3.6 (*n* = 124)	32.5 ± 4.3 (*n* = 159)	31.6 ± 5.1 (*n* = 125)
ROCFT delayed recall ^+^	Male	≥12	22.7 ± 6.9 (*n* = 26)	19.7 ± 7.2 (*n* = 63)	19.2 ± 6.3 (*n* = 80)	15.6 ± 7.8 (*n* = 96)	13.5 ± 7.2 (*n* = 51)
		<12	18.9 ± 5.8 (*n* = 17)	19.9 ± 8.2 (*n* = 36)	16.7 ± 7.5 (*n* = 66)	14.6 ± 7.2 (*n* = 79)	12.7 ± 7.7 (*n* = 64)
	Female	≥12	21.6 ± 9.1 (*n* = 28)	18.5 ± 6.9 (*n* = 46)	15.8 ± 5.9 (*n* = 72)	12 ± 5.7 (*n* = 51)	12.6 ± 6.5 (*n* = 32)
		<12	18 ± 8.5 (*n* = 12)	16.5 ± 5.5 (*n* = 33)	15 ± 6.7 (*n* = 59)	12.7 ± 7.3 (*n* = 78)	9.9 ± 6.4 (*n* = 65)

^+^ after 30 min. Values as means ± standard deviation; FAS = F-A-S Test; ROCFT = Rey–Osterrieth Complex Figure Test.

**Table 7:** Percentile ranks and *z*-scores per age group for the Mini-Mental State Examination (≥12 years of education).

Test Score	Age (Years)											
	18–29 (*n* = 65)		30–49 (*n* = 146)		50–59 (*n* = 127)		60–69 (*n* = 180)		70–79 (*n* = 195)		≥80 (*n* = 62)	
	PR	*z*-Score	PR	*z*-Score	PR	*z*-Score	PR	*z*-Score	PR	*z*-Score	PR	*z*-Score
≤20	0	−3	0	−3	0	−3	0	−3	0	−3	0	−3
21	0	−3	0	−3	0	−3	1	−2.54	1	−2.57	0	−3
22	0	−3	0	−3	0	−3	1	−2.2	1	−2.32	1	−2.41
23	0	−3	0	−3	0	−3	2	−2.01	1	−2.24	4	−1.75
24	0	−3	0	−2.71	0	−2.66	3	−1.84	2	−2	8	−1.41
25	1	−2.43	1	−2.47	1	−2.42	5	−1.62	4	−1.71	11	−1.22
26	3	−1.87	3	−1.92	3	−1.86	8	−1.39	10	−1.3	18	−0.93
27	5	−1.61	7	−1.52	8	−1.39	15	−1.03	18	−0.9	29	−0.56
28	11	−1.24	14	−1.07	20	−0.86	30	−0.54	33	−0.46	46	−0.11
29	32	−0.46	33	−0.44	43	−0.19	53	0.07	57	0.16	69	0.48
30	75	0.66	73	0.61	78	0.78	84	0.97	86	1.06	90	1.3

White cells correspond to cognitive impairment. Cells highlighted in gray represent normal cognition; PR = percentile rank.

## Data Availability

The data presented in this study are available on request from the corresponding authors. The data are not publicly available due to data privacy.

## Supplementary Materials

The following supporting information can be downloaded at: https://www.mdpi.com/article/10.3390/jcm12165170/s1. Supplementary Tables S1–S16.
