# Supplementary material for: The Influence of Age, Gender and Education on Neuropsychological Test Scores: Updated Clinical Norms for Five Widely Used Cognitive Assessments
Source: J Clin Med. 2023 Aug 8;12(16):5170. doi: 10.3390/jcm12165170 (PMC10455991; doi:10.3390/jcm12165170)
Supplement: Supplementary file 1 [file jcm-12-05170-s001.zip › jcm-2517312-supplementary.pdf]

Clinical Normative Data for the Mini-Mental State Examination, F-A-S Test, Rey-Osterrieth Complex Figure Test (copy and delayed recall), and Trail Making Test, Part A and B

Mini-Mental State Examination

Table S1. Percentile ranks and *z*-scores per age group for the Mini-Mental State Examination (< 12 years of education).

Test score	Age (years)											
	18-29 (*n* = 40)		30-49 (*n* = 100)		50-59 (*n* = 109)		60-69 (*n* = 205)		70-79 (*n* = 268)		≥ 80 (*n* = 69)	
	PR	*z*-score	PR	*z*-score	PR	*z*-score	PR	*z*-score	PR	*z*-score	PR	*z*-score
≤ 17	0	-3	0	-3	0	-3	0	-3	0	-3	0	-3
18	0	-3	0	-3	0	-3	0	-3	0	-2.89	0	-3
19	0	-3	0	-3	0	-3	0	-3	1	-2.54	1	-2.45
20	0	-3	0	-3	0	-2.61	0	-3	1	-2.36	1	-2.19
21	0	-3	0	-3	1	-2.36	0	-3	2	-2.13	1	-2.19
22	0	-3	0	-3	1	-2.21	0	-3	3	-1.92	1	-2.19
23	0	-3	1	-2.575	2	-2	0	-2.82	5	-1.66	3	-1.9
24	0	-3	2	-2.06	3	-1.92	1	-2.34	8	-1.4	6	-1.58
25	4	-1.78	5	-1.7	4	-1.79	3	-1.9	12	-1.16	13	-1.13
26	9	-1.36	9	-1.38	7	-1.49	8	-1.39	18	-0.91	23	-0.74
27	15	-1.04	15	-1.04	15	-1.04	17	-0.95	29	-0.54	33	-0.44

Table S1. Percentile ranks and *z*-scores per age group for the Mini-Mental State Examination (< 12 years of education).

Test score	Age (years)											
	18-29 (*n* = 40)		30-49 (*n* = 100)		50-59 (*n* = 109)		60-69 (*n* = 205)		70-79 (*n* = 268)		≥ 80 (*n* = 69)	
	PR	*z*-score	PR	*z*-score	PR	*z*-score	PR	*z*-score	PR	*z*-score	PR	*z*-score
28	24	-0.72	32	-0.49	29	-0.55	32	-0.47	44	-0.16	49	-0.04
29	48	-0.07	58	0.2	54	0.09	56	0.15	65	0.37	71	0.55
30	84	0.98	86	1.08	85	1.03	85	1.05	89	1.23	92	1.4

White cells correspond to cognitive impairment. cells highlighted in gray represent normal cognition; **PR** Percentile rank.

Table S2. Percentile ranks and *z*-scores per age group for the Mini-Mental State Examination (≥ 12 years of education).

Test score	Age (years)											
	18-29 (*n* = 65)		30-49 (*n* = 146)		50-59 (*n* = 127)		60-69 (*n* = 180)		70-79 (*n* = 195)		≥ 80 (*n* = 62)	
	PR	*z*-score	PR	*z*-score	PR	*z*-score	PR	*z*-score	PR	*z*-score	PR	*z*-score
≤ 20	0	-3	0	-3	0	-3	0	-3	0	-3	0	-3
21	0	-3	0	-3	0	-3	1	-2.54	1	-2.57	0	-3
22	0	-3	0	-3	0	-3	1	-2.2	1	-2.32	1	-2.41
23	0	-3	0	-3	0	-3	2	-2.01	1	-2.24	4	-1.75
24	0	-3	0	-2.71	0	-2.66	3	-1.84	2	-2	8	-1.41
25	1	-2.43	1	-2.47	1	-2.42	5	-1.62	4	-1.71	11	-1.22
26	3	-1.87	3	-1.92	3	-1.86	8	-1.39	10	-1.3	18	-0.93
27	5	-1.61	7	-1.52	8	-1.39	15	-1.03	18	-0.9	29	-0.56
28	11	-1.24	14	-1.07	20	-0.86	30	-0.54	33	-0.46	46	-0.11
29	32	-0.46	33	-0.44	43	-0.19	53	0.07	57	0.16	69	0.48
30	75	0.66	73	0.61	78	0.78	84	0.97	86	1.06	90	1.3

White cells correspond to cognitive impairment. cells highlighted in gray represent normal cognition; **PR** Percentile rank.

F-A-S Test

Males

Table S3. Percentile ranks and *z*-scores per age group for the F-A-S Test (males, < 12 years of education).

Test score	Age (years)									
	18-29 (*n* = 17)		30-49 (*n* = 31)		50-64 (*n* = 66)		65-74 (*n* = 82)		≥ 75 (*n* = 64)	
	PR	*z*-score	PR	*z*-score	PR	*z*-score	PR	*z*-score	PR	*z*-score
0	0	-3	0	-3	0	-3	0	-3	0	-3
1	0	-3	0	-3	0	-3	0	-3	0	-3
2	0	-3	0	-3	0	-3	0	-3	1	-2.42
3	0	-3	0	-3	0	-3	0	-3	2	-2.16
4	0	-3	0	-3	0	-3	0	-3	2	-1.99
5	0	-3	0	-3	0	-3	0	-3	4	-1.77
6	0	-3	2	-2.15	0	-3	0	-3	5	-1.68
7	0	-3	3	-1.85	0	-3	0	-3	5	-1.68
8	0	-3	3	-1.85	0	-3	0	-3	5	-1.68
9	0	-3	3	-1.85	0	-3	0	-3	5	-1.68
10	0	-3	3	-1.85	0	-3	1	-2.51	5	-1.68
11	0	-3	3	-1.85	0	-3	1	-2.25	5	-1.68
12	3	-1.89	3	-1.85	1	-2.43	1	-2.25	5	-1.61
13	6	-1.57	3	-1.85	2	-2.17	2	-2.09	7	-1.48
14	6	-1.57	5	-1.67	2	-2.01	3	-1.88	9	-1.37

Table S3. Percentile ranks and *z*-scores per age group for the F-A-S Test (males, < 12 years of education).

Test score	Age (years)									
	18-29 (*n* = 17)		30-49 (*n* = 31)		50-64 (*n* = 66)		65-74 (*n* = 82)		≥ 75 (*n* = 64)	
	PR	*z*-score	PR	*z*-score	PR	*z*-score	PR	*z*-score	PR	*z*-score
15	6	-1.57	10	-1.3	4	-1.78	4	-1.72	10	-1.28
16	6	-1.57	13	-1.14	5	-1.69	5	-1.6	13	-1.12
17	6	-1.57	13	-1.14	5	-1.62	7	-1.5	18	-0.92
18	6	-1.57	13	-1.14	7	-1.49	10	-1.27	21	-0.81
19	12	-1.19	13	-1.14	8	-1.39	15	-1.03	23	-0.73
20	18	-0.93	15	-1.06	11	-1.25	18	-0.93	25	-0.68
21	18	-0.93	19	-0.87	12	-1.17	20	-0.86	27	-0.63
22	18	-0.93	24	-0.71	13	-1.14	21	-0.82	29	-0.56
23	21	-0.83	27	-0.61	17	-0.94	24	-0.72	33	-0.45
24	24	-0.73	31	-0.51	22	-0.78	29	-0.55	38	-0.32
25	24	-0.73	32	-0.47	23	-0.73	32	-0.46	41	-0.24
26	24	-0.73	34	-0.42	26	-0.66	37	-0.33	42	-0.2
27	26	-0.63	35	-0.38	30	-0.52	44	-0.16	43	-0.18
28	35	-0.38	35	-0.38	35	-0.39	48	-0.05	48	-0.06
29	44	-0.15	35	-0.38	40	-0.25	51	0.01	54	0.09
30	47	-0.08	39	-0.29	45	-0.14	53	0.07	59	0.21
31	47	-0.08	45	-0.13	47	-0.08	57	0.16	63	0.33
32	50	0	48	-0.05	52	0.03	61	0.27	67	0.44
33	53	0.07	50	0	58	0.19	67	0.44	69	0.48

Table S3. Percentile ranks and *z*-scores per age group for the F-A-S Test (males, < 12 years of education).

Test score	Age (years)									
	18-29 (*n* = 17)		30-49 (*n* = 31)		50-64 (*n* = 66)		65-74 (*n* = 82)		≥ 75 (*n* = 64)	
	PR	*z*-score	PR	*z*-score	PR	*z*-score	PR	*z*-score	PR	*z*-score
34	56	0.14	52	0.04	61	0.28	73	0.61	70	0.53
35	59	0.22	55	0.12	64	0.34	76	0.71	75	0.67
36	62	0.29	60	0.24	67	0.45	79	0.79	82	0.91
37	65	0.37	68	0.46	70	0.51	81	0.88	87	1.11
38	71	0.54	76	0.7	70	0.53	82	0.92	88	1.18
39	76	0.72	77	0.75	72	0.58	85	1.02	90	1.27
40	82	0.92	79	0.8	73	0.6	88	1.16	91	1.36
41	88	1.18	81	0.86	73	0.62	90	1.29	93	1.47
42	88	1.18	84	0.98	76	0.69	92	1.41	94	1.53
43	91	1.35	89	1.21	78	0.77	93	1.49	95	1.6
44	94	1.56	90	1.3	80	0.82	95	1.59	96	1.76
45	94	1.56	95	1.66	82	0.9	96	1.72	97	1.86
46	94	1.56	100	3	86	1.06	98	1.97	98	1.98
47	94	1.56	100	3	91	1.33	99	2.5	98	2.15
48	94	1.56	100	3	94	1.55	100	3	98	2.15
49	97	1.89	100	3	95	1.61	100	3	98	2.15
50	100	3	100	3	95	1.69	100	3	98	2.15
> 50	100	3	100	3	98	2	100	3	99	2.42

White cells correspond to cognitive impairment. cells highlighted in gray represent normal cognition; **PR** Percentile rank.

Table S4. Percentile ranks and *z*-scores per age group for the F-A-S Test (males, ≥ 12 years of education).

Test score	Age (years)									
	18-29 (*n* = 19)		30-49 (*n* = 60)		50-64 (*n* = 77)		65-74 (*n* = 93)		≥ 75 (*n* = 60)	
	PR	*z*-score	PR	*z*-score	PR	*z*-score	PR	*z*-score	PR	*z*-score
≤ 4	0	-3	0	-3	0	-3	0	-3	0	-3
5	0	-3	0	-3	0	-3	1	-2.55	0	-3
6	0	-3	0	-3	0	-3	1	-2.3	0	-3
7	0	-3	0	-3	0	-3	1	-2.3	0	-3
8	0	-3	0	-3	0	-3	2	-2.15	1	-2.4
9	0	-3	0	-3	0	-3	2	-2.03	2	-2.13
10	0	-3	0	-3	0	-3	2	-2.03	2	-2.13
11	0	-3	0	-3	0	-3	3	-1.93	3	-1.96
12	0	-3	0	-3	0	-3	4	-1.78	3	-1.84
13	0	-3	0	-3	0	-3	5	-1.67	4	-1.74
14	0	-3	0	-3	0	-3	5	-1.61	6	-1.57
15	3	-1.94	0	-3	0	-3	6	-1.52	8	-1.44
16	5	-1.62	0	-3	0	-3	8	-1.41	8	-1.39
17	8	-1.42	0	-3	0	-3	10	-1.3	8	-1.39
18	11	-1.26	0	-3	1	-2.23	11	-1.22	10	-1.29
19	11	-1.26	1	-2.4	3	-1.85	12	-1.19	13	-1.16
20	13	-1.12	3	-1.96	5	-1.69	15	-1.06	15	-1.04
21	16	-1.01	3	-1.84	6	-1.57	17	-0.95	18	-0.94
22	16	-1.01	3	-1.84	6	-1.52	19	-0.87	20	-0.85

Table S4. Percentile ranks and *z*-scores per age group for the F-A-S Test (males, ≥ 12 years of education).

Test score	Age (years)									
	18-29 (*n* = 19)		30-49 (*n* = 60)		50-64 (*n* = 77)		65-74 (*n* = 93)		≥ 75 (*n* = 60)	
	PR	*z*-score	PR	*z*-score	PR	*z*-score	PR	*z*-score	PR	*z*-score
23	16	-1.01	5	-1.65	7	-1.47	24	-0.72	22	-0.79
24	16	-1.01	7	-1.51	8	-1.38	27	-0.61	23	-0.76
25	16	-1.01	8	-1.44	10	-1.3	32	-0.48	23	-0.73
26	16	-1.01	10	-1.29	10	-1.26	36	-0.36	24	-0.71
27	16	-1.01	12	-1.2	12	-1.2	39	-0.29	26	-0.65
28	16	-1.01	12	-1.2	13	-1.13	41	-0.22	28	-0.58
29	16	-1.01	16	-1.01	16	-1.02	44	-0.15	33	-0.46
30	26	-0.64	21	-0.82	21	-0.82	47	-0.07	38	-0.3
31	37	-0.34	22	-0.79	25	-0.69	52	0.05	43	-0.17
32	37	-0.34	23	-0.73	28	-0.59	56	0.16	46	-0.11
33	39	-0.27	26	-0.65	34	-0.42	59	0.23	48	-0.07
34	47	-0.07	29	-0.55	40	-0.27	60	0.25	53	0.06
35	55	0.13	37	-0.35	44	-0.15	61	0.27	58	0.21
36	58	0.19	45	-0.13	48	-0.05	63	0.34	62	0.29
37	61	0.26	50	0	51	0.01	68	0.47	64	0.36
38	66	0.4	54	0.1	53	0.08	72	0.58	68	0.47
39	68	0.47	58	0.21	56	0.16	74	0.64	74	0.64
40	68	0.47	62	0.29	60	0.24	77	0.73	78	0.75
41	68	0.47	66	0.4	65	0.38	81	0.86	79	0.81

Table S4. Percentile ranks and *z*-scores per age group for the F-A-S Test (males, ≥ 12 years of education).

Test score	Age (years)									
	18-29 (*n* = 19)		30-49 (*n* = 60)		50-64 (*n* = 77)		65-74 (*n* = 93)		≥ 75 (*n* = 60)	
	PR	*z*-score	PR	*z*-score	PR	*z*-score	PR	*z*-score	PR	*z*-score
42	68	0.47	70	0.52	69	0.5	84	1.01	80	0.84
43	68	0.47	75	0.67	72	0.58	87	1.13	83	0.93
44	71	0.55	80	0.84	76	0.7	89	1.24	86	1.07
45	76	0.71	83	0.93	80	0.83	90	1.3	87	1.11
46	82	0.89	84	1	83	0.95	91	1.36	87	1.11
47	84	1	87	1.11	85	1.03	93	1.47	87	1.11
48	84	1	89	1.23	86	1.06	94	1.51	88	1.19
49	89	1.25	92	1.38	88	1.15	94	1.51	93	1.43
50	95	1.62	93	1.5	90	1.25	94	1.56	95	1.64
> 50	97	1.93	97	1.83	95	1.62	97	1.92	98	1.96

White cells correspond to cognitive impairment. cells highlighted in gray represent normal cognition; **PR** Percentile rank.

Females

Table S5. Percentile ranks and *z*-scores per age group for the F-A-S Test (females, < 12 years of education).

Test score	Age (years)									
	18-29 (*n* = 10)		30-49 (*n* =34)		50-64 (*n* = 63)		65-74 (*n* = 75)		≥ 75 (*n* = 73)	
	PR	*z*-score	PR	*z*-score	PR	*z*-score	PR	*z*-score	PR	*z*-score
≤ 2	0	-3	0	-3	0	-3	0	-3	0	-3
3	0	-3	0	-3	0	-3	0	-3	1	-2.47
4	0	-3	0	-3	0	-3	0	-3	2	-2.05
5	0	-3	0	-3	0	-3	0	-3	3	-1.92
6	0	-3	0	-3	1	-2.42	0	-3	3	-1.92
7	0	-3	0	-3	2	-2.15	0	-3	3	-1.92
8	0	-3	0	-3	2	-2.15	1	-2.48	3	-1.92
9	0	-3	0	-3	2	-2.15	1	-2.22	3	-1.92
10	0	-3	0	-3	2	-1.99	2	-2.06	3	-1.92
11	0	-3	0	-3	3	-1.86	3	-1.94	3	-1.92
12	0	-3	0	-3	4	-1.76	3	-1.84	5	-1.6
13	0	-3	1	-2.18	5	-1.67	5	-1.68	10	-1.31
14	0	-3	3	-1.89	5	-1.67	7	-1.51	13	-1.13
15	0	-3	3	-1.89	5	-1.67	8	-1.41	15	-1.04
16	0	-3	3	-1.89	5	-1.67	9	-1.37	15	-1.04
17	0	-3	3	-1.89	5	-1.67	11	-1.21	16	-0.98
18	0	-3	3	-1.89	6	-1.53	14	-1.09	19	-0.88
19	0	-3	3	-1.89	9	-1.36	16	-1	21	-0.8

Table S5. Percentile ranks and *z*-scores per age group for the F-A-S Test (females, < 12 years of education).

Test score	Age (years)									
	18-29 (*n* = 10)		30-49 (*n* =34)		50-64 (*n* = 63)		65-74 (*n* = 75)		≥ 75 (*n* = 73)	
	PR	*z*-score	PR	*z*-score	PR	*z*-score	PR	*z*-score	PR	*z*-score
20	0	-3	4	-1.71	10	-1.27	19	-0.89	22	-0.78
21	0	-3	7	-1.45	13	-1.15	22	-0.78	23	-0.73
22	0	-3	10	-1.27	16	-1	25	-0.67	27	-0.61
23	0	-3	13	-1.12	18	-0.91	29	-0.55	33	-0.45
24	5	-1.65	16	-0.99	20	-0.85	33	-0.44	38	-0.3
25	15	-1.04	19	-0.88	21	-0.82	37	-0.35	43	-0.18
26	20	-0.85	22	-0.77	22	-0.77	40	-0.26	47	-0.09
27	20	-0.85	26	-0.63	26	-0.64	42	-0.21	49	-0.02
28	20	-0.85	31	-0.5	29	-0.55	46	-0.11	51	0.03
29	25	-0.68	35	-0.38	33	-0.46	53	0.08	53	0.06
30	35	-0.39	38	-0.3	40	-0.27	58	0.2	55	0.13
31	40	-0.26	40	-0.27	47	-0.08	60	0.25	59	0.22
32	40	-0.26	41	-0.23	52	0.03	64	0.35	62	0.29
33	45	-0.13	41	-0.23	56	0.13	69	0.48	65	0.38
34	50	0	43	-0.19	58	0.2	71	0.56	69	0.5
35	50	0	44	-0.15	62	0.3	74	0.64	72	0.58
36	55	0.12	46	-0.12	66	0.4	77	0.72	74	0.64
37	65	0.38	49	-0.04	70	0.51	78	0.77	76	0.7
38	70	0.52	54	0.11	75	0.66	79	0.79	77	0.72

Table S5. Percentile ranks and *z*-scores per age group for the F-A-S Test (females, < 12 years of education).

Test score	Age (years)									
	18-29 (*n* = 10)		30-49 (*n* =34)		50-64 (*n* = 63)		65-74 (*n* = 75)		≥ 75 (*n* = 73)	
	PR	*z*-score	PR	*z*-score	PR	*z*-score	PR	*z*-score	PR	*z*-score
39	70	0.52	60	0.26	78	0.76	79	0.79	78	0.77
40	70	0.52	63	0.33	82	0.9	79	0.81	82	0.89
41	70	0.52	66	0.41	85	1.03	81	0.89	84	1
42	75	0.67	71	0.54	87	1.1	83	0.94	88	1.15
43	85	1.03	76	0.72	89	1.22	83	0.96	91	1.34
44	90	1.28	81	0.87	92	1.4	87	1.11	92	1.43
45	90	1.28	82	0.92	94	1.52	90	1.28	95	1.6
46	90	1.28	82	0.92	94	1.59	92	1.4	96	1.73
47	90	1.28	82	0.92	95	1.66	93	1.5	96	1.73
48	90	1.28	82	0.92	95	1.66	95	1.67	96	1.73
49	95	1.64	82	0.92	95	1.66	98	2.05	96	1.73
50	100	3	82	0.92	95	1.66	99	2.21	96	1.73
> 50	100	3	91	1.35	98	1.98	99	2.47	98	2.04

White cells correspond to cognitive impairment. cells highlighted in gray represent normal cognition; **PR** Percentile rank.

Table S6. Percentile ranks and *z*-scores per age group for the F-A-S Test (females, ≥ 12 years of education).

Test score	Age (years)									
	18-29 (*n* = 26)		30-49 (*n* = 42)		50-64 (*n* = 65)		65-74 (*n* = 43)		≥ 75 (*n* = 35)	
	PR	*z*-score	PR	*z*-score	PR	*z*-score	PR	*z*-score	PR	*z*-score
≤ 5	0	-3	0	-3	0	-3	0	-3	0	-3
6	0	-3	0	-3	1	-2.43	0	-3	0	-3
7	0	-3	0	-3	2	-2.16	0	-3	0	-3
8	0	-3	0	-3	2	-2.16	0	-3	0	-3
9	0	-3	0	-3	2	-2.16	0	-3	0	-3
10	0	-3	0	-3	2	-2.16	0	-3	0	-3
11	0	-3	0	-3	2	-2.16	0	-3	0	-3
12	0	-3	1	-2.26	2	-2.16	0	-3	0	-3
13	0	-3	2	-1.99	2	-2.16	0	-3	1	-2.19
14	4	-1.77	2	-1.99	2	-2	0	-3	3	-1.91
15	8	-1.43	2	-1.99	4	-1.77	1	-2.27	6	-1.58
16	8	-1.43	2	-1.99	5	-1.61	2	-1.99	9	-1.37
17	8	-1.43	2	-1.99	6	-1.55	2	-1.99	9	-1.37
18	10	-1.31	2	-1.99	6	-1.55	2	-1.99	9	-1.37
19	12	-1.2	2	-1.99	7	-1.49	2	-1.99	9	-1.37
20	12	-1.2	4	-1.81	8	-1.43	3	-1.82	9	-1.37
21	12	-1.2	7	-1.47	8	-1.43	5	-1.68	9	-1.37
22	12	-1.2	12	-1.18	8	-1.38	6	-1.58	9	-1.37
23	12	-1.2	15	-1.02	10	-1.29	7	-1.48	9	-1.37

Table S6. Percentile ranks and *z*-scores per age group for the F-A-S Test (females, ≥ 12 years of education).

Test score	Age (years)									
	18-29 (*n* = 26)		30-49 (*n* = 42)		50-64 (*n* = 65)		65-74 (*n* = 43)		≥ 75 (*n* = 35)	
	PR	*z*-score	PR	*z*-score	PR	*z*-score	PR	*z*-score	PR	*z*-score
24	13	-1.11	17	-0.97	13	-1.13	8	-1.4	10	-1.29
25	15	-1.03	19	-0.88	16	-0.99	12	-1.2	13	-1.14
26	15	-1.03	23	-0.76	18	-0.9	14	-1.09	14	-1.07
27	19	-0.87	24	-0.72	21	-0.82	15	-1.04	20	-0.85
28	23	-0.74	25	-0.68	22	-0.77	16	-0.99	26	-0.66
29	25	-0.68	27	-0.61	24	-0.72	17	-0.94	29	-0.57
30	35	-0.4	29	-0.57	25	-0.67	21	-0.81	31	-0.49
31	42	-0.2	32	-0.47	29	-0.55	26	-0.66	34	-0.41
32	42	-0.2	38	-0.31	35	-0.4	29	-0.56	40	-0.26
33	44	-0.15	40	-0.25	41	-0.24	36	-0.36	46	-0.11
34	48	-0.05	43	-0.18	48	-0.04	43	-0.18	51	0.03
35	52	0.04	49	-0.03	54	0.09	47	-0.09	56	0.14
36	58	0.19	55	0.11	58	0.19	50	0	60	0.25
37	65	0.39	58	0.21	61	0.27	52	0.05	63	0.32
38	69	0.5	61	0.27	62	0.31	55	0.11	64	0.36
39	69	0.5	62	0.3	65	0.39	57	0.17	66	0.4
40	69	0.5	62	0.3	70	0.52	63	0.32	66	0.4
41	69	0.5	63	0.33	73	0.61	69	0.48	66	0.4
42	69	0.5	65	0.39	76	0.71	73	0.62	69	0.48

Table S6. Percentile ranks and *z*-scores per age group for the F-A-S Test (females, ≥ 12 years of education).

Test score	Age (years)									
	18-29 (*n* = 26)		30-49 (*n* = 42)		50-64 (*n* = 65)		65-74 (*n* = 43)		≥ 75 (*n* = 35)	
	PR	*z*-score	PR	*z*-score	PR	*z*-score	PR	*z*-score	PR	*z*-score
43	71	0.55	68	0.46	78	0.78	79	0.8	74	0.65
44	73	0.61	73	0.6	80	0.84	81	0.89	77	0.74
45	77	0.73	77	0.75	82	0.92	85	1.03	80	0.84
46	81	0.86	80	0.83	85	1.05	90	1.25	84	1
47	81	0.86	82	0.92	89	1.23	92	1.39	89	1.2
48	81	0.86	85	1.01	91	1.32	93	1.47	91	1.36
49	85	1.02	87	1.12	92	1.37	94	1.57	91	1.36
50	90	1.3	88	1.18	93	1.48	95	1.68	91	1.36
> 50	96	1.76	94	1.55	97	1.86	98	1.99	96	1.71

White cells correspond to cognitive impairment. cells highlighted in gray represent normal cognition; **PR** Percentile rank.

Rey-Osterrieth Complex Figure Test – Copy Trial

Table S7. Percentile ranks and *z*-scores per age group for the Rey-Osterrieth Complex Figure Test – copy trial (< 12 years of education).

Test score	Age (years)									
	18-29 (*n* = 32)		30-49 (*n* = 70)		50-64 (*n* = 124)		65-74 (*n* = 159)		≥ 75 (*n* = 125)	
	PR	*z*-score	PR	*z*-score	PR	*z*-score	PR	*z*-score	PR	*z*-score
≤ 13.5	0	-3	0	-3	0	-3	0	-3	0	-3
14	0	-3	0	-3	0	-2.65	0	-3	0	-3
14.5	0	-3	0	-3	1	-2.41	0	-3	0	-3
15	0	-3	0	-3	1	-2.41	0	-2.74	0	-3
15.5	0	-3	0	-3	1	-2.41	1	-2.5	0	-3
16	0	-3	0	-3	1	-2.41	1	-2.35	1	-2.41
16.5	0	-3	0	-3	1	-2.41	1	-2.24	2	-2.15
17	0	-3	0	-3	1	-2.41	1	-2.24	2	-1.98
17.5	0	-3	0	-3	1	-2.41	1	-2.24	3	-1.86
18	0	-3	0	-3	1	-2.41	2	-2.16	4	-1.8
18.5	0	-3	0	-3	1	-2.41	2	-2.08	4	-1.76
19	0	-3	0	-3	1	-2.26	3	-1.96	4	-1.71
19.5	0	-3	0	-3	2	-2.15	3	-1.86	5	-1.67
20	0	-3	1	-2.45	2	-2.15	3	-1.86	5	-1.67
20.5	0	-3	1	-2.19	2	-2.15	3	-1.86	5	-1.67

Table S7. Percentile ranks and *z*-scores per age group for the Rey-Osterrieth Complex Figure Test – copy trial (< 12 years of education).

Test score	Age (years)									
	18-29 (*n* = 32)		30-49 (*n* = 70)		50-64 (*n* = 124)		65-74 (*n* = 159)		≥ 75 (*n* = 125)	
	PR	*z*-score	PR	*z*-score	PR	*z*-score	PR	*z*-score	PR	*z*-score
21	0	-3	1	-2.19	2	-2.05	4	-1.78	6	-1.56
21.5	0	-3	1	-2.19	2	-1.98	4	-1.71	7	-1.47
22	0	-3	2	-2.03	2	-1.98	4	-1.71	9	-1.33
22.5	0	-3	3	-1.91	2	-1.98	4	-1.71	11	-1.22
23	0	-3	3	-1.91	2	-1.98	5	-1.68	11	-1.22
23.5	0	-3	3	-1.91	2	-1.98	5	-1.65	11	-1.22
24	0	-3	3	-1.91	3	-1.91	5	-1.62	12	-1.16
24.5	0	-3	3	-1.91	3	-1.85	6	-1.59	14	-1.1
25	0	-3	4	-1.72	3	-1.85	6	-1.56	14	-1.1
25.5	0	-3	6	-1.58	3	-1.85	6	-1.54	14	-1.1
26	0	-3	6	-1.52	4	-1.8	7	-1.46	14	-1.09
26.5	0	-3	7	-1.47	4	-1.75	8	-1.4	14	-1.07
27	2	-2.16	9	-1.33	5	-1.67	8	-1.38	15	-1.05
27.5	3	-1.87	11	-1.21	6	-1.59	9	-1.36	15	-1.03
28	3	-1.87	11	-1.21	7	-1.49	11	-1.25	16	-1
28.5	3	-1.87	11	-1.21	8	-1.41	13	-1.15	17	-0.97
29	3	-1.87	11	-1.21	9	-1.35	14	-1.06	18	-0.91
29.5	3	-1.87	11	-1.21	10	-1.3	16	-0.98	20	-0.85
30	3	-1.87	11	-1.21	13	-1.16	19	-0.9	23	-0.74

Table S7. Percentile ranks and *z*-scores per age group for the Rey-Osterrieth Complex Figure Test – copy trial (< 12 years of education).

Test score	Age (years)									
	18-29 (*n* = 32)		30-49 (*n* = 70)		50-64 (*n* = 124)		65-74 (*n* = 159)		≥ 75 (*n* = 125)	
	PR	*z*-score	PR	*z*-score	PR	*z*-score	PR	*z*-score	PR	*z*-score
30.5	3	-1.87	11	-1.21	15	-1.03	21	-0.82	26	-0.64
31	3	-1.87	12	-1.17	16	-0.99	23	-0.74	30	-0.54
31.5	3	-1.87	13	-1.14	17	-0.96	25	-0.67	33	-0.45
32	8	-1.42	16	-0.98	22	-0.78	34	-0.43	36	-0.37
32.5	13	-1.16	20	-0.85	27	-0.63	42	-0.2	38	-0.3
33	16	-1.01	24	-0.73	30	-0.52	45	-0.12	43	-0.19
33.5	19	-0.89	27	-0.61	34	-0.42	48	-0.04	47	-0.08
34	27	-0.63	38	-0.31	40	-0.25	54	0.1	56	0.15
34.5	34	-0.41	49	-0.04	47	-0.09	60	0.24	65	0.38
35	39	-0.28	54	0.1	51	0.02	64	0.37	70	0.53
35.5	44	-0.16	60	0.25	55	0.12	69	0.5	76	0.7
36	72	0.57	80	0.84	77	0.75	85	1.01	88	1.17

White cells correspond to cognitive impairment. cells highlighted in gray represent normal cognition; **PR** Percentile rank.

Table S8. Percentile ranks and *z*-scores per age group for the Rey-Osterrieth Complex Figure Test – copy trial (≥ 12 years of education).

Test score	Age (years)									
	18-29 (*n* = 56)		30-49 (*n* = 110)		50-64 (*n* = 153)		65-74 (*n* = 150)		≥ 75 (*n* = 82)	
	PR	*z*-score	PR	*z*-score	PR	*z*-score	PR	*z*-score	PR	*z*-score
≤ 13.5	0	-3	0	-3	0	-3	0	-3	0	-3
14	0	-3	0	-3	0	-3	0	-2.72	0	-3
14.5	0	-3	0	-3	0	-3	1	-2.48	0	-3
15	0	-3	0	-3	0	-3	1	-2.48	1	-2.51
15.5	0	-3	0	-3	0	-3	1	-2.48	1	-2.25
16	0	-3	0	-3	0	-3	1	-2.33	1	-2.25
16.5	0	-3	0	-3	0	-3	1	-2.22	1	-2.25
17	0	-3	0	-3	0	-3	1	-2.22	2	-2.09
17.5	0	-3	0	-3	0	-3	1	-2.22	2	-1.97
18	0	-3	0	-3	0	-3	1	-2.22	2	-1.97
18.5	0	-3	0	-3	0	-3	1	-2.22	2	-1.97
19	0	-3	0	-3	0	-3	2	-2.13	2	-1.97
19.5	0	-3	0	-3	0	-3	2	-2.06	2	-1.97
20	0	-3	0	-3	0	-3	2	-1.99	2	-1.97
20.5	0	-3	0	-3	0	-3	3	-1.94	2	-1.97
21	0	-3	0	-3	0	-3	3	-1.94	2	-1.97
21.5	0	-3	0	-3	0	-3	3	-1.94	2	-1.97
22	0	-3	0	-3	0	-3	3	-1.94	2	-1.97
22.5	0	-3	0	-3	0	-3	3	-1.94	2	-1.97

Table S8. Percentile ranks and *z*-scores per age group for the Rey-Osterrieth Complex Figure Test – copy trial (≥ 12 years of education).

Test score	Age (years)									
	18-29 (*n* = 56)		30-49 (*n* = 110)		50-64 (*n* = 153)		65-74 (*n* = 150)		≥ 75 (*n* = 82)	
	PR	*z*-score	PR	*z*-score	PR	*z*-score	PR	*z*-score	PR	*z*-score
23	0	-3	0	-3	0	-3	3	-1.94	2	-1.97
23.5	0	-3	0	-3	0	-3	3	-1.94	2	-1.97
24	0	-3	0	-3	1	-2.49	3	-1.89	2	-1.97
24.5	0	-3	0	-3	1	-2.23	3	-1.84	2	-1.97
25	0	-3	0	-2.61	2	-2.07	4	-1.79	4	-1.8
25.5	0	-3	1	-2.36	3	-1.95	4	-1.76	5	-1.66
26	0	-3	1	-2.21	3	-1.85	5	-1.68	5	-1.66
26.5	0	-3	2	-2.1	4	-1.76	5	-1.62	5	-1.66
27	0	-3	2	-2.01	4	-1.73	6	-1.56	7	-1.5
27.5	0	-3	3	-1.93	5	-1.69	7	-1.51	9	-1.37
28	0	-3	3	-1.93	5	-1.66	8	-1.41	10	-1.27
28.5	0	-3	3	-1.93	5	-1.63	9	-1.33	12	-1.17
29	0	-3	3	-1.86	6	-1.54	10	-1.27	14	-1.08
29.5	0	-3	4	-1.8	7	-1.47	11	-1.21	16	-1.01
30	1	-2.37	6	-1.57	9	-1.36	13	-1.15	17	-0.96
30.5	2	-2.1	8	-1.4	10	-1.26	14	-1.09	18	-0.91
31	3	-1.93	9	-1.37	12	-1.19	15	-1.04	23	-0.74
31.5	4	-1.81	9	-1.34	13	-1.13	16	-1	28	-0.59
32	10	-1.3	12	-1.19	16	-1.01	20	-0.83	34	-0.41

Table S8. Percentile ranks and *z*-scores per age group for the Rey-Osterrieth Complex Figure Test – copy trial (≥ 12 years of education).

Test score	Age (years)									
	18-29 (*n* = 56)		30-49 (*n* = 110)		50-64 (*n* = 153)		65-74 (*n* = 150)		≥ 75 (*n* = 82)	
	PR	*z*-score	PR	*z*-score	PR	*z*-score	PR	*z*-score	PR	*z*-score
32.5	16	-1	15	-1.06	18	-0.91	25	-0.69	40	-0.25
33	17	-0.96	15	-1.02	20	-0.86	30	-0.54	43	-0.17
33.5	18	-0.93	16	-0.98	21	-0.81	35	-0.4	46	-0.1
34	23	-0.74	22	-0.77	28	-0.58	43	-0.17	54	0.1
34.5	29	-0.57	28	-0.58	35	-0.38	52	0.05	62	0.31
35	37	-0.35	31	-0.5	40	-0.26	55	0.11	69	0.49
35.5	45	-0.14	34	-0.43	44	-0.14	57	0.18	76	0.69
36	72	0.59	67	0.43	72	0.58	79	0.79	88	1.16

White cells correspond to cognitive impairment. cells highlighted in gray represent normal cognition; **PR** Percentile rank.

Rey-Osterrieth Complex Figure Test – Delayed Recall (30 minutes)

Males

Table S9. Percentile ranks and *z*-scores per age group for the Rey-Osterrieth Complex Figure Test – delayed recall (males, < 12 years of education).

Test score	Age (years)									
	18-29 (*n* = 17)		30-49 (*n* = 36)		50-64 (*n* = 66)		65-74 (*n* = 79)		≥ 75 (*n* = 64)	
	PR	*z*-score	PR	*z*-score	PR	*z*-score	PR	*z*-score	PR	*z*-score
0	0	-3	1	-2.2	0	-3	1	-2.24	4	-1.77
0.5	0	-3	3	-1.92	0	-3	3	-1.96	8	-1.42
1	0	-3	3	-1.92	0	-3	3	-1.96	8	-1.42
1.5	0	-3	3	-1.92	0	-3	3	-1.96	8	-1.42
2	0	-3	3	-1.92	1	-2.43	4	-1.71	9	-1.37
2.5	0	-3	3	-1.92	2	-2.17	6	-1.53	9	-1.32
3	0	-3	3	-1.92	3	-1.88	8	-1.44	12	-1.19
3.5	0	-3	3	-1.92	5	-1.69	9	-1.35	14	-1.08
4	0	-3	3	-1.92	5	-1.69	9	-1.32	15	-1.05
4.5	0	-3	3	-1.92	5	-1.69	10	-1.28	16	-1.01
5	0	-3	3	-1.92	5	-1.69	12	-1.18	19	-0.89
5.5	0	-3	3	-1.92	5	-1.69	14	-1.09	22	-0.78
6	0	-3	4	-1.74	5	-1.62	16	-1.01	23	-0.73
6.5	0	-3	6	-1.6	6	-1.55	18	-0.93	25	-0.68
7	0	-3	8	-1.39	10	-1.29	18	-0.91	27	-0.61
7.5	0	-3	11	-1.23	14	-1.1	19	-0.88	30	-0.54

Table S9. Percentile ranks and *z*-scores per age group for the Rey-Osterrieth Complex Figure Test – delayed recall (males, < 12 years of education).

Test score	Age (years)									
	18-29 (*n* = 17)		30-49 (*n* = 36)		50-64 (*n* = 66)		65-74 (*n* = 79)		≥ 75 (*n* = 64)	
	PR	*z*-score	PR	*z*-score	PR	*z*-score	PR	*z*-score	PR	*z*-score
8	0	-3	11	-1.23	15	-1.03	20	-0.86	32	-0.47
8.5	0	-3	11	-1.23	17	-0.97	20	-0.84	34	-0.41
9	3	-1.89	14	-1.09	17	-0.97	22	-0.79	34	-0.41
9.5	6	-1.57	17	-0.97	17	-0.97	23	-0.75	34	-0.41
10	6	-1.57	17	-0.97	18	-0.91	24	-0.71	36	-0.36
10.5	6	-1.57	17	-0.97	20	-0.86	25	-0.67	38	-0.32
11	6	-1.57	17	-0.97	21	-0.8	27	-0.63	38	-0.3
11.5	6	-1.57	17	-0.97	23	-0.75	28	-0.59	39	-0.28
12	9	-1.36	17	-0.97	25	-0.68	30	-0.52	45	-0.14
12.5	12	-1.19	17	-0.97	27	-0.61	33	-0.45	50	0
13	12	-1.19	17	-0.97	33	-0.46	35	-0.4	52	0.03
13.5	12	-1.19	17	-0.97	38	-0.31	37	-0.34	53	0.07
14	18	-0.93	17	-0.97	38	-0.31	44	-0.15	54	0.09
14.5	24	-0.73	17	-0.97	38	-0.31	52	0.04	55	0.11
15	24	-0.73	22	-0.77	39	-0.27	54	0.09	59	0.21
15.5	24	-0.73	28	-0.59	41	-0.23	56	0.14	63	0.31
16	24	-0.73	31	-0.51	47	-0.08	59	0.22	65	0.38
16.5	24	-0.73	33	-0.44	53	0.07	62	0.3	67	0.44
17	29	-0.55	38	-0.32	55	0.13	63	0.32	69	0.48

Table S9. Percentile ranks and *z*-scores per age group for the Rey-Osterrieth Complex Figure Test – delayed recall (males, < 12 years of education).

Test score	Age (years)									
	18-29 (*n* = 17)		30-49 (*n* = 36)		50-64 (*n* = 66)		65-74 (*n* = 79)		≥ 75 (*n* = 64)	
	PR	*z*-score	PR	*z*-score	PR	*z*-score	PR	*z*-score	PR	*z*-score
17.5	35	-0.38	42	-0.22	58	0.19	63	0.33	70	0.53
18	44	-0.15	43	-0.18	61	0.26	66	0.4	73	0.62
18.5	53	0.07	44	-0.14	64	0.34	68	0.47	77	0.72
19	56	0.14	46	-0.11	65	0.38	72	0.56	78	0.77
19.5	59	0.22	47	-0.07	67	0.43	75	0.66	80	0.83
20	68	0.45	49	-0.04	69	0.49	77	0.74	81	0.88
20.5	76	0.72	50	0	71	0.55	80	0.83	83	0.94
21	76	0.72	51	0.03	73	0.62	82	0.92	85	1.04
21.5	76	0.72	53	0.06	76	0.69	85	1.02	88	1.15
22	79	0.82	53	0.06	77	0.74	85	1.02	88	1.18
22.5	82	0.92	53	0.06	79	0.79	85	1.02	89	1.22
23	82	0.92	56	0.13	80	0.82	87	1.11	90	1.27
23.5	82	0.92	58	0.21	80	0.85	89	1.2	91	1.31
24	82	0.92	60	0.24	83	0.96	90	1.27	93	1.47
24.5	82	0.92	61	0.28	86	1.09	91	1.34	95	1.67
25	85	1.04	64	0.35	87	1.13	92	1.39	96	1.76
25.5	88	1.18	67	0.43	88	1.16	92	1.43	97	1.86
26	91	1.35	71	0.54	88	1.16	94	1.58	97	1.86
26.5	94	1.56	75	0.67	88	1.16	96	1.77	97	1.86

Table S9. Percentile ranks and *z*-scores per age group for the Rey-Osterrieth Complex Figure Test – delayed recall (males, < 12 years of education).

Test score	Age (years)									
	18-29 (*n* = 17)		30-49 (*n* = 36)		50-64 (*n* = 66)		65-74 (*n* = 79)		≥ 75 (*n* = 64)	
	PR	*z*-score	PR	*z*-score	PR	*z*-score	PR	*z*-score	PR	*z*-score
27	94	1.56	79	0.81	89	1.24	96	1.77	97	1.86
27.5	94	1.56	83	0.96	91	1.33	96	1.77	97	1.86
28	94	1.56	85	1.02	92	1.38	96	1.77	97	1.86
28.5	94	1.56	86	1.08	92	1.43	96	1.77	97	1.86
29	94	1.56	86	1.08	92	1.43	97	1.95	97	1.86
29.5	94	1.56	86	1.08	92	1.43	99	2.23	97	1.86
30	94	1.56	88	1.15	93	1.48	99	2.23	97	1.86
30.5	94	1.56	89	1.22	94	1.55	99	2.23	97	1.86
31	94	1.56	92	1.38	95	1.61	99	2.23	98	2.15
31.5	94	1.56	94	1.59	95	1.69	99	2.23	100	3
32	94	1.56	97	1.91	97	1.87	99	2.49	100	3
32.5	94	1.56	100	3	98	2.16	100	3	100	3
33	94	1.56	100	3	98	2.16	100	3	100	3
33.5	94	1.56	100	3	98	2.16	100	3	100	3
34	94	1.56	100	3	99	2.42	100	3	100	3
34.5	94	1.56	100	3	100	3	100	3	100	3
35	97	1.89	100	3	100	3	100	3	100	3
≥ 35.5	100	3	100	3	100	3	100	3	100	3

White cells correspond to cognitive impairment. cells highlighted in gray represent normal cognition; **PR** Percentile rank.

Table S10. Percentile ranks and *z*-scores per age group for the Rey-Osterrieth Complex Figure Test – delayed recall (males, ≥ 12 years of education).

Test score	Age (years)									
	18-29 (*n* = 26)		30-49 (*n* = 63)		50-64 (*n* = 80)		65-74 (*n* = 96)		≥ 75 (*n* = 51)	
	PR	*z*-score	PR	*z*-score	PR	*z*-score	PR	*z*-score	PR	*z*-score
0	0	-3	1	-2.42	0	-3	1	-2.31	1	-2.34
0.5	0	-3	2	-2.15	0	-3	2	-2.04	2	-2.07
1	0	-3	2	-2.15	1	-2.5	2	-2.04	3	-1.89
1.5	0	-3	2	-2.15	1	-2.24	2	-2.04	4	-1.76
2	2	-2.07	2	-2.15	2	-2.08	3	-1.87	8	-1.42
2.5	4	-1.77	2	-2.15	3	-1.96	4	-1.74	12	-1.19
3	4	-1.77	2	-2.15	3	-1.96	5	-1.68	13	-1.14
3.5	4	-1.77	2	-2.15	3	-1.96	5	-1.63	14	-1.1
4	4	-1.77	2	-2.15	3	-1.96	6	-1.54	14	-1.1
4.5	4	-1.77	2	-2.15	3	-1.96	7	-1.46	14	-1.1
5	4	-1.77	2	-1.99	3	-1.96	8	-1.39	15	-1.05
5.5	4	-1.77	3	-1.86	3	-1.96	9	-1.32	16	-1.01
6	4	-1.77	3	-1.86	4	-1.78	10	-1.26	16	-1.01
6.5	4	-1.77	3	-1.86	5	-1.65	11	-1.21	16	-1.01
7	4	-1.77	3	-1.86	6	-1.59	13	-1.13	17	-0.97
7.5	4	-1.77	3	-1.86	6	-1.54	15	-1.06	18	-0.93
8	4	-1.77	5	-1.67	6	-1.54	16	-0.99	23	-0.76
8.5	4	-1.77	6	-1.53	6	-1.54	18	-0.93	27	-0.6

Table S10. Percentile ranks and *z*-scores per age group for the Rey-Osterrieth Complex Figure Test – delayed recall (males, ≥ 12 years of education).

Test score	Age (years)									
	18-29 (*n* = 26)		30-49 (*n* = 63)		50-64 (*n* = 80)		65-74 (*n* = 96)		≥ 75 (*n* = 51)	
	PR	*z*-score	PR	*z*-score	PR	*z*-score	PR	*z*-score	PR	*z*-score
9	4	-1.77	10	-1.31	6	-1.54	20	-0.84	28	-0.57
9.5	4	-1.77	13	-1.15	6	-1.54	23	-0.75	29	-0.55
10	6	-1.58	13	-1.15	7	-1.49	26	-0.65	32	-0.46
10.5	8	-1.43	13	-1.15	8	-1.44	29	-0.55	35	-0.38
11	8	-1.43	14	-1.07	9	-1.36	31	-0.49	37	-0.33
11.5	8	-1.43	16	-1	10	-1.29	33	-0.44	39	-0.28
12	10	-1.31	17	-0.97	11	-1.25	35	-0.39	41	-0.23
12.5	12	-1.2	17	-0.94	11	-1.22	36	-0.35	43	-0.18
13	12	-1.2	19	-0.88	13	-1.13	39	-0.3	44	-0.15
13.5	12	-1.2	21	-0.82	15	-1.04	41	-0.24	45	-0.13
14	13	-1.11	21	-0.8	20	-0.85	42	-0.22	48	-0.05
14.5	15	-1.03	22	-0.77	25	-0.68	43	-0.19	51	0.02
15	15	-1.03	26	-0.64	25	-0.68	45	-0.14	54	0.09
15.5	15	-1.03	30	-0.52	25	-0.68	47	-0.08	57	0.17
16	15	-1.03	31	-0.5	26	-0.66	51	0.01	59	0.22
16.5	15	-1.03	32	-0.48	26	-0.64	54	0.1	61	0.27
17	15	-1.03	33	-0.44	28	-0.58	57	0.18	63	0.32
17.5	15	-1.03	35	-0.39	30	-0.53	60	0.26	65	0.37
18	17	-0.95	36	-0.37	35	-0.39	63	0.33	71	0.54

Table S10. Percentile ranks and *z*-scores per age group for the Rey-Osterrieth Complex Figure Test – delayed recall (males, ≥ 12 years of education).

Test score	Age (years)									
	18-29 (*n* = 26)		30-49 (*n* = 63)		50-64 (*n* = 80)		65-74 (*n* = 96)		≥ 75 (*n* = 51)	
	PR	*z*-score	PR	*z*-score	PR	*z*-score	PR	*z*-score	PR	*z*-score
18.5	19	-0.87	37	-0.35	40	-0.26	66	0.4	76	0.72
19	19	-0.87	40	-0.27	44	-0.15	68	0.45	77	0.75
19.5	19	-0.87	43	-0.18	49	-0.04	70	0.51	78	0.78
20	19	-0.87	45	-0.12	51	0.01	72	0.59	79	0.82
20.5	19	-0.87	48	-0.06	53	0.06	75	0.67	80	0.85
21	23	-0.74	52	0.03	56	0.14	76	0.7	84	1
21.5	27	-0.62	56	0.13	59	0.22	77	0.74	88	1.18
22	33	-0.45	56	0.15	64	0.35	80	0.83	90	1.29
22.5	38	-0.3	57	0.18	69	0.48	82	0.92	92	1.41
23	40	-0.25	62	0.3	73	0.59	83	0.94	93	1.48
23.5	42	-0.2	67	0.43	76	0.71	83	0.96	94	1.56
24	48	-0.05	69	0.49	79	0.79	85	1.03	94	1.56
24.5	54	0.09	71	0.56	81	0.88	86	1.1	94	1.56
25	60	0.24	74	0.63	84	0.98	86	1.1	95	1.65
25.5	65	0.39	76	0.71	86	1.09	86	1.1	96	1.76
26	67	0.44	79	0.79	88	1.15	88	1.17	96	1.76
26.5	69	0.5	81	0.87	89	1.21	90	1.25	96	1.76
27	71	0.55	84	1	89	1.24	90	1.28	96	1.76
27.5	73	0.61	87	1.14	90	1.28	91	1.31	96	1.76

Table S10. Percentile ranks and *z*-scores per age group for the Rey-Osterrieth Complex Figure Test – delayed recall (males, ≥ 12 years of education).

Test score	Age (years)									
	18-29 (*n* = 26)		30-49 (*n* = 63)		50-64 (*n* = 80)		65-74 (*n* = 96)		≥ 75 (*n* = 51)	
	PR	*z*-score	PR	*z*-score	PR	*z*-score	PR	*z*-score	PR	*z*-score
28	79	0.8	89	1.22	93	1.48	92	1.38	97	1.89
28.5	85	1.02	90	1.3	96	1.78	93	1.45	98	2.06
29	88	1.19	92	1.4	96	1.78	94	1.53	99	2.33
29.5	92	1.42	94	1.52	96	1.78	95	1.62	100	3
30	94	1.57	94	1.59	98	1.96	95	1.62	100	3
30.5	96	1.76	95	1.66	99	2.24	95	1.62	100	3
31	96	1.76	97	1.85	99	2.24	97	1.86	100	3
31.5	96	1.76	98	2.14	99	2.24	99	2.31	100	3
32	96	1.76	98	2.14	99	2.24	99	2.31	100	3
32.5	96	1.76	98	2.14	99	2.24	99	2.31	100	3
33	96	1.76	99	2.41	99	2.24	99	2.56	100	3
33.5	96	1.76	100	3	99	2.24	100	3	100	3
34	98	2.07	100	3	99	2.5	100	3	100	3
≥ 34.5	100	3	100	3	100	3	100	3	100	3

White cells correspond to cognitive impairment. cells highlighted in gray represent normal cognition; **PR** Percentile rank.

Females

Table S11. Percentile ranks and *z*-scores per age group for the Rey-Osterrieth Complex Figure Test – delayed recall (females, < 12 years of education).

Test score	Age (years)									
	18-29 (*n* = 12)		30-49 (*n* = 33)		50-64 (*n* = 59)		65-74 (*n* = 78)		≥ 75 (*n* = 65)	
	PR	*z*-score	PR	*z*-score	PR	*z*-score	PR	*z*-score	PR	*z*-score
0	4	-1.74	0	-3	1	-2.39	4	-1.7	6	-1.55
0.5	8	-1.39	0	-3	2	-2.13	9	-1.35	12	-1.16
1	8	-1.39	0	-3	3	-1.96	10	-1.31	14	-1.09
1.5	8	-1.39	0	-3	3	-1.83	10	-1.27	15	-1.03
2	8	-1.39	0	-3	3	-1.83	10	-1.27	16	-0.99
2.5	8	-1.39	0	-3	3	-1.83	10	-1.27	17	-0.96
3	8	-1.39	0	-3	3	-1.83	12	-1.2	18	-0.9
3.5	8	-1.39	0	-3	3	-1.83	13	-1.14	20	-0.85
4	8	-1.39	0	-3	3	-1.83	13	-1.14	22	-0.79
4.5	8	-1.39	0	-3	3	-1.83	13	-1.11	23	-0.74
5	8	-1.39	0	-3	4	-1.73	16	-1	25	-0.69
5.5	8	-1.39	0	-3	5	-1.64	18	-0.92	27	-0.62
6	8	-1.39	0	-3	5	-1.64	18	-0.92	31	-0.51
6.5	8	-1.39	0	-3	6	-1.57	18	-0.92	35	-0.4
7	8	-1.39	0	-3	8	-1.44	19	-0.9	36	-0.36
7.5	8	-1.39	0	-3	8	-1.38	21	-0.81	37	-0.34
8	8	-1.39	0	-3	13	-1.14	26	-0.64	40	-0.26

Table S11. Percentile ranks and *z*-scores per age group for the Rey-Osterrieth Complex Figure Test – delayed recall (females, < 12 years of education).

Test score	Age (years)									
	18-29 (*n* = 12)		30-49 (*n* = 33)		50-64 (*n* = 59)		65-74 (*n* = 78)		≥ 75 (*n* = 65)	
	PR	*z*-score	PR	*z*-score	PR	*z*-score	PR	*z*-score	PR	*z*-score
8.5	8	-1.39	0	-3	17	-0.96	29	-0.54	44	-0.16
9	8	-1.39	5	-1.69	18	-0.93	32	-0.47	45	-0.12
9.5	8	-1.39	11	-1.25	19	-0.9	35	-0.4	46	-0.1
10	13	-1.16	12	-1.17	23	-0.75	36	-0.37	48	-0.06
10.5	21	-0.82	17	-0.97	27	-0.61	38	-0.3	51	0.01
11	25	-0.68	21	-0.8	30	-0.54	40	-0.25	52	0.05
11.5	25	-0.68	21	-0.8	33	-0.44	42	-0.22	53	0.07
12	25	-0.68	24	-0.7	36	-0.35	45	-0.13	57	0.17
12.5	25	-0.68	29	-0.56	39	-0.28	48	-0.05	61	0.27
13	29	-0.55	32	-0.48	43	-0.18	50	0	63	0.33
13.5	33	-0.44	33	-0.44	48	-0.05	53	0.06	65	0.39
14	38	-0.32	38	-0.31	50	0	57	0.17	70	0.52
14.5	42	-0.22	42	-0.2	51	0.02	61	0.27	74	0.63
15	42	-0.22	44	-0.16	55	0.12	65	0.39	76	0.71
15.5	42	-0.22	47	-0.08	60	0.25	71	0.53	79	0.81
16	42	-0.22	50	0	63	0.32	73	0.61	82	0.89
16.5	42	-0.22	52	0.03	65	0.39	74	0.65	85	1.05
17	42	-0.22	55	0.11	66	0.41	74	0.65	88	1.19
17.5	42	-0.22	59	0.22	66	0.41	75	0.67	89	1.23

Table S11. Percentile ranks and *z*-scores per age group for the Rey-Osterrieth Complex Figure Test – delayed recall (females, < 12 years of education).

Test score	Age (years)									
	18-29 (*n* = 12)		30-49 (*n* = 33)		50-64 (*n* = 59)		65-74 (*n* = 78)		≥ 75 (*n* = 65)	
	PR	*z*-score	PR	*z*-score	PR	*z*-score	PR	*z*-score	PR	*z*-score
18	42	-0.22	64	0.34	66	0.41	77	0.73	91	1.32
18.5	42	-0.22	67	0.43	67	0.43	79	0.8	93	1.48
19	42	-0.22	68	0.47	69	0.48	80	0.84	94	1.54
19.5	42	-0.22	71	0.55	69	0.5	81	0.86	94	1.54
20	42	-0.22	73	0.6	71	0.55	81	0.89	95	1.6
20.5	46	-0.11	73	0.6	73	0.6	83	0.94	96	1.76
21	50	0	79	0.79	78	0.77	85	1.02	97	1.86
21.5	50	0	85	1.03	83	0.95	86	1.07	98	1.99
22	54	0.1	86	1.09	83	0.95	87	1.1	98	2.16
22.5	58	0.21	88	1.16	83	0.95	87	1.13	98	2.16
23	63	0.31	88	1.16	86	1.06	88	1.19	98	2.16
23.5	67	0.43	89	1.24	89	1.22	90	1.26	98	2.16
24	71	0.54	91	1.33	91	1.32	90	1.3	99	2.42
24.5	75	0.67	91	1.33	92	1.43	92	1.38	100	3
25	75	0.67	91	1.33	93	1.49	94	1.57	100	3
25.5	79	0.81	91	1.33	93	1.49	96	1.76	100	3
26	83	0.96	91	1.33	95	1.63	96	1.76	100	3
26.5	83	0.96	91	1.33	97	1.82	96	1.76	100	3
27	92	1.38	94	1.55	97	1.95	97	1.95	100	3

Table S11. Percentile ranks and *z*-scores per age group for the Rey-Osterrieth Complex Figure Test – delayed recall (females, < 12 years of education).

Test score	Age (years)									
	18-29 (*n* = 12)		30-49 (*n* = 33)		50-64 (*n* = 59)		65-74 (*n* = 78)		≥ 75 (*n* = 65)	
	PR	*z*-score	PR	*z*-score	PR	*z*-score	PR	*z*-score	PR	*z*-score
27.5	100	3	97	1.87	98	2.12	99	2.23	100	3
28	100	3	97	1.87	98	2.12	99	2.49	100	3
28.5	100	3	97	1.87	98	2.12	100	3	100	3
29	100	3	98	2.16	98	2.12	100	3	100	3
29.5	100	3	100	3	98	2.12	100	3	100	3
30	100	3	100	3	98	2.12	100	3	100	3
30.5	100	3	100	3	98	2.12	100	3	100	3
31	100	3	100	3	98	2.12	100	3	100	3
31.5	100	3	100	3	99	2.38	100	3	100	3
≥ 32	100	3	100	3	100	3	100	3	100	3

White cells correspond to cognitive impairment. cells highlighted in gray represent normal cognition; **PR** Percentile rank.

Table S12. Percentile ranks and *z*-scores per age group for the Rey-Osterrieth Complex Figure Test – delayed recall (females, ≥ 12 years of education).

Test score	Age (years)									
	18-29 (*n* = 28)		30-49 (*n* = 46)		50-64 (*n* = 72)		65-74 (*n* = 51)		≥ 75 (*n* = 32)	
	PR	*z*-score	PR	*z*-score	PR	*z*-score	PR	*z*-score	PR	*z*-score
0	0	-3	0	-3	1	-2.46	2	-2.07	2	-2.16
0.5	0	-3	0	-3	1	-2.2	4	-1.76	3	-1.87
1	0	-3	0	-3	1	-2.2	4	-1.76	3	-1.87
1.5	0	-3	0	-3	1	-2.2	4	-1.76	3	-1.87
2	0	-3	0	-3	1	-2.2	5	-1.66	5	-1.68
2.5	0	-3	0	-3	1	-2.2	6	-1.57	6	-1.54
3	2	-2.1	0	-3	1	-2.2	6	-1.57	8	-1.42
3.5	4	-1.81	0	-3	1	-2.2	6	-1.57	9	-1.32
4	5	-1.62	0	-3	1	-2.2	8	-1.42	11	-1.23
4.5	7	-1.47	0	-3	1	-2.2	12	-1.19	13	-1.16
5	9	-1.35	0	-3	2	-2.04	15	-1.05	16	-1.01
5.5	11	-1.25	0	-3	3	-1.92	17	-0.97	19	-0.89
6	11	-1.25	0	-3	3	-1.82	19	-0.9	19	-0.89
6.5	11	-1.25	0	-3	5	-1.66	22	-0.79	20	-0.84
7	11	-1.25	0	-3	7	-1.48	25	-0.69	23	-0.73
7.5	11	-1.25	1	-2.3	8	-1.39	25	-0.66	27	-0.63
8	11	-1.25	4	-1.72	9	-1.34	27	-0.6	30	-0.54
8.5	11	-1.25	7	-1.52	10	-1.3	29	-0.55	31	-0.49
9	11	-1.25	8	-1.44	11	-1.23	32	-0.46	33	-0.45

Table S12. Percentile ranks and *z*-scores per age group for the Rey-Osterrieth Complex Figure Test – delayed recall (females, ≥ 12 years of education).

Test score	Age (years)									
	18-29 (*n* = 28)		30-49 (*n* = 46)		50-64 (*n* = 72)		65-74 (*n* = 51)		≥ 75 (*n* = 32)	
	PR	*z*-score	PR	*z*-score	PR	*z*-score	PR	*z*-score	PR	*z*-score
9.5	11	-1.25	9	-1.36	13	-1.16	35	-0.38	34	-0.41
10	11	-1.25	13	-1.13	15	-1.06	36	-0.36	34	-0.41
10.5	11	-1.25	18	-0.9	18	-0.92	38	-0.3	34	-0.41
11	11	-1.25	22	-0.79	21	-0.82	41	-0.23	36	-0.36
11.5	11	-1.25	24	-0.71	23	-0.75	43	-0.18	39	-0.28
12	11	-1.25	26	-0.65	26	-0.64	45	-0.13	42	-0.2
12.5	11	-1.25	28	-0.58	29	-0.55	49	-0.03	44	-0.16
13	14	-1.07	29	-0.55	33	-0.45	53	0.07	48	-0.04
13.5	18	-0.93	30	-0.52	36	-0.36	55	0.12	53	0.07
14	21	-0.8	30	-0.52	38	-0.31	56	0.14	53	0.07
14.5	25	-0.68	30	-0.52	41	-0.23	58	0.19	53	0.07
15	25	-0.68	32	-0.49	44	-0.16	65	0.37	58	0.19
15.5	25	-0.68	33	-0.46	47	-0.09	71	0.54	63	0.31
16	27	-0.62	37	-0.34	51	0.01	73	0.59	66	0.4
16.5	30	-0.52	42	-0.2	54	0.1	75	0.65	69	0.48
17	32	-0.47	45	-0.14	56	0.15	78	0.78	73	0.62
17.5	34	-0.42	47	-0.09	60	0.26	82	0.92	78	0.77
18	38	-0.32	49	-0.03	66	0.41	85	1.04	80	0.83
18.5	39	-0.28	51	0.02	70	0.52	88	1.18	83	0.94

Table S12. Percentile ranks and *z*-scores per age group for the Rey-Osterrieth Complex Figure Test – delayed recall (females, ≥ 12 years of education).

Test score	Age (years)									
	18-29 (*n* = 28)		30-49 (*n* = 46)		50-64 (*n* = 72)		65-74 (*n* = 51)		≥ 75 (*n* = 32)	
	PR	*z*-score	PR	*z*-score	PR	*z*-score	PR	*z*-score	PR	*z*-score
19	41	-0.23	53	0.08	72	0.58	90	1.29	88	1.15
19.5	43	-0.18	54	0.1	74	0.65	93	1.48	91	1.31
20	43	-0.18	57	0.16	77	0.74	94	1.56	91	1.31
20.5	43	-0.18	59	0.21	79	0.81	95	1.65	91	1.31
21	45	-0.14	60	0.24	81	0.86	97	1.89	92	1.41
21.5	46	-0.09	61	0.27	84	0.99	98	2.06	94	1.53
22	48	-0.05	61	0.27	88	1.15	98	2.06	94	1.53
22.5	50	0	64	0.36	89	1.22	98	2.06	94	1.53
23	50	0	68	0.48	90	1.29	99	2.33	95	1.67
23.5	50	0	71	0.54	92	1.38	100	3	97	1.86
24	52	0.04	74	0.64	92	1.43	100	3	97	1.86
24.5	55	0.13	77	0.74	93	1.48	100	3	97	1.86
25	57	0.18	80	0.85	94	1.53	100	3	97	1.86
25.5	59	0.22	83	0.93	94	1.59	100	3	97	1.86
26	61	0.27	86	1.07	95	1.65	100	3	97	1.86
26.5	61	0.27	89	1.23	96	1.73	100	3	97	1.86
27	63	0.31	89	1.23	96	1.73	100	3	98	2.15
27.5	64	0.36	89	1.23	96	1.73	100	3	100	3
28	66	0.41	91	1.35	97	1.81	100	3	100	3

Table S12. Percentile ranks and *z*-scores per age group for the Rey-Osterrieth Complex Figure Test – delayed recall (females, ≥ 12 years of education).

Test score	Age (years)									
	18-29 (*n* = 28)		30-49 (*n* = 46)		50-64 (*n* = 72)		65-74 (*n* = 51)		≥ 75 (*n* = 32)	
	PR	*z*-score	PR	*z*-score	PR	*z*-score	PR	*z*-score	PR	*z*-score
28.5	70	0.51	93	1.51	97	1.91	100	3	100	3
29	75	0.67	95	1.6	98	2.03	100	3	100	3
29.5	80	0.85	96	1.71	99	2.2	100	3	100	3
30	84	0.99	97	1.84	99	2.46	100	3	100	3
30.5	86	1.06	98	2.02	100	3	100	3	100	3
31	88	1.15	98	2.02	100	3	100	3	100	3
31.5	89	1.24	98	2.02	100	3	100	3	100	3
32	89	1.24	98	2.02	100	3	100	3	100	3
32.5	89	1.24	98	2.02	100	3	100	3	100	3
33	91	1.34	99	2.29	100	3	100	3	100	3
33.5	93	1.46	100	3	100	3	100	3	100	3
34	95	1.61	100	3	100	3	100	3	100	3
34.5	96	1.8	100	3	100	3	100	3	100	3
35	96	1.8	100	3	100	3	100	3	100	3
35.5	98	2.1	100	3	100	3	100	3	100	3
36	100	3	100	3	100	3	100	3	100	3

White cells correspond to cognitive impairment. cells highlighted in gray represent normal cognition; **PR** Percentile rank.

Trail Making Test, Part A

Table S13. Percentile ranks and *z*-scores per age group for the Trail Making Test, Part A (< 12 years of education).

Test score (sec)	Age (years)											
	18-29 (*n* = 38)		30-49 (*n* = 87)		50-59 (*n* = 91)		60-69 (*n* = 180)		70-79 (*n* = 226)		≥ 80 (*n* = 60)	
	PR	*z*-score	PR	*z*-score	PR	*z*-score	PR	*z*-score	PR	*z*-score	PR	*z*-score
≤ 10	100	3	100	3	100	3	100	3	100	3	100	3
11	100	3	100	3	100	3	100	3	100	2.85	100	3
12	100	3	100	3	100	3	100	3	100	2.62	100	3
13	100	3	99	2.53	100	3	100	3	100	2.62	100	3
14	100	3	99	2.27	100	3	100	3	100	2.62	100	3
15	100	3	99	2.27	100	3	100	2.77	100	2.62	100	3
16	100	3	99	2.27	100	3	99	2.53	100	2.62	100	3
17	100	3	98	2.11	100	3	99	2.39	100	2.62	100	3
18	96	1.73	97	1.81	100	3	99	2.28	100	2.62	100	3
19	88	1.15	95	1.68	100	3	99	2.28	100	2.62	100	3
20	79	0.81	94	1.57	99	2.54	99	2.2	100	2.62	100	3
21	75	0.67	92	1.4	98	2.13	98	2.12	100	2.62	100	3
22	75	0.67	89	1.2	97	1.91	98	2.01	100	2.62	100	3
23	75	0.67	85	1.03	96	1.76	97	1.83	100	2.62	100	3
24	71	0.54	83	0.96	94	1.55	96	1.7	99	2.48	100	3
25	63	0.31	81	0.87	90	1.25	95	1.61	99	2.28	100	3
26	58	0.21	77	0.73	86	1.09	94	1.52	99	2.21	100	3

Table S13. Percentile ranks and *z*-scores per age group for the Trail Making Test, Part A (< 12 years of education).

Test score (sec)	Age (years)											
	18-29 (*n* = 38)		30-49 (*n* = 87)		50-59 (*n* = 91)		60-69 (*n* = 180)		70-79 (*n* = 226)		≥ 80 (*n* = 60)	
	PR	*z*-score	PR	*z*-score	PR	*z*-score	PR	*z*-score	PR	*z*-score	PR	*z*-score
27	58	0.21	72	0.59	85	1.04	93	1.43	98	2.1	100	3
28	58	0.21	70	0.51	85	1.02	92	1.42	98	1.97	100	3
29	54	0.1	67	0.44	81	0.88	92	1.4	97	1.89	100	3
30	46	-0.11	64	0.36	76	0.7	91	1.31	96	1.77	100	3
31	42	-0.22	60	0.24	73	0.59	89	1.22	95	1.61	100	3
32	42	-0.22	56	0.15	70	0.53	87	1.13	94	1.53	99	2.39
33	42	-0.22	55	0.11	68	0.45	86	1.06	93	1.5	98	2.12
34	42	-0.22	51	0.02	65	0.38	85	1.02	92	1.42	98	2.12
35	42	-0.22	46	-0.11	63	0.33	83	0.96	91	1.35	98	2.12
36	38	-0.32	41	-0.22	62	0.3	81	0.89	90	1.27	97	1.83
37	33	-0.44	40	-0.25	61	0.27	79	0.81	88	1.2	94	1.56
38	29	-0.55	40	-0.25	59	0.22	76	0.71	87	1.12	93	1.43
39	25	-0.68	40	-0.27	56	0.15	73	0.61	85	1.05	91	1.33
40	21	-0.82	37	-0.33	53	0.08	71	0.54	84	0.98	90	1.28
41	17	-0.97	34	-0.4	49	-0.03	68	0.45	82	0.91	90	1.28
42	17	-0.97	32	-0.47	44	-0.16	64	0.36	80	0.85	90	1.28
43	17	-0.97	28	-0.58	41	-0.24	62	0.29	79	0.79	90	1.28
44	17	-0.97	25	-0.67	38	-0.3	60	0.25	76	0.7	90	1.28
45	17	-0.97	25	-0.67	37	-0.34	58	0.19	74	0.64	90	1.28

Table S13. Percentile ranks and *z*-scores per age group for the Trail Making Test, Part A (< 12 years of education).

Test score (sec)	Age (years)											
	18-29 (*n* = 38)		30-49 (*n* = 87)		50-59 (*n* = 91)		60-69 (*n* = 180)		70-79 (*n* = 226)		≥ 80 (*n* = 60)	
	PR	*z*-score	PR	*z*-score	PR	*z*-score	PR	*z*-score	PR	*z*-score	PR	*z*-score
46	17	-0.97	24	-0.73	35	-0.4	56	0.14	73	0.6	89	1.23
47	17	-0.97	22	-0.78	32	-0.48	54	0.09	71	0.55	87	1.11
48	17	-0.97	21	-0.8	30	-0.54	51	0.01	70	0.52	85	1.03
49	17	-0.97	18	-0.91	28	-0.59	48	-0.05	68	0.46	83	0.93
50	17	-0.97	16	-1.02	27	-0.62	44	-0.14	65	0.37	80	0.84
51	17	-0.97	14	-1.07	26	-0.65	42	-0.22	62	0.31	79	0.81
52	17	-0.97	12	-1.18	25	-0.67	40	-0.27	61	0.27	77	0.72
53	17	-0.97	9	-1.37	25	-0.69	38	-0.32	59	0.21	74	0.64
54	17	-0.97	7	-1.49	24	-0.71	36	-0.36	57	0.16	73	0.62
55	17	-0.97	7	-1.49	23	-0.74	34	-0.42	55	0.11	73	0.62
56	17	-0.97	7	-1.49	21	-0.8	33	-0.46	52	0.05	72	0.57
57	13	-1.16	7	-1.49	21	-0.82	32	-0.48	50	-0.02	68	0.47
58	8	-1.39	7	-1.49	20	-0.83	31	-0.51	47	-0.08	64	0.36
59	8	-1.39	7	-1.49	19	-0.87	30	-0.52	46	-0.1	58	0.18
60	8	-1.39	6	-1.58	18	-0.94	30	-0.54	45	-0.13	53	0.06
61	8	-1.39	5	-1.69	16	-1	29	-0.56	43	-0.17	51	0.02
62	8	-1.39	4	-1.75	14	-1.07	28	-0.6	42	-0.21	49	-0.03
63	8	-1.39	3	-1.82	13	-1.15	26	-0.65	41	-0.23	48	-0.05
64	8	-1.39	3	-1.82	12	-1.18	24	-0.71	40	-0.27	47	-0.09

Table S13. Percentile ranks and *z*-scores per age group for the Trail Making Test, Part A (< 12 years of education).

Test score (sec)	Age (years)											
	18-29 (*n* = 38)		30-49 (*n* = 87)		50-59 (*n* = 91)		60-69 (*n* = 180)		70-79 (*n* = 226)		≥ 80 (*n* = 60)	
	PR	*z*-score	PR	*z*-score	PR	*z*-score	PR	*z*-score	PR	*z*-score	PR	*z*-score
65	8	-1.39	3	-1.9	12	-1.18	23	-0.76	38	-0.32	44	-0.15
66	8	-1.39	2	-2	12	-1.18	22	-0.78	35	-0.4	43	-0.19
67	8	-1.39	2	-2	12	-1.18	20	-0.84	33	-0.45	41	-0.24
68	8	-1.39	2	-2	12	-1.18	19	-0.9	32	-0.48	38	-0.32
69	4	-1.74	2	-2.12	12	-1.18	18	-0.93	31	-0.51	35	-0.39
70	0	-3	1	-2.28	12	-1.2	17	-0.97	29	-0.55	35	-0.39
71	0	-3	1	-2.28	11	-1.23	16	-0.99	28	-0.6	35	-0.39
72	0	-3	1	-2.28	11	-1.23	16	-0.99	26	-0.65	34	-0.41
73	0	-3	1	-2.28	10	-1.26	16	-1.02	24	-0.71	33	-0.46
74	0	-3	1	-2.28	10	-1.29	15	-1.04	23	-0.74	31	-0.51
75	0	-3	1	-2.28	9	-1.32	15	-1.05	22	-0.78	29	-0.55
76	0	-3	1	-2.53	9	-1.36	14	-1.08	21	-0.81	28	-0.58
77	0	-3	0	-3	9	-1.36	14	-1.09	20	-0.83	28	-0.58
78	0	-3	0	-3	9	-1.36	14	-1.1	19	-0.87	28	-0.58
79	0	-3	0	-3	9	-1.36	13	-1.13	19	-0.89	28	-0.58
80	0	-3	0	-3	8	-1.39	13	-1.16	18	-0.92	28	-0.58
81	0	-3	0	-3	8	-1.43	12	-1.18	17	-0.95	28	-0.58
82	0	-3	0	-3	8	-1.43	11	-1.21	17	-0.97	28	-0.58
83	0	-3	0	-3	8	-1.43	11	-1.23	16	-0.98	28	-0.58

Table S13. Percentile ranks and *z*-scores per age group for the Trail Making Test, Part A (< 12 years of education).

Test score (sec)	Age (years)											
	18-29 (*n* = 38)		30-49 (*n* = 87)		50-59 (*n* = 91)		60-69 (*n* = 180)		70-79 (*n* = 226)		≥ 80 (*n* = 60)	
	PR	*z*-score	PR	*z*-score	PR	*z*-score	PR	*z*-score	PR	*z*-score	PR	*z*-score
84	0	-3	0	-3	8	-1.43	11	-1.24	16	-0.99	28	-0.58
85	0	-3	0	-3	8	-1.43	11	-1.25	16	-1.01	28	-0.58
86	0	-3	0	-3	8	-1.43	10	-1.3	15	-1.03	28	-0.6
87	0	-3	0	-3	7	-1.47	9	-1.35	15	-1.04	26	-0.65
88	0	-3	0	-3	6	-1.56	9	-1.37	15	-1.06	25	-0.68
89	0	-3	0	-3	5	-1.6	8	-1.39	13	-1.11	25	-0.68
90	0	-3	0	-3	5	-1.65	8	-1.42	12	-1.16	23	-0.73
91	0	-3	0	-3	4	-1.77	7	-1.48	12	-1.18	22	-0.79
92	0	-3	0	-3	3	-1.84	6	-1.53	12	-1.18	21	-0.82
93	0	-3	0	-3	3	-1.84	6	-1.57	12	-1.18	20	-0.85
94	0	-3	0	-3	3	-1.84	5	-1.62	11	-1.22	20	-0.85
95	0	-3	0	-3	3	-1.84	5	-1.65	10	-1.26	20	-0.85
96	0	-3	0	-3	3	-1.84	5	-1.65	10	-1.29	20	-0.85
97	0	-3	0	-3	3	-1.84	5	-1.65	10	-1.3	20	-0.85
98	0	-3	0	-3	3	-1.84	5	-1.65	10	-1.31	20	-0.85
99	0	-3	0	-3	3	-1.84	5	-1.65	9	-1.33	19	-0.88
100	0	-3	0	-3	3	-1.92	5	-1.68	9	-1.33	18	-0.91
101	0	-3	0	-3	2	-2.14	4	-1.71	9	-1.33	18	-0.91
102	0	-3	0	-3	1	-2.29	4	-1.71	9	-1.33	18	-0.91

Table S13. Percentile ranks and *z*-scores per age group for the Trail Making Test, Part A (< 12 years of education).

Test score (sec)	Age (years)											
	18-29 (*n* = 38)		30-49 (*n* = 87)		50-59 (*n* = 91)		60-69 (*n* = 180)		70-79 (*n* = 226)		≥ 80 (*n* = 60)	
	PR	*z*-score	PR	*z*-score	PR	*z*-score	PR	*z*-score	PR	*z*-score	PR	*z*-score
103	0	-3	0	-3	1	-2.29	4	-1.71	9	-1.33	18	-0.91
104	0	-3	0	-3	1	-2.29	4	-1.71	9	-1.35	18	-0.91
105	0	-3	0	-3	1	-2.29	4	-1.71	8	-1.38	18	-0.91
106	0	-3	0	-3	1	-2.29	4	-1.77	8	-1.38	18	-0.91
107	0	-3	0	-3	1	-2.29	3	-1.88	8	-1.38	18	-0.91
108	0	-3	0	-3	1	-2.29	3	-1.92	8	-1.38	18	-0.91
109	0	-3	0	-3	1	-2.29	3	-1.92	8	-1.38	18	-0.91
110	0	-3	0	-3	1	-2.29	2	-2.01	8	-1.4	18	-0.91
111	0	-3	0	-3	1	-2.29	2	-2.13	8	-1.41	18	-0.91
112	0	-3	0	-3	1	-2.29	2	-2.13	8	-1.43	18	-0.91
113	0	-3	0	-3	1	-2.29	2	-2.13	7	-1.46	18	-0.91
114	0	-3	0	-3	1	-2.29	2	-2.13	7	-1.47	18	-0.94
115	0	-3	0	-3	1	-2.29	2	-2.13	7	-1.49	17	-0.97
116	0	-3	0	-3	1	-2.29	2	-2.13	6	-1.53	17	-0.97
117	0	-3	0	-3	1	-2.29	2	-2.13	6	-1.54	16	-1.01
118	0	-3	0	-3	1	-2.29	2	-2.13	6	-1.56	15	-1.04
119	0	-3	0	-3	1	-2.29	1	-2.2	6	-1.58	14	-1.08
120	0	-3	0	-3	1	-2.29	1	-2.29	6	-1.6	13	-1.12
121	0	-3	0	-3	1	-2.29	1	-2.29	5	-1.62	13	-1.12

Table S13. Percentile ranks and *z*-scores per age group for the Trail Making Test, Part A (< 12 years of education).

Test score (sec)	Age (years)											
	18-29 (*n* = 38)		30-49 (*n* = 87)		50-59 (*n* = 91)		60-69 (*n* = 180)		70-79 (*n* = 226)		≥ 80 (*n* = 60)	
	PR	*z*-score	PR	*z*-score	PR	*z*-score	PR	*z*-score	PR	*z*-score	PR	*z*-score
122	0	-3	0	-3	1	-2.29	1	-2.29	5	-1.66	13	-1.12
123	0	-3	0	-3	1	-2.29	1	-2.29	4	-1.71	13	-1.12
124	0	-3	0	-3	1	-2.54	1	-2.29	4	-1.71	13	-1.12
125	0	-3	0	-3	0	-3	1	-2.29	4	-1.71	13	-1.12
126	0	-3	0	-3	0	-3	1	-2.29	4	-1.71	13	-1.12
127	0	-3	0	-3	0	-3	1	-2.29	4	-1.71	13	-1.12
128	0	-3	0	-3	0	-3	1	-2.4	4	-1.71	12	-1.2
129	0	-3	0	-3	0	-3	1	-2.54	4	-1.76	10	-1.29
130	0	-3	0	-3	0	-3	1	-2.54	4	-1.81	10	-1.29
131	0	-3	0	-3	0	-3	1	-2.54	4	-1.81	10	-1.29
132	0	-3	0	-3	0	-3	1	-2.54	4	-1.81	9	-1.34
133	0	-3	0	-3	0	-3	1	-2.54	4	-1.81	8	-1.39
134	0	-3	0	-3	0	-3	1	-2.54	4	-1.81	8	-1.39
135	0	-3	0	-3	0	-3	1	-2.54	4	-1.81	7	-1.51
136	0	-3	0	-3	0	-3	1	-2.54	4	-1.81	5	-1.65
137	0	-3	0	-3	0	-3	1	-2.54	3	-1.84	5	-1.65
138	0	-3	0	-3	0	-3	1	-2.54	3	-1.87	5	-1.65
139	0	-3	0	-3	0	-3	1	-2.54	3	-1.87	5	-1.65
140	0	-3	0	-3	0	-3	1	-2.54	3	-1.87	5	-1.65

Table S13. Percentile ranks and *z*-scores per age group for the Trail Making Test, Part A (< 12 years of education).

Test score (sec)	Age (years)											
	18-29 (*n* = 38)		30-49 (*n* = 87)		50-59 (*n* = 91)		60-69 (*n* = 180)		70-79 (*n* = 226)		≥ 80 (*n* = 60)	
	PR	*z*-score	PR	*z*-score	PR	*z*-score	PR	*z*-score	PR	*z*-score	PR	*z*-score
141	0	-3	0	-3	0	-3	1	-2.54	3	-1.87	5	-1.65
142	0	-3	0	-3	0	-3	1	-2.54	3	-1.87	5	-1.65
143	0	-3	0	-3	0	-3	1	-2.54	3	-1.87	5	-1.65
144	0	-3	0	-3	0	-3	1	-2.54	3	-1.87	5	-1.65
145	0	-3	0	-3	0	-3	1	-2.54	3	-1.87	5	-1.65
146	0	-3	0	-3	0	-3	1	-2.54	3	-1.87	4	-1.74
147	0	-3	0	-3	0	-3	1	-2.54	3	-1.9	3	-1.84
148	0	-3	0	-3	0	-3	1	-2.54	3	-1.94	3	-1.84
149	0	-3	0	-3	0	-3	1	-2.54	2	-1.98	3	-1.84
150	0	-3	0	-3	0	-3	1	-2.54	2	-2.06	3	-1.84
151	0	-3	0	-3	0	-3	1	-2.54	2	-2.11	3	-1.84
152	0	-3	0	-3	0	-3	1	-2.54	2	-2.11	3	-1.84
153	0	-3	0	-3	0	-3	1	-2.54	2	-2.11	3	-1.96
154	0	-3	0	-3	0	-3	1	-2.54	2	-2.11	2	-2.13
155	0	-3	0	-3	0	-3	1	-2.54	2	-2.11	2	-2.13
156	0	-3	0	-3	0	-3	1	-2.54	2	-2.16	2	-2.13
157	0	-3	0	-3	0	-3	1	-2.54	1	-2.22	2	-2.13
158	0	-3	0	-3	0	-3	1	-2.54	1	-2.22	2	-2.13
159	0	-3	0	-3	0	-3	1	-2.54	1	-2.29	2	-2.13

Table S13. Percentile ranks and *z*-scores per age group for the Trail Making Test, Part A (< 12 years of education).

Test score (sec)	Age (years)											
	18-29 (*n* = 38)		30-49 (*n* = 87)		50-59 (*n* = 91)		60-69 (*n* = 180)		70-79 (*n* = 226)		≥ 80 (*n* = 60)	
	PR	*z*-score	PR	*z*-score	PR	*z*-score	PR	*z*-score	PR	*z*-score	PR	*z*-score
160	0	-3	0	-3	0	-3	1	-2.54	1	-2.48	2	-2.13
161	0	-3	0	-3	0	-3	1	-2.54	0	-2.62	2	-2.13
162	0	-3	0	-3	0	-3	1	-2.54	0	-2.62	2	-2.13
163	0	-3	0	-3	0	-3	1	-2.54	0	-2.62	2	-2.13
164	0	-3	0	-3	0	-3	1	-2.54	0	-2.62	2	-2.13
165	0	-3	0	-3	0	-3	1	-2.54	0	-2.62	2	-2.13
166	0	-3	0	-3	0	-3	1	-2.54	0	-2.62	2	-2.13
167	0	-3	0	-3	0	-3	1	-2.54	0	-2.62	2	-2.13
168	0	-3	0	-3	0	-3	0	-2.77	0	-2.62	2	-2.13
169	0	-3	0	-3	0	-3	0	-3	0	-2.85	2	-2.13
170	0	-3	0	-3	0	-3	0	-3	0	-3	2	-2.13
171	0	-3	0	-3	0	-3	0	-3	0	-3	2	-2.13
172	0	-3	0	-3	0	-3	0	-3	0	-3	2	-2.13
173	0	-3	0	-3	0	-3	0	-3	0	-3	1	-2.4
≥ 174	0	-3	0	-3	0	-3	0	-3	0	-3	0	-3

White cells correspond to cognitive impairment. cells highlighted in gray represent normal cognition; **PR** Percentile rank.

Table S14. Percentile ranks and *z*-scores per age group for the Trail Making Test, Part A (≥ 12 years of education).

Test score (sec)	Age (years)											
	18-29 (*n* = 64)		30-49 (*n* = 133)		50-59 (*n* = 118)		60-69 (*n* = 166)		70-79 (*n* = 171)		≥ 80 (*n* = 53)	
	PR	*z*-score	PR	*z*-score	PR	*z*-score	PR	*z*-score	PR	*z*-score	PR	*z*-score
≤ 12	100	3	100	3	100	3	100	3	100	3	100	3
13	99	2.42	100	3	100	3	100	3	100	3	100	3
14	97	1.86	100	3	100	3	100	3	100	3	100	3
15	91	1.36	99	2.28	100	3	100	3	100	3	100	3
16	85	1.04	97	1.88	100	3	100	3	100	3	100	3
17	83	0.94	94	1.55	100	2.63	100	3	100	3	100	3
18	82	0.91	91	1.36	99	2.38	100	3	100	3	100	3
19	80	0.85	89	1.25	99	2.38	100	2.75	100	3	100	3
20	77	0.72	86	1.1	99	2.38	99	2.25	100	3	100	3
21	72	0.57	84	1	98	2.12	98	2.09	100	3	100	3
22	69	0.48	81	0.87	97	1.88	98	2.09	100	2.76	99	2.35
23	65	0.38	76	0.71	96	1.77	97	1.83	99	2.37	98	2.07
24	61	0.27	73	0.59	95	1.63	95	1.6	99	2.18	98	2.07
25	58	0.19	69	0.5	93	1.46	93	1.5	98	2.04	97	1.9
26	55	0.11	66	0.4	89	1.2	92	1.43	97	1.85	96	1.77
27	52	0.03	60	0.25	84	0.99	91	1.33	95	1.67	95	1.67
28	50	0	54	0.1	81	0.87	89	1.21	94	1.59	93	1.5
29	48	-0.04	50	-0.01	77	0.74	86	1.08	93	1.49	92	1.43
30	45	-0.12	46	-0.11	72	0.59	83	0.97	92	1.41	92	1.43

Table S14. Percentile ranks and *z*-scores per age group for the Trail Making Test, Part A (≥ 12 years of education).

Test score (sec)	Age (years)											
	18-29 (*n* = 64)		30-49 (*n* = 133)		50-59 (*n* = 118)		60-69 (*n* = 166)		70-79 (*n* = 171)		≥ 80 (*n* = 53)	
	PR	*z*-score	PR	*z*-score	PR	*z*-score	PR	*z*-score	PR	*z*-score	PR	*z*-score
31	42	-0.2	44	-0.16	69	0.5	81	0.89	92	1.39	92	1.43
32	38	-0.32	42	-0.21	67	0.42	80	0.83	91	1.33	92	1.37
33	32	-0.47	39	-0.27	62	0.3	79	0.8	90	1.28	91	1.31
34	28	-0.58	37	-0.34	58	0.19	78	0.76	89	1.23	90	1.26
35	26	-0.65	34	-0.42	52	0.05	74	0.63	87	1.14	89	1.2
36	25	-0.68	32	-0.48	48	-0.06	69	0.49	85	1.05	87	1.11
37	23	-0.73	29	-0.56	46	-0.1	65	0.39	83	0.96	84	0.99
38	20	-0.86	26	-0.65	44	-0.16	61	0.29	82	0.89	83	0.95
39	15	-1.05	23	-0.75	41	-0.23	58	0.19	80	0.82	80	0.84
40	12	-1.19	21	-0.8	39	-0.28	54	0.09	78	0.77	76	0.72
41	11	-1.23	20	-0.85	38	-0.32	51	0.02	77	0.72	75	0.68
42	11	-1.23	18	-0.92	37	-0.34	48	-0.04	75	0.66	75	0.68
43	11	-1.23	17	-0.98	35	-0.39	47	-0.08	74	0.64	74	0.63
44	10	-1.28	15	-1.06	32	-0.48	46	-0.1	73	0.59	71	0.54
45	9	-1.37	13	-1.14	30	-0.54	43	-0.17	69	0.49	70	0.51
46	8	-1.42	12	-1.2	29	-0.56	41	-0.23	66	0.4	70	0.51
47	8	-1.42	11	-1.24	27	-0.61	40	-0.26	63	0.33	69	0.49
48	8	-1.42	9	-1.32	26	-0.64	38	-0.32	60	0.24	68	0.46
49	8	-1.42	8	-1.42	25	-0.67	37	-0.34	56	0.16	68	0.46

Table S14. Percentile ranks and *z*-scores per age group for the Trail Making Test, Part A (≥ 12 years of education).

Test score (sec)	Age (years)											
	18-29 (*n* = 64)		30-49 (*n* = 133)		50-59 (*n* = 118)		60-69 (*n* = 166)		70-79 (*n* = 171)		≥ 80 (*n* = 53)	
	PR	*z*-score	PR	*z*-score	PR	*z*-score	PR	*z*-score	PR	*z*-score	PR	*z*-score
50	8	-1.42	8	-1.44	23	-0.73	35	-0.38	54	0.1	66	0.41
51	8	-1.42	8	-1.44	21	-0.8	33	-0.43	52	0.04	64	0.36
52	8	-1.42	7	-1.5	19	-0.9	33	-0.46	50	0	62	0.31
53	8	-1.42	6	-1.56	17	-0.98	32	-0.48	49	-0.03	58	0.21
54	8	-1.42	6	-1.59	16	-1.01	31	-0.49	48	-0.06	55	0.11
55	8	-1.42	5	-1.62	15	-1.05	30	-0.53	46	-0.12	52	0.04
56	8	-1.42	5	-1.66	14	-1.09	28	-0.58	44	-0.15	51	0.02
57	8	-1.42	5	-1.7	13	-1.14	27	-0.62	42	-0.2	50	0
58	8	-1.42	4	-1.74	11	-1.21	25	-0.69	40	-0.25	47	-0.08
59	8	-1.42	4	-1.78	11	-1.25	23	-0.75	39	-0.28	45	-0.12
60	8	-1.42	3	-1.83	10	-1.3	22	-0.79	38	-0.31	44	-0.15
61	8	-1.42	3	-1.88	9	-1.33	21	-0.82	37	-0.33	43	-0.17
62	8	-1.42	3	-1.88	9	-1.35	20	-0.84	36	-0.36	43	-0.17
63	8	-1.42	3	-1.88	8	-1.38	19	-0.88	35	-0.4	43	-0.17
64	6	-1.54	3	-1.88	8	-1.38	18	-0.93	34	-0.43	43	-0.17
65	3	-1.87	3	-1.94	8	-1.38	16	-1	32	-0.46	43	-0.17
66	2	-2.16	2	-2.01	7	-1.47	14	-1.08	30	-0.52	43	-0.17
67	2	-2.16	2	-2.01	6	-1.57	13	-1.13	29	-0.57	42	-0.2
68	2	-2.16	2	-2.08	6	-1.57	12	-1.19	27	-0.61	41	-0.24

Table S14. Percentile ranks and *z*-scores per age group for the Trail Making Test, Part A (≥ 12 years of education).

Test score (sec)	Age (years)											
	18-29 (*n* = 64)		30-49 (*n* = 133)		50-59 (*n* = 118)		60-69 (*n* = 166)		70-79 (*n* = 171)		≥ 80 (*n* = 53)	
	PR	*z*-score	PR	*z*-score	PR	*z*-score	PR	*z*-score	PR	*z*-score	PR	*z*-score
69	2	-2.16	2	-2.17	6	-1.57	11	-1.26	26	-0.65	40	-0.27
70	2	-2.16	2	-2.17	6	-1.6	9	-1.32	25	-0.67	39	-0.29
71	1	-2.42	2	-2.17	5	-1.68	9	-1.34	24	-0.7	38	-0.32
72	0	-3	2	-2.17	4	-1.73	9	-1.34	24	-0.72	38	-0.32
73	0	-3	1	-2.28	4	-1.73	9	-1.36	23	-0.75	37	-0.34
74	0	-3	1	-2.43	4	-1.73	8	-1.38	22	-0.78	36	-0.37
75	0	-3	1	-2.43	4	-1.73	8	-1.4	21	-0.81	35	-0.39
76	0	-3	1	-2.43	4	-1.73	7	-1.46	20	-0.85	32	-0.47
77	0	-3	1	-2.43	4	-1.73	7	-1.51	19	-0.88	29	-0.55
78	0	-3	1	-2.43	4	-1.73	7	-1.51	19	-0.89	28	-0.58
79	0	-3	0	-2.67	4	-1.73	6	-1.53	18	-0.92	27	-0.61
80	0	-3	0	-3	4	-1.73	6	-1.58	18	-0.94	25	-0.66
81	0	-3	0	-3	4	-1.78	5	-1.61	17	-0.95	25	-0.69
82	0	-3	0	-3	3	-1.83	5	-1.64	16	-0.98	25	-0.69
83	0	-3	0	-3	3	-1.89	5	-1.7	15	-1.02	25	-0.69
84	0	-3	0	-3	3	-1.96	4	-1.73	15	-1.05	25	-0.69
85	0	-3	0	-3	3	-1.96	4	-1.73	15	-1.06	25	-0.69
86	0	-3	0	-3	2	-2.03	4	-1.73	14	-1.07	25	-0.69
87	0	-3	0	-3	2	-2.13	4	-1.73	13	-1.11	23	-0.76

Table S14. Percentile ranks and *z*-scores per age group for the Trail Making Test, Part A (≥ 12 years of education).

Test score (sec)	Age (years)											
	18-29 (*n* = 64)		30-49 (*n* = 133)		50-59 (*n* = 118)		60-69 (*n* = 166)		70-79 (*n* = 171)		≥ 80 (*n* = 53)	
	PR	*z*-score	PR	*z*-score	PR	*z*-score	PR	*z*-score	PR	*z*-score	PR	*z*-score
88	0	-3	0	-3	2	-2.13	4	-1.76	13	-1.14	21	-0.82
89	0	-3	0	-3	1	-2.24	4	-1.8	13	-1.14	21	-0.82
90	0	-3	0	-3	1	-2.39	3	-1.84	12	-1.17	21	-0.82
91	0	-3	0	-3	1	-2.39	3	-1.88	11	-1.21	21	-0.82
92	0	-3	0	-3	1	-2.39	3	-1.88	11	-1.24	21	-0.82
93	0	-3	0	-3	1	-2.39	3	-1.88	11	-1.26	21	-0.82
94	0	-3	0	-3	1	-2.39	3	-1.88	10	-1.29	20	-0.85
95	0	-3	0	-3	1	-2.39	3	-1.88	9	-1.32	18	-0.92
96	0	-3	0	-3	1	-2.39	3	-1.88	9	-1.34	16	-1
97	0	-3	0	-3	1	-2.39	3	-1.88	9	-1.36	15	-1.04
98	0	-3	0	-3	1	-2.39	3	-1.88	8	-1.38	15	-1.04
99	0	-3	0	-3	1	-2.39	3	-1.88	8	-1.4	15	-1.04
100	0	-3	0	-3	1	-2.39	3	-1.93	8	-1.4	15	-1.04
101	0	-3	0	-3	1	-2.39	2	-1.98	8	-1.4	15	-1.04
102	0	-3	0	-3	1	-2.39	2	-1.98	8	-1.4	15	-1.04
103	0	-3	0	-3	1	-2.39	2	-1.98	8	-1.42	15	-1.04
104	0	-3	0	-3	1	-2.39	2	-1.98	8	-1.44	15	-1.04
105	0	-3	0	-3	1	-2.39	2	-1.98	8	-1.44	15	-1.04
106	0	-3	0	-3	1	-2.39	2	-1.98	8	-1.44	15	-1.04

Table S14. Percentile ranks and *z*-scores per age group for the Trail Making Test, Part A (≥ 12 years of education).

Test score (sec)	Age (years)											
	18-29 (*n* = 64)		30-49 (*n* = 133)		50-59 (*n* = 118)		60-69 (*n* = 166)		70-79 (*n* = 171)		≥ 80 (*n* = 53)	
	PR	*z*-score	PR	*z*-score	PR	*z*-score	PR	*z*-score	PR	*z*-score	PR	*z*-score
107	0	-3	0	-3	1	-2.39	2	-1.98	8	-1.44	15	-1.04
108	0	-3	0	-3	1	-2.39	2	-1.98	8	-1.44	14	-1.08
109	0	-3	0	-3	1	-2.39	2	-1.98	7	-1.46	13	-1.12
110	0	-3	0	-3	1	-2.39	2	-1.98	7	-1.5	12	-1.17
111	0	-3	0	-3	1	-2.39	2	-1.98	6	-1.52	11	-1.21
112	0	-3	0	-3	1	-2.39	2	-1.98	6	-1.52	11	-1.21
113	0	-3	0	-3	1	-2.39	2	-1.98	6	-1.55	11	-1.21
114	0	-3	0	-3	1	-2.39	2	-1.98	6	-1.57	11	-1.21
115	0	-3	0	-3	1	-2.39	2	-1.98	6	-1.57	11	-1.21
116	0	-3	0	-3	1	-2.39	2	-1.98	6	-1.57	11	-1.21
117	0	-3	0	-3	1	-2.39	2	-2.04	6	-1.57	11	-1.21
118	0	-3	0	-3	1	-2.39	2	-2.1	6	-1.57	11	-1.21
119	0	-3	0	-3	1	-2.39	2	-2.17	6	-1.57	11	-1.21
120	0	-3	0	-3	1	-2.39	1	-2.26	5	-1.62	10	-1.26
121	0	-3	0	-3	1	-2.39	1	-2.26	5	-1.68	9	-1.32
122	0	-3	0	-3	1	-2.39	1	-2.26	5	-1.68	9	-1.32
123	0	-3	0	-3	1	-2.39	1	-2.26	4	-1.71	9	-1.32
124	0	-3	0	-3	0	-2.64	1	-2.26	4	-1.74	9	-1.32
125	0	-3	0	-3	0	-3	1	-2.26	4	-1.78	9	-1.32

Table S14. Percentile ranks and *z*-scores per age group for the Trail Making Test, Part A (≥ 12 years of education).

Test score (sec)	Age (years)											
	18-29 (*n* = 64)		30-49 (*n* = 133)		50-59 (*n* = 118)		60-69 (*n* = 166)		70-79 (*n* = 171)		≥ 80 (*n* = 53)	
	PR	*z*-score	PR	*z*-score	PR	*z*-score	PR	*z*-score	PR	*z*-score	PR	*z*-score
126	0	-3	0	-3	0	-3	1	-2.26	4	-1.81	9	-1.32
127	0	-3	0	-3	0	-3	1	-2.26	4	-1.81	9	-1.32
128	0	-3	0	-3	0	-3	1	-2.26	4	-1.81	9	-1.32
129	0	-3	0	-3	0	-3	1	-2.26	3	-1.9	9	-1.32
130	0	-3	0	-3	0	-3	1	-2.26	2	-1.99	9	-1.32
131	0	-3	0	-3	0	-3	1	-2.26	2	-1.99	9	-1.32
132	0	-3	0	-3	0	-3	1	-2.26	2	-1.99	9	-1.32
133	0	-3	0	-3	0	-3	1	-2.26	2	-1.99	9	-1.32
134	0	-3	0	-3	0	-3	1	-2.26	2	-1.99	9	-1.32
135	0	-3	0	-3	0	-3	1	-2.26	2	-2.11	9	-1.32
136	0	-3	0	-3	0	-3	1	-2.26	1	-2.27	9	-1.32
137	0	-3	0	-3	0	-3	1	-2.26	1	-2.27	9	-1.32
138	0	-3	0	-3	0	-3	1	-2.26	1	-2.27	9	-1.32
139	0	-3	0	-3	0	-3	1	-2.26	1	-2.38	9	-1.32
140	0	-3	0	-3	0	-3	1	-2.26	1	-2.53	9	-1.32
141	0	-3	0	-3	0	-3	1	-2.37	1	-2.53	9	-1.32
142	0	-3	0	-3	0	-3	1	-2.51	1	-2.53	8	-1.38
143	0	-3	0	-3	0	-3	1	-2.51	1	-2.53	8	-1.44
144	0	-3	0	-3	0	-3	1	-2.51	1	-2.53	8	-1.44

Table S14. Percentile ranks and *z*-scores per age group for the Trail Making Test, Part A (≥ 12 years of education).

Test score (sec)	Age (years)											
	18-29 (*n* = 64)		30-49 (*n* = 133)		50-59 (*n* = 118)		60-69 (*n* = 166)		70-79 (*n* = 171)		≥ 80 (*n* = 53)	
	PR	*z*-score	PR	*z*-score	PR	*z*-score	PR	*z*-score	PR	*z*-score	PR	*z*-score
145	0	-3	0	-3	0	-3	1	-2.51	1	-2.53	8	-1.44
146	0	-3	0	-3	0	-3	1	-2.51	1	-2.53	8	-1.44
147	0	-3	0	-3	0	-3	1	-2.51	1	-2.53	8	-1.44
148	0	-3	0	-3	0	-3	1	-2.51	1	-2.53	8	-1.44
149	0	-3	0	-3	0	-3	1	-2.51	0	-2.76	8	-1.44
150	0	-3	0	-3	0	-3	1	-2.51	0	-3	8	-1.44
151	0	-3	0	-3	0	-3	1	-2.51	0	-3	8	-1.44
152	0	-3	0	-3	0	-3	1	-2.51	0	-3	7	-1.51
153	0	-3	0	-3	0	-3	0	-2.75	0	-3	6	-1.59
154	0	-3	0	-3	0	-3	0	-3	0	-3	6	-1.59
155	0	-3	0	-3	0	-3	0	-3	0	-3	5	-1.68
156	0	-3	0	-3	0	-3	0	-3	0	-3	4	-1.78
157	0	-3	0	-3	0	-3	0	-3	0	-3	4	-1.78
158	0	-3	0	-3	0	-3	0	-3	0	-3	4	-1.78
159	0	-3	0	-3	0	-3	0	-3	0	-3	4	-1.78
160	0	-3	0	-3	0	-3	0	-3	0	-3	3	-1.91
161	0	-3	0	-3	0	-3	0	-3	0	-3	2	-2.08
162	0	-3	0	-3	0	-3	0	-3	0	-3	2	-2.08
163	0	-3	0	-3	0	-3	0	-3	0	-3	2	-2.08

Table S14. Percentile ranks and *z*-scores per age group for the Trail Making Test, Part A (≥ 12 years of education).

Test score (sec)	Age (years)											
	18-29 (*n* = 64)		30-49 (*n* = 133)		50-59 (*n* = 118)		60-69 (*n* = 166)		70-79 (*n* = 171)		≥ 80 (*n* = 53)	
	PR	*z*-score	PR	*z*-score	PR	*z*-score	PR	*z*-score	PR	*z*-score	PR	*z*-score
164	0	-3	0	-3	0	-3	0	-3	0	-3	2	-2.08
165	0	-3	0	-3	0	-3	0	-3	0	-3	2	-2.08
166	0	-3	0	-3	0	-3	0	-3	0	-3	2	-2.08
167	0	-3	0	-3	0	-3	0	-3	0	-3	2	-2.08
168	0	-3	0	-3	0	-3	0	-3	0	-3	2	-2.08
169	0	-3	0	-3	0	-3	0	-3	0	-3	2	-2.08
170	0	-3	0	-3	0	-3	0	-3	0	-3	2	-2.08
171	0	-3	0	-3	0	-3	0	-3	0	-3	2	-2.08
172	0	-3	0	-3	0	-3	0	-3	0	-3	2	-2.08
173	0	-3	0	-3	0	-3	0	-3	0	-3	2	-2.08
174	0	-3	0	-3	0	-3	0	-3	0	-3	1	-2.35
≥ 175	0	-3	0	-3	0	-3	0	-3	0	-3	0	-3

White cells correspond to cognitive impairment. cells highlighted in gray represent normal cognition; **PR** Percentile rank.

Trail Making Test, Part B

Table S15. Percentile ranks and *z*-scores per age group for the Trail Making Test, Part B (< 12 years of education).

Test score (sec)	Age (years)											
	18-29 (*n* = 29)		30-49 (*n* = 73)		50-59 (*n* = 82)		60-69 (*n* = 146)		70-79 (*n* = 186)		≥ 80 (*n* = 52)	
	PR	*z*-score	PR	*z*-score	PR	*z*-score	PR	*z*-score	PR	*z*-score	PR	*z*-score
≤ 32	100	3	100	3	100	3	100	3	100	3	100	3
33	100	3	99	2.47	100	3	100	3	100	3	100	3
34	100	3	98	2.04	100	3	100	3	100	3	100	3
35	100	3	97	1.92	100	3	100	3	100	3	100	3
36	98	2.11	97	1.82	100	3	100	3	100	3	100	3
37	97	1.81	96	1.73	100	3	100	3	100	3	100	3
38	95	1.62	96	1.73	100	3	100	3	100	2.78	100	3
39	93	1.48	96	1.73	100	3	100	3	99	2.55	100	3
40	93	1.48	95	1.66	100	3	100	3	99	2.55	100	3
41	93	1.48	95	1.6	100	3	100	3	99	2.55	100	3
42	93	1.48	94	1.54	100	3	100	2.71	99	2.55	100	3
43	93	1.48	92	1.43	100	3	99	2.47	99	2.55	100	3
44	93	1.48	92	1.39	100	3	99	2.47	99	2.55	100	3
45	93	1.48	90	1.3	100	3	99	2.47	99	2.55	100	3
46	90	1.26	89	1.22	99	2.5	99	2.47	99	2.55	100	3
47	86	1.09	88	1.19	98	2.09	99	2.47	99	2.55	100	3
48	86	1.09	87	1.12	96	1.79	99	2.47	99	2.4	100	3

Table S15. Percentile ranks and *z*-scores per age group for the Trail Making Test, Part B (< 12 years of education).

Test score (sec)	Age (years)											
	18-29 (*n* = 29)		30-49 (*n* = 73)		50-59 (*n* = 82)		60-69 (*n* = 146)		70-79 (*n* = 186)		≥ 80 (*n* = 52)	
	PR	*z*-score	PR	*z*-score	PR	*z*-score	PR	*z*-score	PR	*z*-score	PR	*z*-score
49	86	1.09	86	1.06	95	1.65	99	2.47	99	2.29	100	3
50	84	1.01	84	1	95	1.65	99	2.47	99	2.29	100	3
51	83	0.94	82	0.92	95	1.59	99	2.47	99	2.29	100	3
52	81	0.87	80	0.84	94	1.54	99	2.31	99	2.29	100	3
53	78	0.75	79	0.79	93	1.49	99	2.2	99	2.21	100	3
54	76	0.7	77	0.72	92	1.41	98	2.04	98	2.14	100	3
55	74	0.64	75	0.68	91	1.33	97	1.86	98	2.08	100	3
56	72	0.59	75	0.66	90	1.26	97	1.82	98	2.02	100	3
57	72	0.59	71	0.56	88	1.19	97	1.82	98	2.02	100	3
58	71	0.54	68	0.46	88	1.16	97	1.82	98	2.02	100	3
59	69	0.49	67	0.44	87	1.1	97	1.82	98	2.02	100	3
60	69	0.49	66	0.4	85	1.05	97	1.82	98	2.02	100	3
61	69	0.49	64	0.35	85	1.02	97	1.82	98	2.02	100	3
62	69	0.49	63	0.33	84	1	97	1.82	98	2.02	99	2.34
63	66	0.39	63	0.33	84	0.97	96	1.77	98	2.02	98	2.07
64	62	0.3	63	0.33	82	0.92	95	1.63	98	2.02	98	2.07
65	62	0.3	62	0.31	82	0.9	94	1.54	98	2.02	98	2.07
66	60	0.26	61	0.27	81	0.88	94	1.54	98	2.02	98	2.07
67	57	0.17	60	0.26	80	0.85	94	1.54	98	2.02	98	2.07

Table S15. Percentile ranks and *z*-scores per age group for the Trail Making Test, Part B (< 12 years of education).

Test score (sec)	Age (years)											
	18-29 (*n* = 29)		30-49 (*n* = 73)		50-59 (*n* = 82)		60-69 (*n* = 146)		70-79 (*n* = 186)		≥ 80 (*n* = 52)	
	PR	*z*-score	PR	*z*-score	PR	*z*-score	PR	*z*-score	PR	*z*-score	PR	*z*-score
68	53	0.08	58	0.2	80	0.85	94	1.54	98	2.02	98	2.07
69	52	0.04	56	0.15	80	0.85	94	1.54	98	1.97	98	2.07
70	50	0	55	0.13	80	0.83	93	1.46	97	1.88	98	2.07
71	45	-0.13	55	0.12	78	0.77	91	1.36	97	1.84	98	2.07
72	40	-0.27	53	0.08	75	0.67	90	1.28	97	1.84	98	2.07
73	38	-0.31	52	0.05	73	0.59	89	1.21	97	1.84	98	2.07
74	38	-0.31	52	0.05	71	0.56	88	1.19	96	1.77	98	2.07
75	38	-0.31	51	0.01	71	0.54	87	1.1	96	1.71	98	2.07
76	38	-0.31	48	-0.06	70	0.51	84	1	95	1.68	98	2.07
77	38	-0.31	46	-0.11	68	0.45	83	0.96	95	1.63	98	2.07
78	34	-0.4	45	-0.13	67	0.44	82	0.92	95	1.6	97	1.89
79	31	-0.5	45	-0.14	67	0.44	81	0.88	95	1.6	96	1.76
80	31	-0.5	43	-0.18	66	0.42	80	0.85	94	1.54	96	1.76
81	31	-0.5	42	-0.19	66	0.4	80	0.84	93	1.47	95	1.66
82	31	-0.5	42	-0.19	66	0.4	80	0.83	93	1.45	94	1.57
83	31	-0.5	42	-0.21	65	0.39	79	0.79	92	1.41	94	1.57
84	31	-0.5	41	-0.23	64	0.35	78	0.77	92	1.38	94	1.57
85	29	-0.55	41	-0.23	63	0.32	78	0.76	91	1.34	94	1.57
86	28	-0.6	40	-0.25	62	0.29	77	0.75	91	1.33	94	1.57

Table S15. Percentile ranks and *z*-scores per age group for the Trail Making Test, Part B (< 12 years of education).

Test score (sec)	Age (years)											
	18-29 (*n* = 29)		30-49 (*n* = 73)		50-59 (*n* = 82)		60-69 (*n* = 146)		70-79 (*n* = 186)		≥ 80 (*n* = 52)	
	PR	*z*-score	PR	*z*-score	PR	*z*-score	PR	*z*-score	PR	*z*-score	PR	*z*-score
87	28	-0.6	39	-0.28	61	0.27	77	0.72	91	1.31	94	1.57
88	28	-0.6	38	-0.3	60	0.24	76	0.69	90	1.3	94	1.57
89	28	-0.6	38	-0.3	58	0.2	75	0.68	90	1.26	94	1.57
90	28	-0.6	38	-0.3	54	0.1	75	0.67	89	1.24	94	1.57
91	28	-0.6	38	-0.3	51	0.03	75	0.66	89	1.24	93	1.49
92	28	-0.6	37	-0.34	51	0.01	75	0.66	89	1.24	91	1.36
93	28	-0.6	36	-0.37	49	-0.02	74	0.65	89	1.21	90	1.3
94	28	-0.6	36	-0.37	49	-0.04	73	0.61	88	1.15	90	1.3
95	28	-0.6	36	-0.37	49	-0.04	71	0.55	87	1.11	90	1.3
96	28	-0.6	36	-0.37	48	-0.05	70	0.51	87	1.1	90	1.3
97	28	-0.6	35	-0.39	47	-0.08	68	0.48	86	1.08	90	1.3
98	28	-0.6	33	-0.45	45	-0.13	67	0.45	85	1.05	90	1.3
99	28	-0.6	31	-0.51	42	-0.2	67	0.44	85	1.05	90	1.3
100	28	-0.6	29	-0.55	38	-0.3	65	0.38	85	1.05	90	1.3
101	28	-0.6	29	-0.56	37	-0.35	63	0.32	85	1.04	90	1.3
102	28	-0.6	29	-0.56	37	-0.35	62	0.3	85	1.03	90	1.3
103	28	-0.6	29	-0.56	36	-0.36	62	0.29	84	1.01	90	1.3
104	26	-0.65	28	-0.59	35	-0.4	62	0.29	84	0.97	90	1.3
105	21	-0.82	27	-0.61	32	-0.46	61	0.28	83	0.94	90	1.3

Table S15. Percentile ranks and *z*-scores per age group for the Trail Making Test, Part B (< 12 years of education).

Test score (sec)	Age (years)											
	18-29 (*n* = 29)		30-49 (*n* = 73)		50-59 (*n* = 82)		60-69 (*n* = 146)		70-79 (*n* = 186)		≥ 80 (*n* = 52)	
	PR	*z*-score	PR	*z*-score	PR	*z*-score	PR	*z*-score	PR	*z*-score	PR	*z*-score
106	16	-1.02	27	-0.61	30	-0.52	61	0.26	82	0.91	90	1.3
107	14	-1.09	27	-0.61	30	-0.52	60	0.24	81	0.87	90	1.3
108	12	-1.18	27	-0.61	30	-0.52	59	0.21	80	0.83	89	1.24
109	10	-1.27	27	-0.63	30	-0.52	57	0.18	79	0.81	88	1.15
110	10	-1.27	26	-0.65	30	-0.52	56	0.14	79	0.79	87	1.1
111	10	-1.27	26	-0.65	29	-0.55	55	0.12	78	0.78	87	1.1
112	10	-1.27	25	-0.67	28	-0.59	55	0.12	78	0.78	87	1.1
113	9	-1.37	25	-0.69	28	-0.59	55	0.12	78	0.76	87	1.1
114	7	-1.49	23	-0.73	28	-0.59	54	0.09	77	0.74	87	1.1
115	7	-1.49	21	-0.8	28	-0.59	52	0.05	76	0.7	87	1.1
116	7	-1.49	20	-0.85	28	-0.59	51	0.02	74	0.64	87	1.1
117	7	-1.49	19	-0.88	27	-0.6	50	0	73	0.61	87	1.1
118	5	-1.63	18	-0.9	26	-0.64	50	-0.01	73	0.61	87	1.1
119	3	-1.82	18	-0.93	25	-0.68	49	-0.03	72	0.58	87	1.1
120	2	-2.12	16	-0.98	24	-0.72	49	-0.04	70	0.52	86	1.06
121	0	-3	15	-1.04	23	-0.76	48	-0.06	69	0.48	85	1.02
122	0	-3	15	-1.04	22	-0.78	47	-0.07	68	0.46	85	1.02
123	0	-3	15	-1.04	22	-0.78	47	-0.07	67	0.43	85	1.02
124	0	-3	15	-1.04	21	-0.8	47	-0.09	67	0.43	85	1.02

Table S15. Percentile ranks and *z*-scores per age group for the Trail Making Test, Part B (< 12 years of education).

Test score (sec)	Age (years)											
	18-29 (*n* = 29)		30-49 (*n* = 73)		50-59 (*n* = 82)		60-69 (*n* = 146)		70-79 (*n* = 186)		≥ 80 (*n* = 52)	
	PR	*z*-score	PR	*z*-score	PR	*z*-score	PR	*z*-score	PR	*z*-score	PR	*z*-score
125	0	-3	15	-1.04	21	-0.82	46	-0.12	67	0.43	85	1.02
126	0	-3	15	-1.04	20	-0.84	45	-0.13	66	0.42	85	1.02
127	0	-3	15	-1.04	19	-0.89	45	-0.14	66	0.4	85	1.02
128	0	-3	15	-1.04	18	-0.91	44	-0.15	65	0.37	85	1.02
129	0	-3	14	-1.07	18	-0.91	44	-0.16	64	0.35	85	1.02
130	0	-3	14	-1.1	18	-0.91	44	-0.16	63	0.34	84	0.98
131	0	-3	14	-1.1	18	-0.91	43	-0.17	63	0.33	82	0.9
132	0	-3	13	-1.13	18	-0.93	43	-0.19	63	0.32	81	0.86
133	0	-3	12	-1.2	17	-0.96	42	-0.19	62	0.3	81	0.86
134	0	-3	10	-1.27	17	-0.96	42	-0.19	62	0.3	81	0.86
135	0	-3	10	-1.31	17	-0.96	42	-0.19	62	0.29	81	0.86
136	0	-3	10	-1.31	17	-0.96	42	-0.21	61	0.27	81	0.86
137	0	-3	10	-1.31	17	-0.96	41	-0.23	60	0.25	81	0.86
138	0	-3	10	-1.31	17	-0.96	41	-0.23	60	0.25	81	0.86
139	0	-3	10	-1.31	17	-0.96	40	-0.25	60	0.24	81	0.86
140	0	-3	9	-1.35	17	-0.96	40	-0.27	59	0.23	80	0.83
141	0	-3	8	-1.4	17	-0.96	40	-0.27	59	0.23	79	0.8
142	0	-3	8	-1.4	16	-0.98	40	-0.27	58	0.2	78	0.76
143	0	-3	8	-1.4	16	-1.01	39	-0.27	57	0.17	77	0.73

Table S15. Percentile ranks and *z*-scores per age group for the Trail Making Test, Part B (< 12 years of education).

Test score (sec)	Age (years)											
	18-29 (*n* = 29)		30-49 (*n* = 73)		50-59 (*n* = 82)		60-69 (*n* = 146)		70-79 (*n* = 186)		≥ 80 (*n* = 52)	
	PR	*z*-score	PR	*z*-score	PR	*z*-score	PR	*z*-score	PR	*z*-score	PR	*z*-score
144	0	-3	8	-1.4	16	-1.01	39	-0.29	57	0.17	77	0.73
145	0	-3	8	-1.4	16	-1.01	38	-0.32	57	0.17	77	0.73
146	0	-3	8	-1.4	16	-1.01	37	-0.34	57	0.16	76	0.7
147	0	-3	8	-1.4	16	-1.01	37	-0.35	56	0.16	74	0.64
148	0	-3	8	-1.4	16	-1.01	36	-0.36	56	0.15	73	0.61
149	0	-3	8	-1.4	16	-1.01	36	-0.36	56	0.14	73	0.61
150	0	-3	8	-1.4	16	-1.01	36	-0.37	54	0.1	73	0.61
151	0	-3	8	-1.4	16	-1.01	35	-0.39	52	0.05	72	0.58
152	0	-3	8	-1.44	16	-1.01	35	-0.4	51	0.02	71	0.55
153	0	-3	7	-1.49	15	-1.03	34	-0.41	49	-0.03	71	0.55
154	0	-3	7	-1.49	14	-1.08	34	-0.42	48	-0.05	69	0.5
155	0	-3	6	-1.55	13	-1.14	33	-0.45	48	-0.07	67	0.44
156	0	-3	5	-1.6	12	-1.17	32	-0.47	47	-0.09	67	0.44
157	0	-3	5	-1.6	12	-1.17	32	-0.47	46	-0.1	67	0.44
158	0	-3	5	-1.67	12	-1.2	32	-0.47	45	-0.13	67	0.44
159	0	-3	4	-1.74	11	-1.23	32	-0.49	44	-0.15	67	0.44
160	0	-3	4	-1.74	10	-1.27	30	-0.52	43	-0.17	67	0.44
161	0	-3	4	-1.74	10	-1.3	30	-0.53	43	-0.18	67	0.44
162	0	-3	4	-1.74	10	-1.3	30	-0.54	43	-0.18	66	0.42

Table S15. Percentile ranks and *z*-scores per age group for the Trail Making Test, Part B (< 12 years of education).

Test score (sec)	Age (years)											
	18-29 (*n* = 29)		30-49 (*n* = 73)		50-59 (*n* = 82)		60-69 (*n* = 146)		70-79 (*n* = 186)		≥ 80 (*n* = 52)	
	PR	*z*-score	PR	*z*-score	PR	*z*-score	PR	*z*-score	PR	*z*-score	PR	*z*-score
163	0	-3	4	-1.74	10	-1.3	29	-0.56	43	-0.19	65	0.39
164	0	-3	3	-1.83	10	-1.3	28	-0.58	42	-0.19	65	0.39
165	0	-3	3	-1.92	10	-1.3	28	-0.59	42	-0.2	65	0.39
166	0	-3	3	-1.92	10	-1.3	28	-0.59	42	-0.22	65	0.39
167	0	-3	3	-1.92	10	-1.3	28	-0.59	41	-0.24	65	0.39
168	0	-3	3	-1.92	10	-1.3	28	-0.6	40	-0.26	65	0.39
169	0	-3	2	-2.05	10	-1.3	27	-0.62	40	-0.26	65	0.39
170	0	-3	1	-2.21	10	-1.3	27	-0.63	40	-0.26	64	0.36
171	0	-3	1	-2.21	10	-1.3	27	-0.63	40	-0.26	63	0.34
172	0	-3	1	-2.21	10	-1.3	26	-0.64	40	-0.26	63	0.34
173	0	-3	1	-2.21	9	-1.34	26	-0.65	40	-0.26	63	0.34
174	0	-3	1	-2.21	9	-1.37	26	-0.65	40	-0.27	63	0.34
175	0	-3	1	-2.21	8	-1.41	26	-0.65	39	-0.28	63	0.31
176	0	-3	1	-2.21	7	-1.46	26	-0.66	38	-0.3	61	0.26
177	0	-3	1	-2.21	7	-1.46	25	-0.67	38	-0.31	60	0.24
178	0	-3	1	-2.21	7	-1.46	25	-0.67	37	-0.33	60	0.24
179	0	-3	1	-2.21	7	-1.46	25	-0.67	37	-0.34	59	0.21
180	0	-3	1	-2.21	7	-1.5	24	-0.71	35	-0.39	57	0.16
181	0	-3	1	-2.21	6	-1.55	23	-0.76	33	-0.44	56	0.14

Table S15. Percentile ranks and *z*-scores per age group for the Trail Making Test, Part B (< 12 years of education).

Test score (sec)	Age (years)											
	18-29 (*n* = 29)		30-49 (*n* = 73)		50-59 (*n* = 82)		60-69 (*n* = 146)		70-79 (*n* = 186)		≥ 80 (*n* = 52)	
	PR	*z*-score	PR	*z*-score	PR	*z*-score	PR	*z*-score	PR	*z*-score	PR	*z*-score
182	0	-3	1	-2.21	6	-1.55	23	-0.76	33	-0.44	56	0.14
183	0	-3	1	-2.21	6	-1.55	22	-0.77	33	-0.44	55	0.12
184	0	-3	1	-2.21	6	-1.55	22	-0.78	33	-0.45	54	0.09
185	0	-3	1	-2.21	6	-1.55	22	-0.79	33	-0.45	53	0.07
186	0	-3	1	-2.21	6	-1.55	21	-0.8	33	-0.45	52	0.04
187	0	-3	1	-2.21	6	-1.55	21	-0.8	33	-0.45	51	0.02
188	0	-3	1	-2.21	6	-1.55	21	-0.82	32	-0.47	50	0
189	0	-3	1	-2.21	6	-1.55	21	-0.83	32	-0.48	50	0
190	0	-3	1	-2.21	6	-1.55	21	-0.83	32	-0.48	50	0
191	0	-3	1	-2.21	6	-1.55	21	-0.83	32	-0.48	50	0
192	0	-3	1	-2.21	6	-1.55	21	-0.83	31	-0.49	50	0
193	0	-3	1	-2.21	6	-1.55	21	-0.83	31	-0.5	50	0
194	0	-3	1	-2.21	5	-1.6	21	-0.83	31	-0.51	50	0
195	0	-3	1	-2.21	5	-1.66	20	-0.85	30	-0.53	48	-0.05
196	0	-3	1	-2.21	5	-1.66	19	-0.88	30	-0.53	46	-0.1
197	0	-3	1	-2.21	5	-1.66	19	-0.88	30	-0.53	46	-0.1
198	0	-3	1	-2.21	5	-1.66	19	-0.88	30	-0.53	46	-0.1
199	0	-3	1	-2.21	5	-1.66	19	-0.88	30	-0.54	46	-0.1
200	0	-3	1	-2.21	4	-1.8	18	-0.9	30	-0.54	45	-0.13

Table S15. Percentile ranks and *z*-scores per age group for the Trail Making Test, Part B (< 12 years of education).

Test score (sec)	Age (years)											
	18-29 (*n* = 29)		30-49 (*n* = 73)		50-59 (*n* = 82)		60-69 (*n* = 146)		70-79 (*n* = 186)		≥ 80 (*n* = 52)	
	PR	*z*-score	PR	*z*-score	PR	*z*-score	PR	*z*-score	PR	*z*-score	PR	*z*-score
201	0	-3	1	-2.21	2	-1.97	18	-0.93	29	-0.55	44	-0.15
202	0	-3	1	-2.21	2	-1.97	18	-0.93	29	-0.56	43	-0.17
203	0	-3	1	-2.21	2	-1.97	17	-0.94	29	-0.56	42	-0.2
204	0	-3	1	-2.21	2	-1.97	17	-0.97	29	-0.56	42	-0.2
205	0	-3	1	-2.21	2	-1.97	16	-0.98	29	-0.56	42	-0.2
206	0	-3	1	-2.21	2	-1.97	16	-0.98	29	-0.56	42	-0.2
207	0	-3	1	-2.21	2	-1.97	16	-0.98	29	-0.57	42	-0.2
208	0	-3	1	-2.21	2	-1.97	16	-1	28	-0.57	41	-0.22
209	0	-3	1	-2.21	2	-1.97	16	-1.01	28	-0.58	40	-0.25
210	0	-3	1	-2.47	2	-1.97	15	-1.04	27	-0.63	38	-0.3
211	0	-3	0	-3	2	-1.97	14	-1.07	25	-0.68	37	-0.35
212	0	-3	0	-3	2	-1.97	14	-1.07	24	-0.7	37	-0.35
213	0	-3	0	-3	2	-1.97	14	-1.07	24	-0.71	37	-0.35
214	0	-3	0	-3	2	-1.97	14	-1.07	23	-0.73	37	-0.35
215	0	-3	0	-3	2	-1.97	14	-1.07	23	-0.74	37	-0.35
216	0	-3	0	-3	2	-1.97	14	-1.08	23	-0.74	37	-0.35
217	0	-3	0	-3	2	-1.97	14	-1.1	23	-0.74	37	-0.35
218	0	-3	0	-3	2	-2.09	14	-1.1	23	-0.74	37	-0.35
219	0	-3	0	-3	1	-2.25	13	-1.11	23	-0.74	37	-0.35

Table S15. Percentile ranks and *z*-scores per age group for the Trail Making Test, Part B (< 12 years of education).

Test score (sec)	Age (years)											
	18-29 (*n* = 29)		30-49 (*n* = 73)		50-59 (*n* = 82)		60-69 (*n* = 146)		70-79 (*n* = 186)		≥ 80 (*n* = 52)	
	PR	*z*-score	PR	*z*-score	PR	*z*-score	PR	*z*-score	PR	*z*-score	PR	*z*-score
220	0	-3	0	-3	1	-2.25	12	-1.16	23	-0.76	37	-0.35
221	0	-3	0	-3	1	-2.25	12	-1.2	22	-0.78	37	-0.35
222	0	-3	0	-3	1	-2.25	12	-1.2	22	-0.78	37	-0.35
223	0	-3	0	-3	1	-2.25	12	-1.2	22	-0.78	37	-0.35
224	0	-3	0	-3	1	-2.25	12	-1.2	22	-0.78	37	-0.35
225	0	-3	0	-3	1	-2.25	11	-1.22	21	-0.8	37	-0.35
226	0	-3	0	-3	1	-2.25	11	-1.23	21	-0.81	37	-0.35
227	0	-3	0	-3	1	-2.25	11	-1.23	21	-0.82	37	-0.35
228	0	-3	0	-3	1	-2.25	11	-1.23	20	-0.84	37	-0.35
229	0	-3	0	-3	1	-2.25	11	-1.23	20	-0.86	37	-0.35
230	0	-3	0	-3	1	-2.25	11	-1.23	19	-0.87	37	-0.35
231	0	-3	0	-3	1	-2.25	11	-1.23	19	-0.88	37	-0.35
232	0	-3	0	-3	1	-2.25	11	-1.23	19	-0.89	37	-0.35
233	0	-3	0	-3	1	-2.25	11	-1.23	19	-0.9	37	-0.35
234	0	-3	0	-3	1	-2.25	11	-1.23	18	-0.92	37	-0.35
235	0	-3	0	-3	1	-2.25	11	-1.23	18	-0.93	37	-0.35
236	0	-3	0	-3	1	-2.25	11	-1.23	18	-0.93	37	-0.35
237	0	-3	0	-3	1	-2.25	11	-1.23	18	-0.93	37	-0.35
238	0	-3	0	-3	1	-2.25	11	-1.23	18	-0.93	37	-0.35

Table S15. Percentile ranks and *z*-scores per age group for the Trail Making Test, Part B (< 12 years of education).

Test score (sec)	Age (years)											
	18-29 (*n* = 29)		30-49 (*n* = 73)		50-59 (*n* = 82)		60-69 (*n* = 146)		70-79 (*n* = 186)		≥ 80 (*n* = 52)	
	PR	*z*-score	PR	*z*-score	PR	*z*-score	PR	*z*-score	PR	*z*-score	PR	*z*-score
239	0	-3	0	-3	1	-2.25	11	-1.23	18	-0.93	37	-0.35
240	0	-3	0	-3	1	-2.25	11	-1.23	17	-0.96	35	-0.4
241	0	-3	0	-3	1	-2.25	11	-1.23	16	-0.99	32	-0.48
242	0	-3	0	-3	1	-2.25	11	-1.23	16	-0.99	31	-0.51
243	0	-3	0	-3	1	-2.25	11	-1.25	16	-0.99	31	-0.51
244	0	-3	0	-3	1	-2.25	10	-1.27	16	-0.99	31	-0.51
245	0	-3	0	-3	1	-2.25	10	-1.27	16	-0.99	31	-0.51
246	0	-3	0	-3	1	-2.25	10	-1.27	16	-0.99	31	-0.51
247	0	-3	0	-3	1	-2.25	10	-1.27	16	-0.99	30	-0.53
248	0	-3	0	-3	1	-2.25	10	-1.27	16	-0.99	29	-0.56
249	0	-3	0	-3	1	-2.25	10	-1.27	16	-0.99	29	-0.56
250	0	-3	0	-3	1	-2.25	10	-1.27	16	-1.01	29	-0.56
251	0	-3	0	-3	1	-2.25	10	-1.27	16	-1.02	29	-0.56
252	0	-3	0	-3	1	-2.25	10	-1.27	16	-1.02	29	-0.56
253	0	-3	0	-3	1	-2.25	10	-1.27	16	-1.02	29	-0.56
254	0	-3	0	-3	1	-2.25	10	-1.27	16	-1.02	29	-0.56
255	0	-3	0	-3	1	-2.25	10	-1.27	15	-1.03	29	-0.56
256	0	-3	0	-3	1	-2.25	10	-1.27	15	-1.04	29	-0.56
257	0	-3	0	-3	1	-2.25	10	-1.27	15	-1.04	29	-0.56

Table S15. Percentile ranks and *z*-scores per age group for the Trail Making Test, Part B (< 12 years of education).

Test score (sec)	Age (years)											
	18-29 (*n* = 29)		30-49 (*n* = 73)		50-59 (*n* = 82)		60-69 (*n* = 146)		70-79 (*n* = 186)		≥ 80 (*n* = 52)	
	PR	*z*-score	PR	*z*-score	PR	*z*-score	PR	*z*-score	PR	*z*-score	PR	*z*-score
258	0	-3	0	-3	1	-2.25	10	-1.27	15	-1.04	29	-0.56
259	0	-3	0	-3	1	-2.25	10	-1.27	15	-1.04	29	-0.56
260	0	-3	0	-3	1	-2.25	10	-1.27	15	-1.05	29	-0.56
261	0	-3	0	-3	1	-2.25	10	-1.27	14	-1.07	28	-0.59
262	0	-3	0	-3	1	-2.25	10	-1.27	13	-1.12	27	-0.62
263	0	-3	0	-3	1	-2.25	10	-1.27	12	-1.17	26	-0.65
264	0	-3	0	-3	1	-2.25	10	-1.27	12	-1.19	25	-0.68
265	0	-3	0	-3	1	-2.25	10	-1.29	12	-1.19	25	-0.68
266	0	-3	0	-3	1	-2.25	10	-1.31	12	-1.19	25	-0.68
267	0	-3	0	-3	1	-2.25	10	-1.31	12	-1.19	25	-0.68
268	0	-3	0	-3	1	-2.25	10	-1.31	12	-1.19	25	-0.68
269	0	-3	0	-3	1	-2.25	10	-1.31	12	-1.19	25	-0.68
270	0	-3	0	-3	1	-2.25	9	-1.33	12	-1.19	25	-0.68
271	0	-3	0	-3	1	-2.25	9	-1.35	12	-1.19	23	-0.74
272	0	-3	0	-3	1	-2.51	9	-1.35	12	-1.2	21	-0.81
273	0	-3	0	-3	0	-3	9	-1.35	11	-1.23	21	-0.81
274	0	-3	0	-3	0	-3	9	-1.35	11	-1.24	20	-0.84
275	0	-3	0	-3	0	-3	9	-1.35	11	-1.24	19	-0.87
276	0	-3	0	-3	0	-3	9	-1.35	11	-1.24	19	-0.87

Table S15. Percentile ranks and *z*-scores per age group for the Trail Making Test, Part B (< 12 years of education).

Test score (sec)	Age (years)											
	18-29 (*n* = 29)		30-49 (*n* = 73)		50-59 (*n* = 82)		60-69 (*n* = 146)		70-79 (*n* = 186)		≥ 80 (*n* = 52)	
	PR	*z*-score	PR	*z*-score	PR	*z*-score	PR	*z*-score	PR	*z*-score	PR	*z*-score
277	0	-3	0	-3	0	-3	9	-1.35	11	-1.24	19	-0.87
278	0	-3	0	-3	0	-3	9	-1.35	11	-1.24	19	-0.87
279	0	-3	0	-3	0	-3	9	-1.37	11	-1.24	19	-0.87
280	0	-3	0	-3	0	-3	8	-1.4	11	-1.24	18	-0.91
281	0	-3	0	-3	0	-3	8	-1.42	11	-1.24	17	-0.95
282	0	-3	0	-3	0	-3	8	-1.44	11	-1.24	17	-0.95
283	0	-3	0	-3	0	-3	8	-1.44	11	-1.24	16	-0.98
284	0	-3	0	-3	0	-3	8	-1.44	11	-1.24	15	-1.03
285	0	-3	0	-3	0	-3	8	-1.44	11	-1.24	15	-1.03
286	0	-3	0	-3	0	-3	8	-1.44	11	-1.24	15	-1.03
287	0	-3	0	-3	0	-3	8	-1.44	11	-1.24	15	-1.03
288	0	-3	0	-3	0	-3	7	-1.47	11	-1.24	15	-1.03
289	0	-3	0	-3	0	-3	7	-1.49	11	-1.24	15	-1.03
290	0	-3	0	-3	0	-3	7	-1.49	11	-1.24	15	-1.03
291	0	-3	0	-3	0	-3	7	-1.49	11	-1.24	14	-1.07
292	0	-3	0	-3	0	-3	7	-1.49	11	-1.24	13	-1.11
293	0	-3	0	-3	0	-3	7	-1.49	11	-1.24	13	-1.11
294	0	-3	0	-3	0	-3	7	-1.52	11	-1.24	13	-1.11
295	0	-3	0	-3	0	-3	6	-1.55	10	-1.26	13	-1.11

Table S15. Percentile ranks and *z*-scores per age group for the Trail Making Test, Part B (< 12 years of education).

Test score (sec)	Age (years)											
	18-29 (*n* = 29)		30-49 (*n* = 73)		50-59 (*n* = 82)		60-69 (*n* = 146)		70-79 (*n* = 186)		≥ 80 (*n* = 52)	
	PR	*z*-score	PR	*z*-score	PR	*z*-score	PR	*z*-score	PR	*z*-score	PR	*z*-score
296	0	-3	0	-3	0	-3	6	-1.55	10	-1.27	13	-1.11
297	0	-3	0	-3	0	-3	6	-1.55	10	-1.27	13	-1.11
298	0	-3	0	-3	0	-3	6	-1.55	10	-1.27	13	-1.11
299	0	-3	0	-3	0	-3	6	-1.55	10	-1.27	13	-1.11
300	0	-3	0	-3	0	-3	3	-1.87	5	-1.64	7	-1.5

White cells correspond to cognitive impairment. cells highlighted in gray represent normal cognition; **PR** Percentile rank.

Table S16. Percentile ranks and *z*-scores per age group for the Trail Making Test, Part B (≥ 12 years of education).

Test score (sec)	Age (years)											
	18-29 (*n* = 59)		30-49 (*n* = 123)		50-59 (*n* = 103)		60-69 (*n* = 142)		70-79 (*n* = 140)		≥ 80 (*n* = 44)	
	PR	*z*-score	PR	*z*-score	PR	*z*-score	PR	*z*-score	PR	*z*-score	PR	*z*-score
≤ 30	100	3	100	3	100	3	100	3	100	3	100	3
31	99	2.38	100	3	100	3	100	3	100	3	100	3
32	98	2.12	100	3	100	3	100	3	100	3	100	3
33	97	1.95	100	3	100	3	100	3	100	3	100	3
34	97	1.82	100	3	100	3	100	3	100	3	100	3
35	97	1.82	100	2.64	100	3	100	3	100	3	100	3
36	97	1.82	98	2.13	100	3	100	3	100	3	100	3

Table S16. Percentile ranks and *z*-scores per age group for the Trail Making Test, Part B (≥ 12 years of education).

Test score (sec)	Age (years)											
	18-29 (*n* = 59)		30-49 (*n* = 123)		50-59 (*n* = 103)		60-69 (*n* = 142)		70-79 (*n* = 140)		≥ 80 (*n* = 44)	
	PR	*z*-score	PR	*z*-score	PR	*z*-score	PR	*z*-score	PR	*z*-score	PR	*z*-score
37	96	1.72	97	1.9	100	3	100	3	100	3	100	3
38	95	1.63	96	1.79	100	3	100	3	100	3	100	3
39	92	1.43	96	1.69	100	2.58	100	3	100	3	100	3
40	89	1.22	95	1.65	99	2.33	100	3	100	3	100	3
41	86	1.1	94	1.54	99	2.33	100	3	100	3	100	3
42	83	0.95	91	1.34	99	2.33	99	2.45	100	3	100	3
43	79	0.8	89	1.2	99	2.33	99	2.19	100	3	100	3
44	76	0.71	87	1.1	99	2.18	99	2.19	100	3	100	3
45	76	0.71	85	1.03	98	2.06	99	2.19	100	3	100	3
46	74	0.63	84	1	98	1.97	99	2.19	100	3	100	3
47	69	0.5	83	0.93	97	1.82	98	2.1	100	2.69	99	2.27
48	66	0.41	80	0.85	96	1.76	98	2.03	99	2.45	98	2
49	64	0.34	78	0.78	96	1.7	98	2.03	99	2.3	98	2
50	61	0.27	76	0.72	94	1.52	98	1.96	99	2.19	98	2
51	57	0.17	75	0.68	92	1.42	97	1.9	99	2.19	98	2
52	53	0.08	74	0.63	91	1.35	97	1.85	99	2.19	98	2
53	50	0	71	0.54	90	1.27	96	1.72	99	2.19	98	2
54	46	-0.11	67	0.45	89	1.21	95	1.65	98	2.1	98	2
55	44	-0.15	64	0.36	87	1.14	95	1.65	98	2.02	98	2

Table S16. Percentile ranks and *z*-scores per age group for the Trail Making Test, Part B (≥ 12 years of education).

Test score (sec)	Age (years)											
	18-29 (*n* = 59)		30-49 (*n* = 123)		50-59 (*n* = 103)		60-69 (*n* = 142)		70-79 (*n* = 140)		≥ 80 (*n* = 44)	
	PR	*z*-score	PR	*z*-score	PR	*z*-score	PR	*z*-score	PR	*z*-score	PR	*z*-score
56	44	-0.15	62	0.31	85	1.03	94	1.55	98	2.02	98	2
57	44	-0.15	61	0.26	83	0.97	92	1.42	97	1.9	98	2
58	44	-0.15	59	0.21	83	0.95	91	1.33	96	1.75	98	2
59	44	-0.15	57	0.16	82	0.91	90	1.28	96	1.71	98	2
60	44	-0.15	53	0.08	81	0.88	90	1.26	95	1.68	97	1.82
61	42	-0.2	51	0.02	81	0.86	88	1.19	95	1.64	94	1.58
62	41	-0.24	50	-0.02	80	0.84	87	1.12	95	1.61	93	1.48
63	41	-0.24	48	-0.06	79	0.81	86	1.07	94	1.58	93	1.48
64	39	-0.28	47	-0.08	78	0.77	85	1.03	94	1.58	91	1.33
65	37	-0.33	46	-0.1	76	0.71	85	1.01	94	1.58	89	1.2
66	36	-0.37	45	-0.14	74	0.65	85	1.01	94	1.54	89	1.2
67	33	-0.44	43	-0.18	72	0.57	83	0.95	93	1.49	89	1.2
68	32	-0.47	41	-0.24	69	0.49	82	0.9	93	1.46	89	1.2
69	31	-0.49	38	-0.32	67	0.42	82	0.9	93	1.46	89	1.2
70	31	-0.51	36	-0.36	64	0.36	82	0.9	93	1.43	89	1.2
71	29	-0.56	35	-0.38	62	0.3	81	0.89	92	1.39	89	1.2
72	27	-0.61	35	-0.39	61	0.27	80	0.85	91	1.36	89	1.2
73	25	-0.67	34	-0.42	60	0.24	79	0.81	91	1.34	89	1.2
74	23	-0.75	31	-0.49	59	0.22	78	0.77	91	1.32	89	1.2

Table S16. Percentile ranks and *z*-scores per age group for the Trail Making Test, Part B (≥ 12 years of education).

Test score (sec)	Age (years)											
	18-29 (*n* = 59)		30-49 (*n* = 123)		50-59 (*n* = 103)		60-69 (*n* = 142)		70-79 (*n* = 140)		≥ 80 (*n* = 44)	
	PR	*z*-score	PR	*z*-score	PR	*z*-score	PR	*z*-score	PR	*z*-score	PR	*z*-score
75	22	-0.78	30	-0.53	58	0.19	77	0.74	90	1.26	89	1.2
76	22	-0.78	29	-0.56	57	0.18	76	0.7	89	1.2	89	1.2
77	22	-0.78	28	-0.6	57	0.17	75	0.67	89	1.2	89	1.2
78	21	-0.8	27	-0.62	55	0.12	75	0.66	89	1.2	89	1.2
79	20	-0.83	25	-0.67	53	0.08	74	0.65	89	1.2	89	1.2
80	20	-0.83	24	-0.7	53	0.07	73	0.62	89	1.2	89	1.2
81	20	-0.83	24	-0.7	51	0.03	71	0.56	88	1.18	89	1.2
82	19	-0.9	24	-0.7	50	0	69	0.5	88	1.15	89	1.2
83	16	-1	24	-0.71	50	-0.02	68	0.46	87	1.11	88	1.15
84	15	-1.03	24	-0.72	48	-0.05	67	0.43	86	1.08	86	1.09
85	15	-1.03	23	-0.75	45	-0.14	65	0.38	86	1.06	86	1.09
86	15	-1.03	22	-0.79	42	-0.2	64	0.36	85	1.05	86	1.09
87	15	-1.03	21	-0.82	41	-0.23	63	0.32	85	1.03	85	1.04
88	14	-1.07	20	-0.85	40	-0.26	59	0.22	85	1.03	84	0.99
89	14	-1.11	20	-0.86	37	-0.33	55	0.13	85	1.03	83	0.95
90	13	-1.14	19	-0.88	35	-0.38	54	0.09	85	1.03	80	0.82
91	12	-1.19	18	-0.92	35	-0.39	54	0.08	85	1.03	77	0.74
92	12	-1.19	17	-0.97	35	-0.39	54	0.08	84	0.99	77	0.74
93	12	-1.19	15	-1.04	35	-0.39	53	0.07	82	0.92	77	0.74

Table S16. Percentile ranks and *z*-scores per age group for the Trail Making Test, Part B (≥ 12 years of education).

Test score (sec)	Age (years)											
	18-29 (*n* = 59)		30-49 (*n* = 123)		50-59 (*n* = 103)		60-69 (*n* = 142)		70-79 (*n* = 140)		≥ 80 (*n* = 44)	
	PR	*z*-score	PR	*z*-score	PR	*z*-score	PR	*z*-score	PR	*z*-score	PR	*z*-score
94	12	-1.19	13	-1.11	35	-0.39	53	0.07	81	0.88	77	0.74
95	12	-1.19	13	-1.15	34	-0.4	51	0.03	80	0.82	77	0.74
96	10	-1.28	12	-1.17	33	-0.43	50	0	79	0.79	76	0.71
97	8	-1.38	12	-1.19	33	-0.46	50	0	78	0.77	75	0.67
98	8	-1.38	11	-1.21	32	-0.47	49	-0.02	78	0.75	75	0.67
99	8	-1.38	11	-1.21	32	-0.47	49	-0.04	77	0.73	75	0.67
100	8	-1.38	11	-1.21	31	-0.5	49	-0.04	76	0.69	72	0.57
101	8	-1.38	11	-1.21	29	-0.55	49	-0.04	75	0.66	68	0.47
102	8	-1.38	11	-1.23	28	-0.58	48	-0.06	73	0.61	68	0.47
103	8	-1.38	10	-1.28	28	-0.58	47	-0.08	71	0.56	68	0.47
104	8	-1.38	10	-1.3	28	-0.58	46	-0.09	70	0.53	68	0.47
105	7	-1.5	10	-1.3	28	-0.6	46	-0.1	69	0.5	68	0.47
106	5	-1.64	10	-1.3	27	-0.61	46	-0.11	68	0.45	68	0.47
107	5	-1.64	10	-1.3	27	-0.61	45	-0.13	66	0.42	68	0.47
108	5	-1.64	10	-1.3	27	-0.63	44	-0.15	66	0.42	68	0.47
109	5	-1.64	10	-1.3	25	-0.67	44	-0.15	66	0.41	68	0.47
110	5	-1.64	10	-1.3	24	-0.72	44	-0.16	65	0.39	68	0.47
111	5	-1.64	10	-1.3	23	-0.73	43	-0.17	65	0.38	68	0.47
112	5	-1.64	10	-1.3	23	-0.73	43	-0.19	65	0.37	68	0.47

Table S16. Percentile ranks and *z*-scores per age group for the Trail Making Test, Part B (≥ 12 years of education).

Test score (sec)	Age (years)											
	18-29 (*n* = 59)		30-49 (*n* = 123)		50-59 (*n* = 103)		60-69 (*n* = 142)		70-79 (*n* = 140)		≥ 80 (*n* = 44)	
	PR	*z*-score	PR	*z*-score	PR	*z*-score	PR	*z*-score	PR	*z*-score	PR	*z*-score
113	5	-1.64	10	-1.3	23	-0.73	42	-0.21	63	0.33	68	0.47
114	5	-1.64	9	-1.32	23	-0.75	42	-0.22	62	0.3	66	0.41
115	4	-1.73	9	-1.35	21	-0.8	41	-0.23	61	0.27	64	0.34
116	3	-1.83	9	-1.35	20	-0.83	41	-0.24	59	0.23	64	0.34
117	3	-1.83	9	-1.35	20	-0.83	40	-0.25	58	0.2	63	0.31
118	3	-1.96	9	-1.35	20	-0.83	39	-0.27	58	0.19	61	0.28
119	2	-2.13	9	-1.35	20	-0.83	38	-0.31	58	0.19	61	0.28
120	2	-2.13	9	-1.35	20	-0.85	37	-0.34	57	0.17	61	0.28
121	2	-2.13	9	-1.35	19	-0.87	37	-0.35	55	0.12	61	0.28
122	2	-2.13	9	-1.37	18	-0.9	37	-0.35	54	0.09	61	0.28
123	1	-2.39	8	-1.43	17	-0.96	37	-0.35	53	0.08	61	0.28
124	0	-3	7	-1.46	17	-0.98	36	-0.36	53	0.07	59	0.22
125	0	-3	7	-1.46	17	-0.98	36	-0.38	52	0.05	57	0.17
126	0	-3	7	-1.46	16	-1	35	-0.38	51	0.01	57	0.17
127	0	-3	7	-1.49	16	-1.02	35	-0.38	50	0	57	0.17
128	0	-3	7	-1.52	15	-1.04	35	-0.38	50	0	57	0.17
129	0	-3	7	-1.52	15	-1.06	35	-0.38	50	-0.01	57	0.17
130	0	-3	6	-1.55	14	-1.08	35	-0.4	49	-0.04	57	0.17
131	0	-3	6	-1.59	14	-1.1	34	-0.42	48	-0.06	57	0.17

Table S16. Percentile ranks and *z*-scores per age group for the Trail Making Test, Part B (≥ 12 years of education).

Test score (sec)	Age (years)											
	18-29 (*n* = 59)		30-49 (*n* = 123)		50-59 (*n* = 103)		60-69 (*n* = 142)		70-79 (*n* = 140)		≥ 80 (*n* = 44)	
	PR	*z*-score	PR	*z*-score	PR	*z*-score	PR	*z*-score	PR	*z*-score	PR	*z*-score
132	0	-3	6	-1.59	14	-1.1	33	-0.43	48	-0.06	57	0.17
133	0	-3	6	-1.59	14	-1.1	33	-0.44	48	-0.07	57	0.17
134	0	-3	6	-1.59	14	-1.1	33	-0.45	46	-0.09	57	0.17
135	0	-3	6	-1.59	14	-1.1	32	-0.46	46	-0.11	57	0.17
136	0	-3	6	-1.59	14	-1.1	32	-0.47	46	-0.11	56	0.14
137	0	-3	6	-1.59	13	-1.13	32	-0.48	45	-0.12	55	0.11
138	0	-3	6	-1.59	13	-1.15	32	-0.48	45	-0.13	55	0.11
139	0	-3	6	-1.59	13	-1.15	31	-0.5	45	-0.14	55	0.11
140	0	-3	6	-1.59	12	-1.17	30	-0.54	44	-0.15	55	0.11
141	0	-3	6	-1.59	12	-1.2	28	-0.58	44	-0.15	55	0.11
142	0	-3	5	-1.62	11	-1.22	27	-0.6	44	-0.15	55	0.11
143	0	-3	4	-1.7	11	-1.25	27	-0.62	44	-0.15	55	0.11
144	0	-3	4	-1.75	11	-1.25	26	-0.66	44	-0.15	55	0.11
145	0	-3	4	-1.8	11	-1.25	25	-0.67	44	-0.15	55	0.11
146	0	-3	3	-1.85	11	-1.25	25	-0.68	44	-0.16	55	0.11
147	0	-3	3	-1.85	11	-1.25	25	-0.69	44	-0.17	55	0.11
148	0	-3	3	-1.85	11	-1.25	24	-0.71	43	-0.18	55	0.11
149	0	-3	3	-1.85	11	-1.25	23	-0.75	42	-0.2	55	0.11
150	0	-3	3	-1.85	11	-1.25	23	-0.76	42	-0.21	55	0.11

Table S16. Percentile ranks and *z*-scores per age group for the Trail Making Test, Part B (≥ 12 years of education).

Test score (sec)	Age (years)											
	18-29 (*n* = 59)		30-49 (*n* = 123)		50-59 (*n* = 103)		60-69 (*n* = 142)		70-79 (*n* = 140)		≥ 80 (*n* = 44)	
	PR	*z*-score	PR	*z*-score	PR	*z*-score	PR	*z*-score	PR	*z*-score	PR	*z*-score
151	0	-3	3	-1.85	11	-1.25	23	-0.76	41	-0.22	55	0.11
152	0	-3	3	-1.85	11	-1.25	23	-0.76	41	-0.22	55	0.11
153	0	-3	3	-1.85	11	-1.25	23	-0.76	41	-0.22	53	0.08
154	0	-3	3	-1.85	11	-1.25	23	-0.76	41	-0.24	52	0.05
155	0	-3	3	-1.85	11	-1.25	23	-0.76	39	-0.28	51	0.02
156	0	-3	3	-1.85	11	-1.25	23	-0.76	38	-0.31	50	0
157	0	-3	3	-1.85	11	-1.25	23	-0.76	37	-0.33	50	0
158	0	-3	3	-1.85	11	-1.25	22	-0.78	36	-0.35	50	0
159	0	-3	3	-1.85	11	-1.25	21	-0.81	36	-0.37	50	0
160	0	-3	3	-1.85	10	-1.28	21	-0.82	36	-0.37	49	-0.03
161	0	-3	3	-1.85	10	-1.3	20	-0.84	36	-0.37	47	-0.09
162	0	-3	3	-1.85	10	-1.3	20	-0.86	35	-0.38	45	-0.12
163	0	-3	3	-1.85	10	-1.3	20	-0.86	35	-0.39	45	-0.12
164	0	-3	3	-1.85	9	-1.33	19	-0.88	35	-0.39	45	-0.12
165	0	-3	3	-1.85	8	-1.39	18	-0.92	34	-0.41	45	-0.12
166	0	-3	3	-1.85	8	-1.43	18	-0.94	34	-0.43	45	-0.12
167	0	-3	3	-1.85	8	-1.43	18	-0.94	33	-0.45	45	-0.12
168	0	-3	3	-1.85	7	-1.46	18	-0.94	31	-0.49	45	-0.12
169	0	-3	3	-1.85	7	-1.5	18	-0.94	30	-0.53	45	-0.12

Table S16. Percentile ranks and *z*-scores per age group for the Trail Making Test, Part B (≥ 12 years of education).

Test score (sec)	Age (years)											
	18-29 (*n* = 59)		30-49 (*n* = 123)		50-59 (*n* = 103)		60-69 (*n* = 142)		70-79 (*n* = 140)		≥ 80 (*n* = 44)	
	PR	*z*-score	PR	*z*-score	PR	*z*-score	PR	*z*-score	PR	*z*-score	PR	*z*-score
170	0	-3	3	-1.85	7	-1.5	18	-0.94	29	-0.56	45	-0.12
171	0	-3	3	-1.85	7	-1.5	17	-0.95	29	-0.57	45	-0.12
172	0	-3	3	-1.85	7	-1.5	17	-0.96	29	-0.57	45	-0.12
173	0	-3	3	-1.85	7	-1.5	17	-0.96	29	-0.57	45	-0.12
174	0	-3	3	-1.85	7	-1.5	17	-0.96	29	-0.57	45	-0.12
175	0	-3	3	-1.85	6	-1.53	17	-0.96	28	-0.58	44	-0.15
176	0	-3	3	-1.85	6	-1.57	17	-0.96	28	-0.59	43	-0.18
177	0	-3	3	-1.85	6	-1.57	17	-0.96	28	-0.6	43	-0.18
178	0	-3	3	-1.85	6	-1.57	17	-0.96	27	-0.61	43	-0.18
179	0	-3	3	-1.85	5	-1.62	17	-0.98	27	-0.62	43	-0.18
180	0	-3	3	-1.85	4	-1.71	16	-1.01	26	-0.63	41	-0.23
181	0	-3	3	-1.85	4	-1.77	15	-1.02	26	-0.63	39	-0.29
182	0	-3	3	-1.85	4	-1.77	15	-1.02	26	-0.65	39	-0.29
183	0	-3	3	-1.85	4	-1.77	15	-1.04	26	-0.66	39	-0.29
184	0	-3	3	-1.85	4	-1.77	15	-1.05	25	-0.67	39	-0.29
185	0	-3	3	-1.85	4	-1.77	15	-1.05	25	-0.68	39	-0.29
186	0	-3	3	-1.85	4	-1.77	15	-1.05	25	-0.68	39	-0.29
187	0	-3	3	-1.85	4	-1.77	15	-1.05	25	-0.68	39	-0.29
188	0	-3	3	-1.85	4	-1.77	15	-1.05	25	-0.69	39	-0.29

Table S16. Percentile ranks and *z*-scores per age group for the Trail Making Test, Part B (≥ 12 years of education).

Test score (sec)	Age (years)											
	18-29 (*n* = 59)		30-49 (*n* = 123)		50-59 (*n* = 103)		60-69 (*n* = 142)		70-79 (*n* = 140)		≥ 80 (*n* = 44)	
	PR	*z*-score	PR	*z*-score	PR	*z*-score	PR	*z*-score	PR	*z*-score	PR	*z*-score
189	0	-3	3	-1.85	4	-1.77	14	-1.07	24	-0.7	39	-0.29
190	0	-3	3	-1.85	4	-1.77	14	-1.08	24	-0.71	39	-0.29
191	0	-3	3	-1.85	4	-1.77	14	-1.1	24	-0.73	39	-0.29
192	0	-3	3	-1.85	4	-1.77	13	-1.11	24	-0.73	39	-0.29
193	0	-3	3	-1.85	4	-1.77	13	-1.11	23	-0.74	38	-0.32
194	0	-3	3	-1.85	4	-1.77	13	-1.13	23	-0.76	36	-0.35
195	0	-3	3	-1.85	4	-1.77	12	-1.16	22	-0.77	36	-0.35
196	0	-3	3	-1.85	4	-1.77	12	-1.18	22	-0.77	36	-0.35
197	0	-3	3	-1.85	4	-1.77	12	-1.18	22	-0.78	36	-0.35
198	0	-3	3	-1.85	4	-1.77	12	-1.18	21	-0.8	36	-0.35
199	0	-3	3	-1.85	4	-1.77	12	-1.18	21	-0.8	36	-0.35
200	0	-3	3	-1.85	3	-1.83	12	-1.18	21	-0.8	36	-0.35
201	0	-3	3	-1.85	3	-1.9	12	-1.18	21	-0.8	36	-0.35
202	0	-3	3	-1.85	3	-1.9	12	-1.18	21	-0.81	36	-0.35
203	0	-3	3	-1.85	3	-1.9	12	-1.18	20	-0.83	35	-0.38
204	0	-3	3	-1.85	3	-1.9	12	-1.18	20	-0.86	34	-0.41
205	0	-3	3	-1.85	3	-1.9	12	-1.18	19	-0.89	34	-0.41
206	0	-3	3	-1.85	3	-1.9	12	-1.18	19	-0.9	34	-0.41
207	0	-3	3	-1.91	3	-1.9	12	-1.18	19	-0.9	34	-0.41

Table S16. Percentile ranks and *z*-scores per age group for the Trail Making Test, Part B (≥ 12 years of education).

Test score (sec)	Age (years)											
	18-29 (*n* = 59)		30-49 (*n* = 123)		50-59 (*n* = 103)		60-69 (*n* = 142)		70-79 (*n* = 140)		≥ 80 (*n* = 44)	
	PR	*z*-score	PR	*z*-score	PR	*z*-score	PR	*z*-score	PR	*z*-score	PR	*z*-score
208	0	-3	2	-1.97	3	-1.9	12	-1.18	19	-0.9	34	-0.41
209	0	-3	2	-2.05	3	-1.9	12	-1.18	19	-0.9	34	-0.41
210	0	-3	2	-2.14	2	-1.98	12	-1.18	18	-0.94	32	-0.48
211	0	-3	2	-2.14	2	-2.07	12	-1.18	16	-0.98	30	-0.54
212	0	-3	2	-2.14	2	-2.07	12	-1.18	16	-0.98	30	-0.54
213	0	-3	2	-2.14	2	-2.07	12	-1.18	16	-0.98	30	-0.54
214	0	-3	1	-2.25	2	-2.07	12	-1.2	16	-0.98	30	-0.54
215	0	-3	1	-2.41	2	-2.07	11	-1.25	16	-0.98	30	-0.54
216	0	-3	1	-2.41	2	-2.07	10	-1.29	16	-1	30	-0.54
217	0	-3	1	-2.41	2	-2.07	10	-1.29	16	-1.01	30	-0.54
218	0	-3	1	-2.41	2	-2.07	10	-1.29	16	-1.01	30	-0.54
219	0	-3	1	-2.41	2	-2.07	10	-1.29	15	-1.03	30	-0.54
220	0	-3	1	-2.41	2	-2.07	10	-1.29	15	-1.06	30	-0.54
221	0	-3	1	-2.41	2	-2.07	10	-1.29	14	-1.07	30	-0.54
222	0	-3	1	-2.41	2	-2.07	10	-1.29	14	-1.07	30	-0.54
223	0	-3	1	-2.41	2	-2.07	10	-1.29	14	-1.07	30	-0.54
224	0	-3	1	-2.41	2	-2.07	10	-1.29	14	-1.07	30	-0.54
225	0	-3	1	-2.41	2	-2.07	10	-1.29	14	-1.1	30	-0.54
226	0	-3	1	-2.41	2	-2.07	10	-1.29	13	-1.14	30	-0.54

Table S16. Percentile ranks and *z*-scores per age group for the Trail Making Test, Part B (≥ 12 years of education).

Test score (sec)	Age (years)											
	18-29 (*n* = 59)		30-49 (*n* = 123)		50-59 (*n* = 103)		60-69 (*n* = 142)		70-79 (*n* = 140)		≥ 80 (*n* = 44)	
	PR	*z*-score	PR	*z*-score	PR	*z*-score	PR	*z*-score	PR	*z*-score	PR	*z*-score
227	0	-3	1	-2.41	2	-2.07	10	-1.31	13	-1.14	30	-0.54
228	0	-3	1	-2.41	2	-2.07	9	-1.34	13	-1.14	30	-0.54
229	0	-3	1	-2.41	2	-2.07	9	-1.34	13	-1.14	27	-0.61
230	0	-3	1	-2.41	2	-2.07	9	-1.34	13	-1.14	25	-0.68
231	0	-3	1	-2.41	2	-2.07	9	-1.36	13	-1.14	25	-0.68
232	0	-3	1	-2.41	2	-2.07	8	-1.38	13	-1.14	25	-0.68
233	0	-3	1	-2.41	2	-2.07	8	-1.38	13	-1.14	25	-0.68
234	0	-3	1	-2.41	2	-2.07	8	-1.38	13	-1.14	25	-0.68
235	0	-3	1	-2.41	2	-2.07	8	-1.38	13	-1.14	25	-0.68
236	0	-3	1	-2.41	2	-2.07	8	-1.38	12	-1.17	25	-0.68
237	0	-3	1	-2.41	2	-2.07	8	-1.38	11	-1.21	25	-0.68
238	0	-3	1	-2.41	2	-2.07	8	-1.38	11	-1.21	25	-0.68
239	0	-3	1	-2.41	2	-2.07	8	-1.38	11	-1.21	25	-0.68
240	0	-3	1	-2.41	1	-2.18	8	-1.43	11	-1.25	25	-0.68
241	0	-3	1	-2.41	1	-2.34	7	-1.48	10	-1.29	25	-0.68
242	0	-3	1	-2.41	1	-2.34	7	-1.48	10	-1.29	25	-0.68
243	0	-3	1	-2.41	1	-2.34	7	-1.48	10	-1.29	25	-0.68
244	0	-3	1	-2.41	1	-2.34	7	-1.48	10	-1.29	25	-0.68
245	0	-3	1	-2.41	1	-2.34	7	-1.48	10	-1.29	25	-0.68

Table S16. Percentile ranks and *z*-scores per age group for the Trail Making Test, Part B (≥ 12 years of education).

Test score (sec)	Age (years)											
	18-29 (*n* = 59)		30-49 (*n* = 123)		50-59 (*n* = 103)		60-69 (*n* = 142)		70-79 (*n* = 140)		≥ 80 (*n* = 44)	
	PR	*z*-score	PR	*z*-score	PR	*z*-score	PR	*z*-score	PR	*z*-score	PR	*z*-score
246	0	-3	1	-2.41	1	-2.34	7	-1.5	10	-1.29	24	-0.72
247	0	-3	1	-2.41	1	-2.34	6	-1.53	10	-1.29	23	-0.75
248	0	-3	1	-2.41	1	-2.34	6	-1.53	10	-1.29	23	-0.75
249	0	-3	1	-2.41	1	-2.34	6	-1.53	10	-1.31	23	-0.75
250	0	-3	1	-2.41	1	-2.34	6	-1.53	9	-1.33	23	-0.75
251	0	-3	1	-2.41	1	-2.34	6	-1.53	9	-1.37	23	-0.75
252	0	-3	1	-2.41	1	-2.34	6	-1.53	8	-1.42	23	-0.75
253	0	-3	1	-2.41	1	-2.34	6	-1.53	8	-1.44	22	-0.79
254	0	-3	1	-2.41	1	-2.34	6	-1.56	7	-1.5	20	-0.83
255	0	-3	1	-2.41	1	-2.34	6	-1.59	6	-1.52	20	-0.83
256	0	-3	1	-2.41	1	-2.34	6	-1.59	6	-1.52	20	-0.83
257	0	-3	1	-2.41	1	-2.34	6	-1.59	6	-1.52	20	-0.83
258	0	-3	1	-2.41	1	-2.34	6	-1.59	6	-1.52	20	-0.83
259	0	-3	1	-2.41	1	-2.34	5	-1.62	6	-1.52	20	-0.83
260	0	-3	1	-2.41	1	-2.34	5	-1.69	6	-1.55	20	-0.83
261	0	-3	1	-2.41	1	-2.34	4	-1.73	6	-1.58	20	-0.83
262	0	-3	1	-2.41	1	-2.34	4	-1.73	6	-1.58	20	-0.83
263	0	-3	1	-2.41	1	-2.34	4	-1.77	6	-1.58	20	-0.83
264	0	-3	1	-2.41	1	-2.34	4	-1.81	6	-1.58	20	-0.83

Table S16. Percentile ranks and *z*-scores per age group for the Trail Making Test, Part B (≥ 12 years of education).

Test score (sec)	Age (years)											
	18-29 (*n* = 59)		30-49 (*n* = 123)		50-59 (*n* = 103)		60-69 (*n* = 142)		70-79 (*n* = 140)		≥ 80 (*n* = 44)	
	PR	*z*-score	PR	*z*-score	PR	*z*-score	PR	*z*-score	PR	*z*-score	PR	*z*-score
265	0	-3	1	-2.41	1	-2.34	4	-1.81	6	-1.58	20	-0.83
266	0	-3	1	-2.41	1	-2.34	4	-1.81	6	-1.58	20	-0.83
267	0	-3	1	-2.41	1	-2.34	4	-1.81	6	-1.58	20	-0.83
268	0	-3	1	-2.41	1	-2.34	4	-1.81	6	-1.58	20	-0.83
269	0	-3	1	-2.41	1	-2.34	4	-1.81	6	-1.58	20	-0.83
270	0	-3	1	-2.41	1	-2.34	4	-1.81	6	-1.58	20	-0.83
271	0	-3	1	-2.41	1	-2.34	4	-1.81	6	-1.58	20	-0.83
272	0	-3	1	-2.41	1	-2.34	3	-1.86	6	-1.58	20	-0.83
273	0	-3	1	-2.41	1	-2.34	3	-1.91	6	-1.58	20	-0.83
274	0	-3	1	-2.41	1	-2.34	3	-1.91	6	-1.58	20	-0.83
275	0	-3	1	-2.41	1	-2.34	3	-1.91	6	-1.58	20	-0.83
276	0	-3	1	-2.41	1	-2.34	3	-1.91	5	-1.62	20	-0.83
277	0	-3	1	-2.41	1	-2.34	3	-1.91	5	-1.65	20	-0.83
278	0	-3	1	-2.41	1	-2.34	3	-1.91	5	-1.65	20	-0.83
279	0	-3	1	-2.41	1	-2.34	3	-1.91	5	-1.65	20	-0.83
280	0	-3	1	-2.41	1	-2.34	3	-1.91	5	-1.69	20	-0.83
281	0	-3	1	-2.41	1	-2.34	3	-1.91	4	-1.72	20	-0.83
282	0	-3	1	-2.41	1	-2.34	3	-1.91	4	-1.72	20	-0.83
283	0	-3	1	-2.41	1	-2.34	3	-1.91	4	-1.72	20	-0.83

Table S16. Percentile ranks and *z*-scores per age group for the Trail Making Test, Part B (≥ 12 years of education).

Test score (sec)	Age (years)											
	18-29 (*n* = 59)		30-49 (*n* = 123)		50-59 (*n* = 103)		60-69 (*n* = 142)		70-79 (*n* = 140)		≥ 80 (*n* = 44)	
	PR	*z*-score	PR	*z*-score	PR	*z*-score	PR	*z*-score	PR	*z*-score	PR	*z*-score
284	0	-3	1	-2.41	1	-2.34	3	-1.91	4	-1.72	20	-0.83
285	0	-3	1	-2.41	1	-2.34	3	-1.91	4	-1.72	20	-0.83
286	0	-3	1	-2.41	1	-2.34	3	-1.91	4	-1.72	20	-0.83
287	0	-3	1	-2.41	1	-2.34	3	-1.91	4	-1.72	20	-0.83
288	0	-3	1	-2.41	1	-2.34	3	-1.91	4	-1.72	20	-0.83
289	0	-3	1	-2.41	1	-2.34	3	-1.91	4	-1.72	20	-0.83
290	0	-3	1	-2.41	1	-2.34	3	-1.91	4	-1.72	20	-0.83
291	0	-3	1	-2.41	1	-2.34	3	-1.91	4	-1.72	20	-0.83
292	0	-3	1	-2.41	1	-2.34	3	-1.91	4	-1.72	20	-0.83
293	0	-3	1	-2.41	1	-2.34	3	-1.91	4	-1.72	20	-0.83
294	0	-3	1	-2.41	1	-2.34	3	-1.91	4	-1.72	20	-0.83
295	0	-3	1	-2.41	1	-2.34	2	-1.97	4	-1.72	20	-0.83
296	0	-3	1	-2.41	1	-2.34	2	-2.04	4	-1.72	18	-0.91
297	0	-3	1	-2.41	1	-2.34	2	-2.04	4	-1.72	16	-1
298	0	-3	1	-2.41	1	-2.34	2	-2.04	4	-1.72	16	-1
299	0	-3	1	-2.41	1	-2.34	2	-2.04	4	-1.72	15	-1.05
300	0	-3	0	-2.64	0	-2.58	1	-2.31	2	-2.03	7	-1.49

White cells correspond to cognitive impairment. cells highlighted in gray represent normal cognition; **PR** Percentile rank.
